# Molecular engineering enabling reversible transformation between helical and planar conformations by cyclization of alkynes†

**DOI:** 10.1039/d0sc05844k

**Published:** 2020-12-08

**Authors:** Lipeng Yan, Weixin Ma, Jingbo Lan, Hu Cheng, Zhengyang Bin, Di Wu, Jingsong You

**Affiliations:** Key Laboratory of Green Chemistry and Technology of Ministry of Education, College of Chemistry, Sichuan University 29 Wangjiang Road Chengdu 610064 People's Republic of China jingbolan@scu.edu.cn jsyou@scu.edu.cn

## Abstract

Molecular engineering enabling reversible transformation between helical and planar conformations is described herein. Starting from easily available 2-(pyridin-2-yl)anilines and alkynes, a one-pot strategy is set up for the synthesis of aza[4]helicenes *via* two successive rhodium-catalyzed C–H activation/cyclizations. Helical pyrrolophenanthridiziniums can be transformed into planar conformations through the cleavage of acidic pyrrole N–H, leading to turn-off fluorescence. NMR spectra, single crystal X-ray diffraction and DFT calculations demonstrate that the formation of an intramolecular C–H⋯N hydrogen bond is beneficial to stabilize the pyrrole nitrogen anion of the planar molecule and provide increased planarity. The reversible conformation transformations can be finely adjusted by the electron-donating and -withdrawing groups on the π^+^-fused pyrrole skeleton in the physiological pH range, thus affording an opportunity for pH-controlled intracellular selective fluorescence imaging. Pyrrolophenanthridiziniums show turn-on fluorescence in lysosomes owing to the acidic environment of lysosomes and turn-off fluorescence out of lysosomes, indicating the occurrence of the deprotonation reaction outside lysosomes and the corresponding transformation from helical to planar conformations.

## Abstract 2

One-pot synthesis of aza[4]helicenes is accomplished through two successive C–H activation/cyclizations, which exhibit on/off fluorescence switching through reversible transformation between helical and planar conformations.

## Graphical abstract

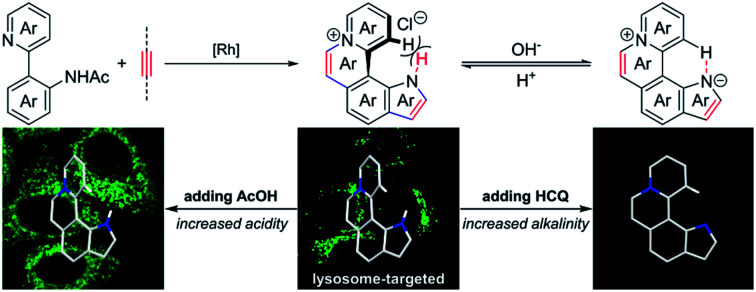

## Introduction

**Scheme 1:**
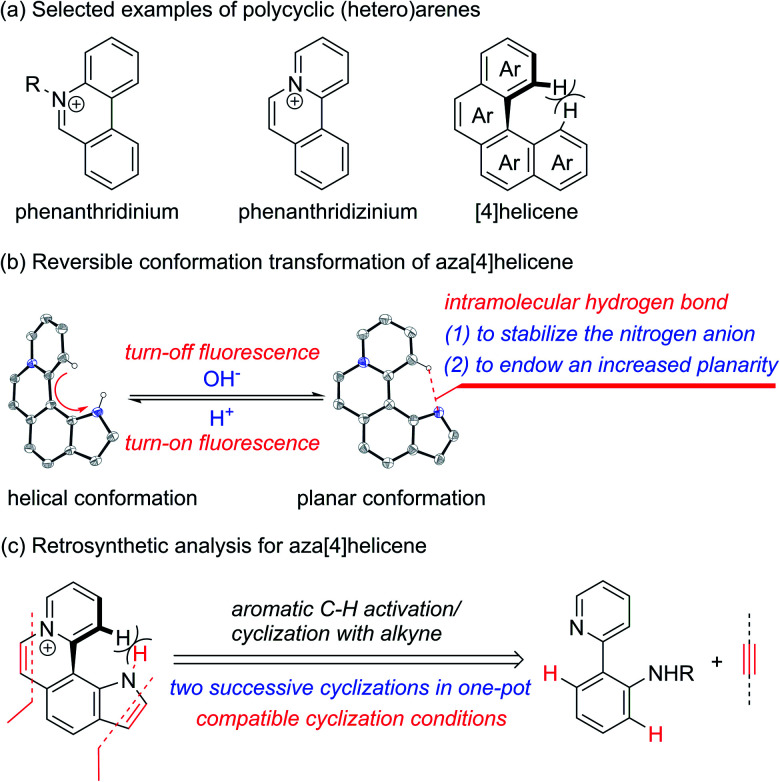
(a) Selected examples of polycyclic (hetero)arenes. (b) Reversible conformation transformation of aza[4]helicene. (c) Retrosynthetic analysis for aza[4]helicene.

Cationic polycyclic heteroaromatic (π^+^) compounds, such as phenanthridiniums and phenanthridiziniums, have been widely applied in many fields, such as fluorescence bioimaging, DNA binding, and photodynamic therapy (Scheme 1a).^1^ However, polycyclic (hetero)arenes with a large conjugated plane generally encounter aggregation-caused quenching (ACQ) due to strong π–π stacking interactions, leading to undesired weak emissions.^2^ Helicenes, constituted by *ortho*-fused (hetero)aromatic rings, possess screw-shaped three-dimensional conjugated skeletons, which may prevent intermolecular close packing, thus having an advantage of inhibiting ACQ.^3,4^

The nonplanar conformation of helicenes is attributed to the steric repulsion of terminal (hetero)aromatic rings and their functional groups.^3*a*^ [4]Helicene is the smallest helical π-conjugated molecule, and the electrostatic repulsion between two hydrogen atoms on its terminal aromatic rings results in the helical structure (Scheme 1a).^5^ Thus, we proposed that [4]helicene with an acidic proton on the terminal aromatic ring would undergo a transformation from helical to planar conformations *via* removing the terminal acidic hydrogen in an alkaline environment, thus enabling on/off fluorescence switching. However, the design and construction of [4]helicene with an acidic proton on the terminal aromatic ring remain challenging due to the large p*K*_a_ values (>20) of aromatic C–H.^6^ The N–H of pyrrole is weakly acidic, but its p*K*_a_ value is still relatively large (approximately 23).^6*a*,7^ It is a reasonable speculation that π^+^-fused pyrrole may have more strongly acidic N–H that can be removed easily due to the strong electron-withdrawing effect of π^+^, thus providing an opportunity to implement transformation between helical and planar conformations (Scheme 1b).

The existing methods for the synthesis of azahelicenes typically suffer from multistep reactions. Undoubtedly, π^+^-fused pyrroles with helical structures are not readily accessible by traditional synthetic methods. In recent years, transition metal-catalyzed direct C–H functionalization has emerged as one of the most important approaches for the construction of various π-conjugated bi(hetero)aryls and ring-fused structures.^8^ Apparently, two successive cyclizations of 2-(pyridin-2-yl)anilines with alkynes through pyridyl and amino (or amide) group-directed C–H activation would be an ideal approach to construct pyridinium (π^+^) and pyrrole rings (Scheme 1c). Moreover, if the two cyclization reactions can be accomplished in one pot, it would undoubtedly offer more efficient and step-economical synthetic access to π^+^-fused pyrroles with helical structures. However, the construction of both pyridinium and pyrrole rings in one pot remains challenging because these two reaction conditions are not easily compatible with each other.^9,10^ Herein, we wish to present the one-pot synthesis of pyrrolo[3,2-*k*]phenanthridiziniums with helical structures through two successive Rh-catalyzed cyclizations of 2-(pyridin-2-yl)anilines with alkynes. The resulting aza[4]helicenes exhibit relatively strong fluorescence emission and can be transformed into fluorescence-quenched planar structures in the pH range from 2.8 to 10.5 depending on the electron-donating and -withdrawing groups on the π^+^-fused pyrrole skeleton. The conformation transformation of most of the aza[4]helicenes occurs in the physiological pH range, thus opening a window for pH-controlled intracellular selective fluorescence imaging.

## Results and discussion

**Scheme 2:**
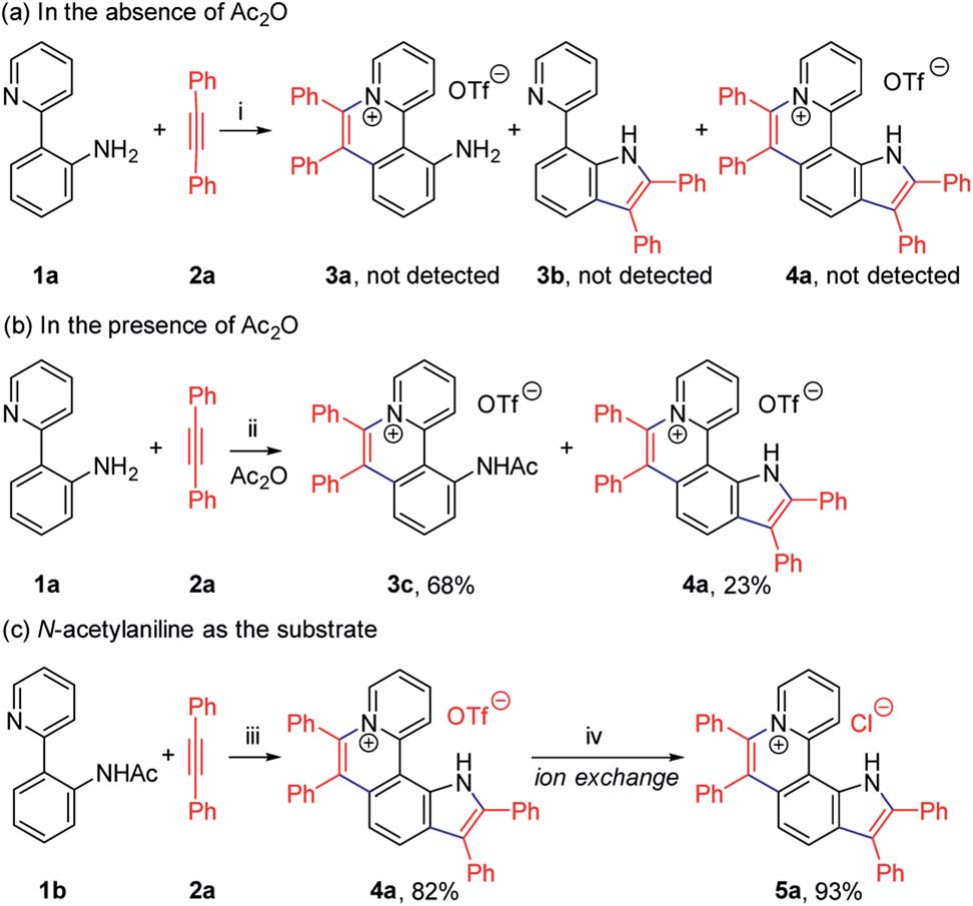
Rh-Catalyzed cyclization of 2-(pyridin-2-yl)aniline (1) with 1,2-diphenylacetylene (2a). Reaction conditions: (i) 1a (0.1 mmol), 2a (0.2 mmol), [Cp*RhCl_2_]_2_ (5 mol%), and AgOTf (0.12 mmol) in *t*-AmOH (1.5 mL) at 120 °C for 24 h under O_2_; (ii) 1a (0.1 mmol), 2a (0.2 mmol), [Cp*RhCl_2_]_2_ (5 mol%), AgOTf (0.12 mmol), and Ac_2_O (0.5 mmol) in *t*-AmOH (1.5 mL) at 120 °C for 24 h under O_2_; (iii) 1b (0.1 mmol), 2a (0.3 mmol), [Cp*RhCl_2_]_2_ (5 mol%), AgSbF_6_ (20 mol%), Cu(OAc)_2_·H_2_O (0.3 mmol) and TfOH (0.15 mmol) in *t*-AmOH (1.5 mL) at 140 °C for 24 h under N_2_; (iv) column chromatography on ion exchange resin. *t*-AmOH = 2-methylbutan-2-ol. AgOTf = silver trifluoromethanesulfonate. TfOH = trifluoromethanesulfonic acid.

To achieve sequential two-step cyclizations in one pot, the cyclization reaction conditions were explored initially by employing 2-(pyridin-2-yl)aniline (1a) and 1,2-diphenylacetylene (2a) as the substrates. Under the catalysis of [Cp*RhCl_2_]_2_ with AgOTf as an additive in an O_2_ atmosphere, no annulated product was observed (Scheme 2a). Upon addition of Ac_2_O to *in situ* generate *N*-acetyl-2-(pyridin-2-yl)aniline (1b), phenanthridizinium (3c) was obtained in 68% yield, accompanied by 2,3,6,7-tetraphenyl-1*H*-pyrrolo[3,2-*k*]phenanthridizinium (4a) in 23% yield (Scheme 2b and Section III in the ESI†). Thus, further optimization of reaction conditions was carried out by the direct use of 1b and 2a as the substrates (Table S1†). Finally, 4a was afforded in 82% yield in the presence of 5 mol% of [Cp*RhCl_2_]_2_, 20 mol% of AgSbF_6_, 3.0 equiv. of Cu(OAc)_2_·H_2_O as an oxidant, and 1.5 equiv. of trifluoromethanesulfonic acid (TfOH) as an additive in 2-methylbutan-2-ol (*t*-AmOH) at 140 °C for 24 h in a N_2_ atmosphere (Scheme 2c and Table S1,† entry 8). Considering that the poor water solubility of 4a would prevent its application in a physiological environment, pyrrolophenanthridizinium chloride (5a) was prepared by column chromatography on ion exchange resin (Scheme 2c).

**Fig. 1:**
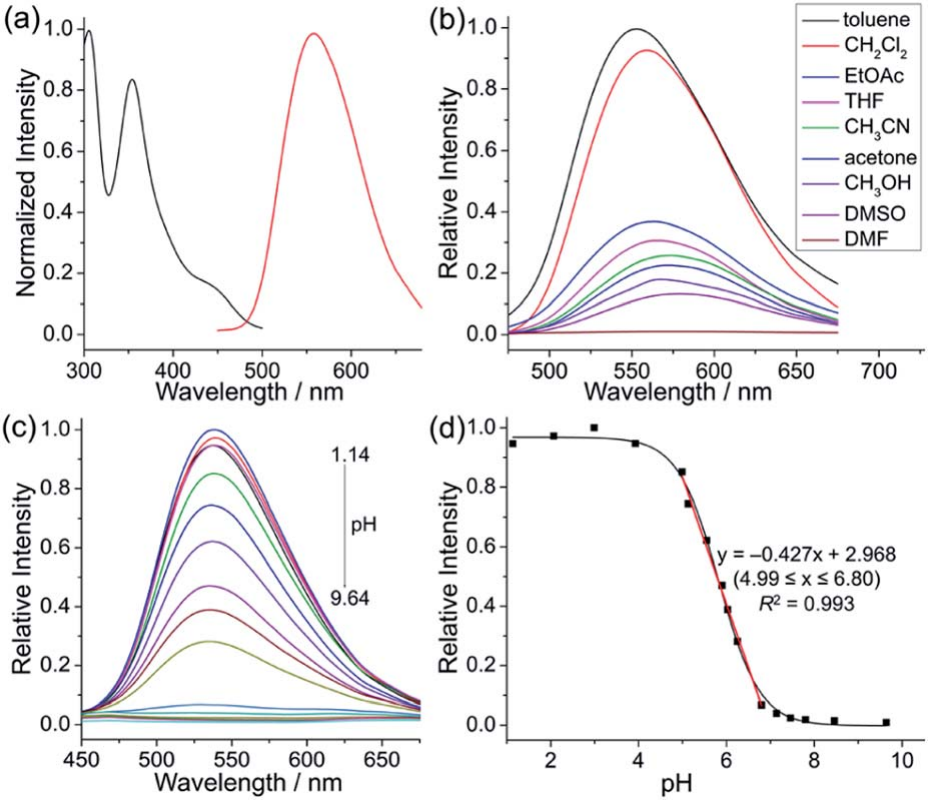
Photophysical properties of 5a. (a) Absorption spectrum (black) and fluorescence spectrum (red) of 5a in CH_2_Cl_2_ (1 × 10^−5^ M). (b) Fluorescence spectra of 5a in different solvents (1 × 10^−5^ M). (c) Fluorescence spectra of 5a in PBS solutions with different pH values (2 × 10^−5^ M, pH = 1.14–9.64, DMSO/H_2_O = 1 : 9, v/v). (d) Relative fluorescence intensities of 5a at 538 nm in phosphate-buffered saline with different pH values (2 × 10^−5^ M, pH = 1.14–9.64, DMSO/H_2_O = 1 : 9, v/v). Inset: The linear relationship between the relative fluorescence intensity of 5a at 538 nm and pH values ranging from 4.99 to 6.80.

Next, the photophysical properties of 5a were investigated. The UV-vis absorption spectrum of 5a in CH_2_Cl_2_ (1 × 10^−5^ M) exhibits two absorption bands with the maxima at 305 and 354 nm, which may be assigned to π–π* electronic transition of the π-conjugated backbone and charge-transfer (CT) transition from the electron-rich pyrrole unit to the electron-deficient pyridinium unit, respectively (Table 1 and Fig. 1a). The CH_2_Cl_2_ solution of 5a exhibits an emission peak at 559 nm with a large Stokes shift of 205 nm (Table 1 and Fig. 1a). As shown in Fig. 1b, the fluorescence intensity of 5a gradually decreased with the increase of solvent polarity from low-polarity toluene to high-polarity dimethyl sulfoxide (DMSO). Unexpectedly, pronounced fluorescence quenching is observed in *N*,*N*-dimethylformamide (DMF). It is inferred that basicity may cause the fluorescence quenching due to the presence of a small amount of dimethylamine in DMF. Inspired by the fact, fluorescence titration experiments were carried out in different pH environments (Fig. 1c). The emission maximum of 5a is located at 538 nm in acidic phosphate-buffered saline (PBS solution), which is gradually weakened with the increase of the pH value. A good linear correlation (*R*^2^ = 0.993) between emission intensity and the pH value is observed in the pH range from 4.99 to 6.80 (Fig. 1d). The p*K*_a_ value of 5.72 for 5a is in the physiological range, which thus provides an opportunity for pH-controlled intracellular selective fluorescence imaging.

**Table 1:** Synthesis, photophysical properties and pH ranges of aza[4]helicenes^a^

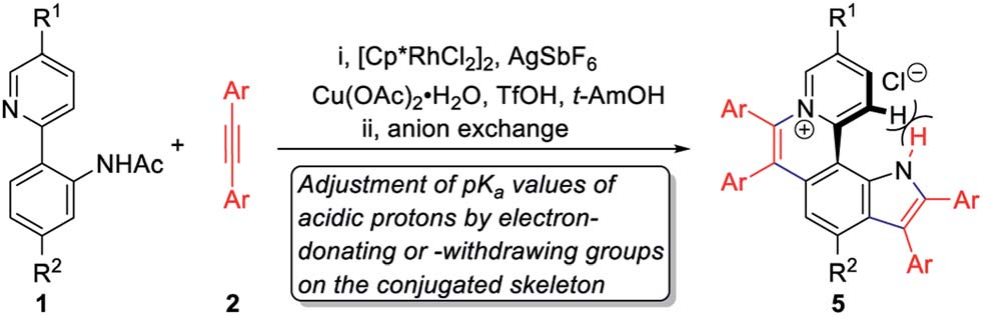									
Compound	Ar	R^1^	R^2^	Yield^b^ (%)	Abs^c^ (nm)	Em^d^ (nm)	Stokes shift (nm)	p*K*_a_	pH range^e^
5a	Ph	H	H	76	305, 354	559	205	5.72	4.99–6.80
5b	4-CH_3_C_6_H_4_	H	H	76	307, 358	579	221	5.83	4.96–6.98
5c	4-*t*-BuC_6_H_4_	H	H	72	308, 360	582	222	4.18	2.76–5.23
5d	4-FC_6_H_4_	H	H	66	305, 354	558	204	5.39	4.63–6.55
5e	4-CH_3_OC_6_H_4_	H	H	78	311, 365	517	152	8.76	7.07–10.54
5f	3-CH_3_OC_6_H_4_	H	H	58	299, 355	561	206	5.84	4.97–6.55
5g	Ph	H	OCH_3_	65	304, 345	552	207	6.11	5.27–6.85
5h	Ph	OCH_3_	H	61	303, 350	559	209	5.67	4.96–6.72
5i	Ph	OCH_3_	OCH_3_	56	302, 342	557	215	7.74	6.96–8.19
5j	Ph	CF_3_	H	63	318, 374	604	230	7.43	4.97–9.90
5k	Ph	CF_3_	OCH_3_	59	316, 354	581	227	6.96	5.58–8.34

a Reaction conditions: (i) 1 (0.1 mmol), 2 (0.3 mmol), [Cp*RhCl_2_]_2_ (5 mol%), AgSbF_6_ (20 mol%), Cu(OAc)_2_·H_2_O (3.0 equiv.) and TfOH (1.5 equiv.) in *t*-AmOH at 140 °C under N_2_ for 24 h; (ii) trifluoromethanesulfonate was exchanged to chloride *via* column chromatography on ion exchange resin.

b Isolated yields in two steps.

c Absorption maxima in CH_2_Cl_2_ (1 × 10^−5^ M).

d Emission maxima in CH_2_Cl_2_ (1 × 10^−5^ M).

e pH ranges where the fluorescence intensities of 5 vary linearly.

**Fig. 2:**
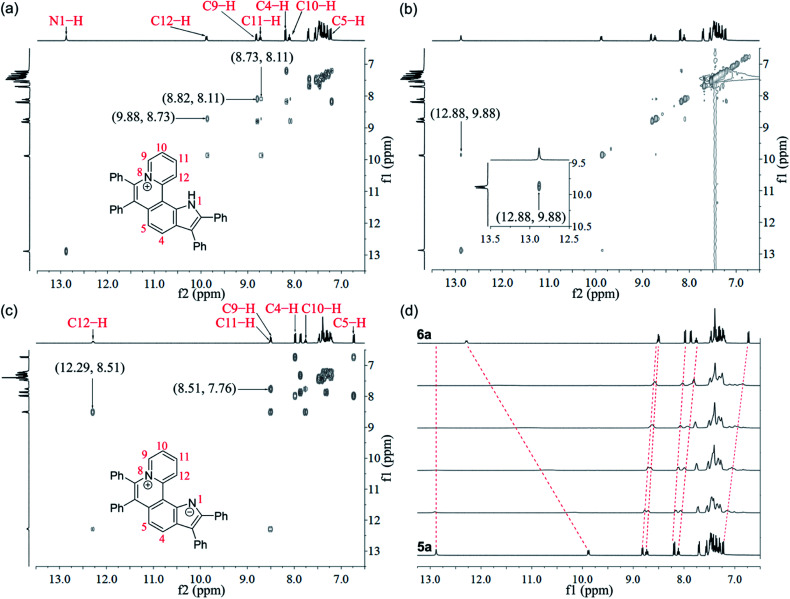
NMR spectra of 5a and 6a. (a) ^1^H–^1^H COSY spectrum of 5a. (b) ^1^H–^1^H NOESY spectrum of 5a. (c) ^1^H–^1^H COSY spectrum of 6a. (d) ^1^H NMR spectrum variation from 5a to 6a upon titration of NaOH solution (2 M in water, from bottom to top: 0 μL, 2 μL, 4 μL, 6 μL, 8 μL, and 16 μL) into 5a (16 μmol in DMSO-d_6_ solution) at room temperature.

Given the acidic N–H on the pyrrole ring of 5a, it is speculated that this proton could be deprived in an alkaline environment, resulting in a planar zwitterion structure (6a). To verify this hypothesis, 6a was prepared through the reaction of 5a (in CH_2_Cl_2_) with NaOH solution (2 M in water). Next, the ^1^H–^1^H NOESY and ^1^H–^1^H COSY NMR spectra of 5a and 6a were investigated to assign characteristic protons (Fig. 2a–c). ^1^H–^1^H NOESY NMR correlations reveal that, as predicted, the N–H of pyrrole and C12–H of 5a are close to each other, but their electrons are mutually repulsive due to the overlapping of spatial orientations, thus perhaps leading to a helical structure. Comparing the NMR spectra of 5a with those of 6a, it is found that the N–H of pyrrole is deprived by NaOH, forming a nitrogen anion. The C12–H of 6a exhibits enhanced acidity, demonstrating the formation of an intramolecular C12–H⋯N hydrogen bond, which is beneficial to stabilize the nitrogen anion of pyrrole. Undoubtedly, the formation of the intramolecular hydrogen bond can endow 6a with increased planarity. Thus, we speculate that, when helical 5a is transformed into planar 6a under alkaline conditions, the strong π–π stacking interactions among planar molecules would lead to the ACQ effect.

**Scheme 3:**
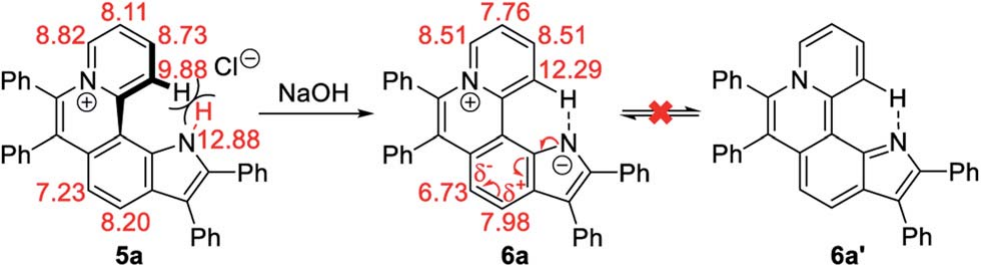
Conformation transformation from 5a to 6a and chemical shifts of their characteristic protons.

The ^1^H NMR titration experiments of 5a were performed (Fig. 2d). Upon addition of NaOH solution (2 M in water), the sharp singlet of pyrrole N–H at 12.88 ppm gradually weakens until it vanishes due to deprotonation under alkaline conditions. The doublet signal of C12–H at 9.88 ppm is remarkably downfield shifted with the increase of the concentration of NaOH, and finally shifted to 12.29 ppm when adding 2 equiv. of NaOH, which demonstrates a complete replacement of pyrrole N–H with the intramolecular C12–H⋯N hydrogen bond. The proton signals of C9–H, C10–H and C11–H at 8.82, 8.11 and 8.73 ppm are slightly upfield shifted to 8.51, 7.76 and 8.51 ppm, respectively, which indicates that the electron configuration of the pyridinium cation does not significantly change before and after adding NaOH. Compared with 5a, all proton signals except for those of C12–H on the pyrrolophenanthridizinium skeleton of 6a are upfield shifted owing to the electron-donating characteristic of the nitrogen anion. C5–H, located at the *para*-position of the nitrogen anion, shows an even larger upfield shift of 0.50 ppm. These results suggest the zwitterion structure of 6a rather than an electrically neutral resonance structure 6a′ (Scheme 3).

**Fig. 3:**
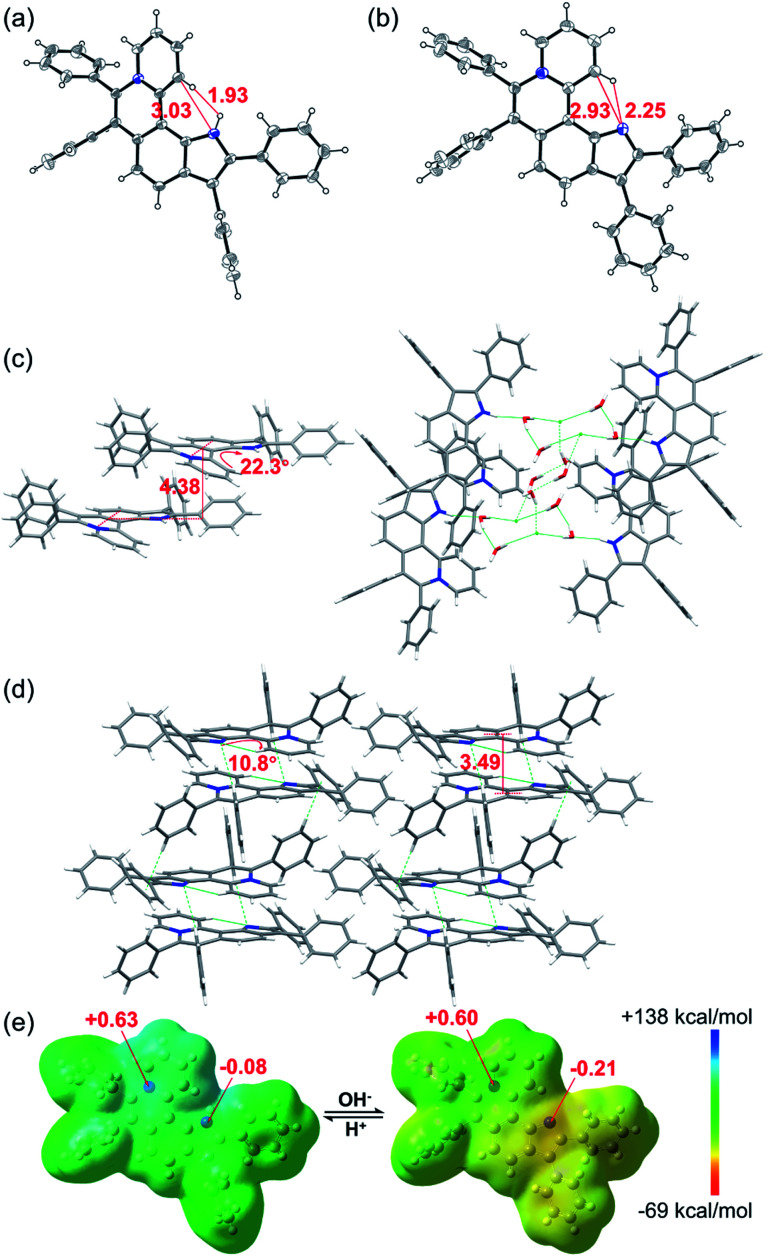
X-ray single-crystal structures of (a) 5a (CCDC 2034147, C⋯N 3.03 Å, H⋯H 1.93 Å) and (b) 6a (CCDC 2034148, C⋯N 2.93 Å, H⋯N 2.25 Å). Structures of 5a and 6a with thermal ellipsoids set at the 30% probability level and the anion of 5a is omitted for clarity. Stacked structures of (c) 5a (torsion angle = 22.3°, interlayer distance = 4.38 Å) and (d) 6a (torsion angle = 10.8°, interlayer distance = 3.49 Å). Hydrogen atoms are simulated using software. (e) Electrostatic potential maps and natural bond orbital (NBO) atomic charge distributions of 5a (left) and 6a (right) based on their crystal structures.

The single crystal X-ray diffraction (XRD) data indicate that the skeleton of 5a indeed shows a helical structure with a torsion angle of 22.3°, while 6a displays an approximately planar core skeleton with a relatively small torsion angle of 10.8° (Fig. 3a–d). The distance between N1–H and C12–H of 5a is 1.93 Å, suggesting the presence of the electrostatic repulsion between the two hydrogen atoms, which leads to the helical structure. The distance between N1 and C12 and the distance between N1 and C12–H of 6a are 2.93 Å and 2.25 Å, respectively, further confirming the presence of the intramolecular hydrogen bond (Fig. 3a and b). The helical structure endows 5a with loose J-aggregate packing with an interlayer distance of around 4.38 Å in the crystal structure, while planar 6a adopts tighter H-aggregate packing with an interlayer distance of around 3.49 Å (Fig. 3c and d). Multiple hydrogen-bond interaction networks among the anion, the pyrrole N–H, and water molecules make the loose packing motif of 5a stable (Fig. 3c). For 6a, intermolecular π⋯π, C–H⋯π and C–H⋯N interactions result in the tight molecular packing motif (Fig. 3d). It is well known that J-aggregates usually lead to enhanced emissive properties, while H-aggregates generally exhibit low fluorescence. Therefore, 5a exhibits a relatively strong emission and 6a displays apparent fluorescence quenching in both the solution and solid states (Fig. 1c and S14†).

To further confirm the zwitterion structure of 6a, electrostatic potential maps and natural bond orbital (NBO) atomic charge distributions of 5a and 6a were compared by density functional theory (DFT) computation based on their crystal structures, using Gaussian 09 at the B3LYP/6-311++G(2DF,2P) level.^11^ As shown in Fig. 3e, the positive charge mostly locates around the N8 atom in both 5a and 6a, on which the NBO charges are nearly equal, with 0.63 and 0.60, respectively, further confirming the pyridinium cation structure in 5a and 6a. In contrast, a significant change of the NBO charge occurs on the N1 atom from −0.08 of 5a to −0.21 of 6a, illustrating the transformation from N–H of pyrrole to the nitrogen anion. These results further verify the zwitterion structure of 6a.

As is well known, the p*K*_a_ values of acidic protons in organic molecules can be adjusted by the introduction of different substituents.^12^ To achieve the fine adjustment of p*K*_a_ values, a series of pyrrolo[3,2-*k*]phenanthridiziniums containing electron-donating or -withdrawing groups were synthesized *via* Rh-catalyzed cyclization and the subsequent anion exchange (Table 1, 5b–5k). This protocol was compatible with diphenylacetylenes with electron-donating substituents and bis(4-fluorophenyl)acetylene, but dialkylacetylene, diheteroarylacetylene, and diphenylacetylenes with electro-withdrawing groups did not deliver the corresponding aza[4]helicenes under standard conditions. In most of these sequential C–H activation/cyclization reactions, small amounts of phenanthridiziniums were detected as side products, and 7-(pyridin-2-yl)-1*H*-indoles were not detected. Considering that pyridine possesses stronger coordination ability than the carbonyl group of amides, it is speculated that the formation of the pyridinium ring was priority to that of pyrrole.

Subsequently, the photophysical properties of the resulting pyrrolo[3,2-*k*]phenanthridiziniums were studied (Table 1 and Section VII in the ESI†). Fluorescence titration experiments indicate that the p*K*_a_ values of 5b–5k vary from 4.18 to 8.76. Starting from the pH value of approximately 5.0, the emission intensities of 5b, 5d, 5f–5h, and 5j–5k sharply weaken with the increase of the pH values, which are roughly consistent with 5a. However, 5c exhibits a significant change of fluorescent intensity in the pH range from 2.76 to 5.23. The four bulky *tert*-butyl groups of 5c may have an impact on molecular planarity and the intermolecular packing motif, leading to a relatively lower p*K*_a_ value. Meanwhile, the fluorescence intensities of 5e and 5i start to dramatically weaken from the pH value of around 7.0.

**Fig. 4:**
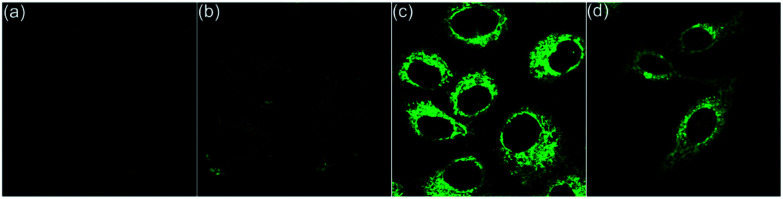
Fluorescence images of HepG2 cells. (a) Incubated with 5c for 20 min, and (b) followed by treatment with acetic acid for another 30 min. (c) Incubated with 5e for 20 min. (d) Incubated with 5i for 20 min.

Living cells possess a complicated intracellular pH environment from 4.5 to 8.0. Lysosomes, as the digestive system of exogenous substances, are the first defensive line of cells, which have a pH environment at 4.5–5.5 in virtue of diverse acidic hydrolases.^13^ Abnormal pH values in lysosomes are associated with some common diseases, such as Alzheimers and cancer.^14^ Considering the fluorescence on/off characteristic of the pH-controlled reversible conformation transformation of aza[4]helicenes (5) in the physiological pH range, the confocal fluorescence imaging experiments of HepG2 cells stained with 5 were performed. After incubation of HepG2 cells with 5a–5k for 20 min, bright intracellular fluorescence was captured by fluorescence microscopy for all compounds except 5c (Fig. 4 and 6). It is speculated that 5c shows a turn-off fluorescence emission in living cells owing to its relatively low pH transition interval from 2.76 to 5.23 (Fig. 4a). To verify this speculation, by incubation of HepG2 cells with 5c for 20 min, followed by treatment with acetic acid for another 30 min,^15^ green fluorescence was observed (Fig. 4b), suggesting that the intracellular pH environment quenches the fluorescence of 5c through the cleavage of its N1–H to trigger the transformation from helical to planar conformations. The addition of acetic acid results in a conformation inversion from the planar molecule to helicene, achieving turn on fluorescence. Without the addition of acetic acid, 5e and 5i also exhibit bright fluorescence throughout the whole cytoplasm due to their relatively larger p*K*_a_ values (8.76 and 7.74, respectively) (Fig. 4c and d).

**Fig. 5:**
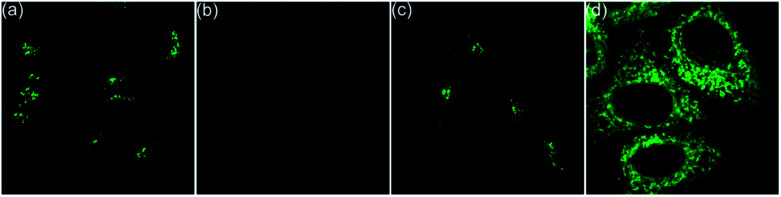
Fluorescence images of HepG2 cells. (a) Incubated with 5a for 1 h. (b) Incubated with 5a and hydroxychloroquine sulfate for 1 h. (c) Incubated with 5a for 30 min. (d) Incubated with 5a for 20 min, followed by treatment with acetic acid for 10 min.

**Fig. 6:**
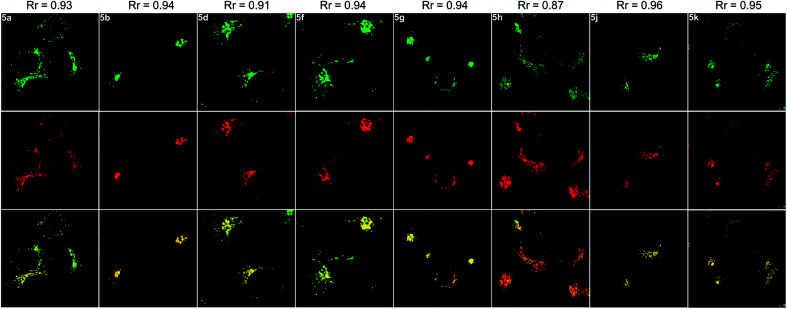
Fluorescence images of HepG2 cells incubated with 5 and LysoTracker™ Deep Red for 20 min. Top line: fluorescence images of HepG2 cells stained with 5a, 5b, 5d, 5f, 5g, 5h, 5j and 5k, respectively (*λ*_ex_ = 405 nm, *λ*_em_ = 470−570 nm). Middle line: fluorescence images of HepG2 cells cultured with LysoTracker™ Deep Red (*λ*_ex_ = 633 nm, *λ*_em_ = 640−740 nm). Bottom line: merged images. *R*_r_ = Pearson correlation coefficient.

Next, the co-staining experiments of HepG2 cells with 5a and commercially available LysoTracker™ Deep Red were carried out and the images from channel 1 (green luminescence from 5a) and channel 2 (red luminescence from LysoTracker™ Deep Red) overlapped very well with a Pearson's coefficient of 0.93, indicating that 5a specifically targeted the lysosome of living cells (Fig. 6, 5a). To validate the pH-controlled conformation transformation between the helical and planar molecules in living cells, hydroxychloroquine (HCQ) sulfate was employed as the lysosomal inhibitor to increase the alkalinity of lysosomes.^16^ After incubating HepG2 cells with 5a and HCQ for 1 h, bright fluorescence signals disappeared in HepG2 cells. In addition, when treating with alkaline phosphate buffer solution after incubating HepG2 cells with 5a, a turn-off fluorescence emission was also observed (Fig. S15†). These results demonstrate that the enhanced alkalinity removes the N1–H of 5a, and helical 5a is transformed into planar 6a, leading to turn-off fluorescence (Fig. 5a and b). After incubation of HepG2 cells with 5a for 20 min, followed by treatment with acetic acid for another 10 min,^15^ the luminescent region of 5a in living cells expanded to the whole cytoplasm, indicating that 5a indeed distributes in the whole cytoplasm and the relatively strong acidity of lysosome leads to its turn-on fluorescence (Fig. 5c and d). In other words, 5a can mark the acidic environment in living cells rather than specific organelles. Aza[4]helicenes 5b, 5d, 5f–5h, and 5j–5k also exhibit potential as lysosome-targeted biomarkers with Pearson's coefficients of 0.94, 0.91, 0.94, 0.94, 0.87, 0.96, and 0.95 (Fig. 6). The cytotoxicity of 5a, 5b, 5d, 5f–5h, and 5j–5k was evaluated by MTS assay. These compounds display low toxicity or non-toxicity to the cultured HepG2 cells (Fig. S16–S23†).

**Fig. 7:**
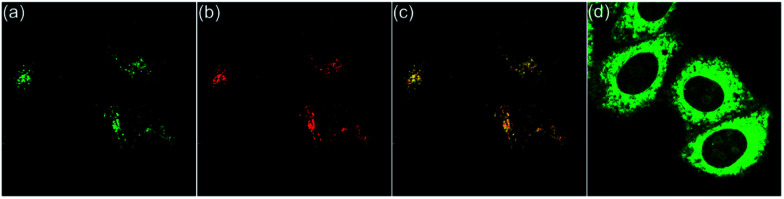
Fluorescence images of HepG2 cells incubated with 6a and LysoTracker™ Deep Red for 20 min. (a) Fluorescence images of HepG2 cells stained with 6a (*λ*_ex_ = 405 nm, *λ*_em_ = 470−570 nm). (b) Fluorescence images of HepG2 cells cultured with LysoTracker™ Deep Red (*λ*_ex_ = 633 nm, *λ*_em_ = 640−740 nm). (c) Merged images of (a) and (b) with a Pearson correlation coefficient of 0.95. (d) Incubated with 6a for 20 min, followed by treatment with acetic acid for 10 min.

To further confirm the reversible conformation transformation, the co-staining experiments with 6a and LysoTracker™ Deep Red were also conducted (Fig. 7a–c). Planar 6a did not show fluorescence emission in the culture medium outside the cells. However, green fluorescence in lysosomes was detected after incubating HepG2 cells with 6a for 20 min, which indicated that 6a successfully penetrated the cell membrane, and then underwent the protonation reaction of the pyrrole nitrogen anion owing to the acidic environment of lysosomes. Planar 6a was transformed into helical 5a through the protonation reaction, thus exhibiting bright green fluorescence (Fig. 7a). HepG2 cells were incubated with 6a for 20 min, washed twice with PBS solution, and then treated with acetic acid for 10 min. The green fluorescence was observed throughout the whole cytoplasm (Fig. 7d). These results demonstrate that the protonation reaction of the pyrrole nitrogen anion only takes place in lysosomes in the absence of acetic acid. Upon treating HepG2 cells with acetic acid, the protonation reaction can occur throughout the whole cytoplasm due to enhanced acidity.

## Conclusions

In summary, we have synthesized a novel class of aza[4]helicenes, pyrrolo[3,2-*k*]phenanthridiziniums, by two sequential rhodium-catalyzed C–H activation/cyclizations of 2-(pyridin-2-yl)anilines with alkynes. These aza[4]helicenes can be transformed into planar molecules through the cleavage of acidic pyrrole N–H, leading to turn-off fluorescence. The p*K*_a_ values of these acidic N–H bonds range from 4.18 to 8.76 depending on the electron-donating and -withdrawing groups on the conjugated skeleton. The conformation transformations between helical and planar molecules occur in the physiological pH range, thus affording an opportunity for pH-controlled intracellular selective fluorescence imaging. Pyrrolo[3,2-*k*]phenanthridiziniums can successfully penetrate the cell membrane and spread over the whole cytoplasm, but only exhibit bright fluorescence in lysosomes owing to the acidic environment of lysosomes. Outside lysosomes, the deprotonation of pyrrole leads to the transformation from aza[4]helicenes to planar conformations, thus showing turn-off fluorescence. These results indicate the potential of pyrrolo[3,2-*k*]phenanthridiziniums as lysosome-targeted biomarkers.

## Conflicts of interest

There are no conflicts to declare.

## Supplementary Material

SC-012-D0SC05844K-s001

SC-012-D0SC05844K-s002
