# Supplementary material for: Molecular engineering enabling reversible transformation between helical and planar conformations by cyclization of alkynes
Source: Chem Sci. 2020 Dec 8;12(7):2419–26. doi: 10.1039/d0sc05844k (PMC8179297; doi:10.1039/d0sc05844k)

Electronic Supplementary Material (ESI) for Chemical Science.
This journal is © The Royal Society of Chemistry 2020

Supplementary Information

Molecular engineering enabling reversible transformation between helical and planar conformations by cyclization of alkynes

Lipeng Yan, Weixin Ma, Jingbo Lan, Hu Cheng, Zhengyang Bin, Di Wu, and Jingsong You**

Key Laboratory of Green Chemistry and Technology of Ministry of Education, College of Chemistry, Sichuan University, 29 Wangjiang Road, Chengdu 610064, People's Republic of China

Table of Contents

I. General Remarks S3

II. Synthesis of *N*-acetyl-2-(pyridin-2-yl)aniline derivatives S3

III. Rh-catalyzed C–H activation/cyclization of 2-(pyridin-2-yl)aniline with diphenylacetylene S4

IV. Optimization of Rh-catalyzed dual C–H activation/cyclization of *N*-acetyl-2-(pyridin-2-yl)aniline with diphenylacetylene S5

V. General procedure for Rh-catalyzed dual C–H activation/cyclization and the subsequent anion exchange reaction S6

VI. Synthesis of **6a** S7

VII. Photophysical properties and calculation of pK_a values of **5** S7

VIII. Fluorescence spectra of **5a** and **6a** in solid state S15

IX. Cell experiments..S15

X. Preparation and characterization of the described compounds..........................S21

XI. References..S32

XII. Copies of ^{1}H, ^{13}C and ^{19}F NMR spectra..S33

I. General Remarks

Unless otherwise noted, all reagents were obtained from commercial suppliers and used without further purification. Diarylacetylene were synthesized by literature procedures.[1]

NMR spectra were obtained on an Agilent 400-MR DD2 or a Bruker AV II-400 MHz spectrometer. ^{1}H NMR (400 MHz) chemical shifts were measured relative to $CDCl_3$ or DMSO-d_6 as the internal reference ($CDCl_3$: δ = 7.26 ppm; DMSO-d_6: δ = 2.50 ppm). ^{13}C NMR (100 MHz) chemical shifts were measured relative to $CDCl_3$ or DMSO-d_6 as the internal reference ($CDCl_3$: δ = 77.16 ppm; DMSO-d_6: δ = 39.52 ppm). High-resolution mass spectra (HRMS) were obtained with a Shimadzu LCMS-IT-TOF (ESI) spectrometer. X-ray single crystal diffraction data were collected on an Agilent Gemini Plus single crystal diffractometer. Absorption and fluorescence spectra were obtained using a HITACHI U-2910 spectrometer and a Horiba Fluorolog-3 fluorescence spectrometer, respectively. Absolute quantum yields were collected with a calibrated integrating sphere system. Confocal fluorescence imaging measurements were conducted on a LSM 780 (Zeiss) confocal fluorescent microscope. Cytotoxicity experiments were carried out by CellTiler 96® AQueous One Solution Cell Proliferation Assay.

II. Synthesis of *N*-acetyl-2-(pyridin-2-yl)aniline derivatives

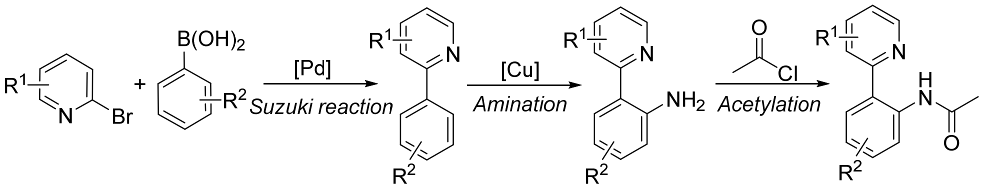

Suzuki reaction: A mixture of 2-bromopyridine derivative (5 mmol), arylboronic acid (7 mmol), $Pd(PPh_3)_4$ (0.25 mmol), Na_2CO_3 (25 mmol), ethanol (6 mL), toluene (30 mL) and water (30 mL) was stirred at 100 °C overnight under N_2. Then, the mixture was poured into brine and extracted with ethyl acetate three times. The organic phase was dried over Na_2SO_4, filtered and concentrated under reduced pressure. The

residue was purified by column chromatography on silica gel to give the 2-arylpyridine derivative.

Amination:[2] A mixture of 2-arylpyridine derivative (2 mmol), trimethylsilyl azide (4 mmol), copper trifluoroacetate (2 mmol), trifluoroacetic acid (2 mmol) and anhydrous 1,2-dichlorobenzene (20 mL) was stirred at 115 °C for 24 h under N_2. After the mixture was cooled to room temperature, 1 mL of ammonia was added and stirred for another 10 min. Then, the mixture was diluted in ethyl acetate (50 mL), and washed with saline three times. The organic phase was dried over Na_2SO_4, filtered and concentrated under reduced pressure. The residue was purified by column chromatography on silica gel to give the 2-(pyridin-2-yl)aniline derivative.

Acetylation: Acetyl chloride (1.5 mmol) was added to a solution of 2-(pyridin-2-yl)aniline derivative (1 mmol), K_2CO_3 (3 mmol), ethyl acetate (10 mL) and water (5 mL). The resulting mixture was stirred at room temperature for 2 h. Then, the mixture was diluted in ethyl acetate (50 mL), and washed with saline three times. The organic phase was dried over Na_2SO_4, filtered, and concentrated under reduced pressure. The residue was purified by column chromatography on silica gel to give the *N*-acetyl-2-(pyridin-2-yl)aniline derivative.

III. Rh-catalyzed C–H activation/cyclization of 2-(pyridin-2-yl)aniline with diphenylacetylene

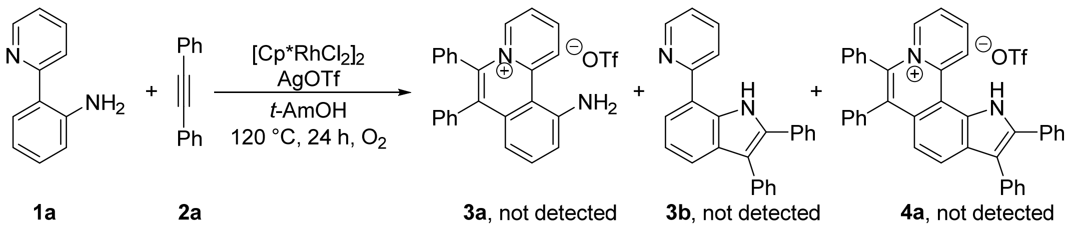

A 25 mL Schlenk sealed tube with a magnetic stir bar was charged with 2-(pyridin-2-yl)aniline **1a** (17.0 mg, 0.1 mmol), diphenylacetylene **2a** (35.6 mg, 0.2 mmol), $[Cp^*RhCl_2]_2$ (3.1 mg, 5 mol%), AgOTf (30.8 mg, 0.12 mmol) and 2-methylbutan-2-ol (*t*-AmOH, 1.5 mL) under O_2. The resulting mixture was stirred at

120 °C for 24 h and then diluted with 10 mL of dichloromethane. The results of detection by thin-layer chromatography (TLC) indicated that this reaction delivered no annulated product **3a**, **3b** and **4a**.

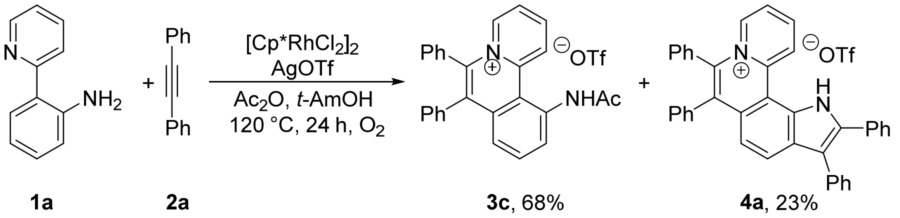

A 25 mL Schlenk sealed tube with a magnetic stir bar was charged with 2-(pyridin-2-yl)aniline **1a** (17.0 mg, 0.1 mmol), diphenylacetylene **2a** (35.6 mg, 0.2 mmol), $[Cp*RhCl_2]_2$ (3.1 mg, 5 mol%), AgOTf (30.8 mg, 0.12 mmol), acetic anhydride (47 μL, 0.5 mmol) and 2-methylbutan-2-ol (*t*-AmOH, 1.5 mL) under O_2. The resulting mixture was stirred at 120 °C for 24 h and then diluted with 10 mL of dichloromethane. The mixture was filtered through a celite pad and washed with 20 mL of dichloromethane. Then the filtrate was concentrated under reduced pressure. The residue was purified by column chromatography on silica gel to give **3c** as a grayish-white solid (dichloromethane/methanol = 10/1, v/v, 36.7 mg, 68% yield) and **4a** as a yellow solid (dichloromethane/methanol = 30/1, v/v, 15.5 mg, 23% yield).

IV. Optimization of Rh-catalyzed dual C–H activation/cyclization of *N*-acetyl-2-(pyridin-2-yl)aniline with diphenylacetylene

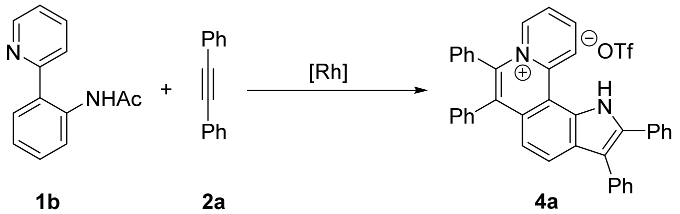

A 25 mL Schlenk sealed tube with a magnetic stir bar was charged with *N*-acetyl-2-(pyridin-2-yl)aniline **1b** (21.2 mg, 0.1 mmol), diphenylacetylene **2a** (35.6 mg, 0.2 mmol), $[Cp*RhCl_2]_2$, silver salt, oxidant, additive and 2-methylbutan-2-ol (*t*-AmOH) under N_2. The resulting mixture was stirred at 120-140 °C for 24 h and then

diluted with 10 mL of dichloromethane. The mixture was filtered through a celite pad and washed with 20 mL of dichloromethane. Then the filtrate was concentrated under reduced pressure. The residue was purified by column chromatography on silica gel (dichloromethane/methanol = 30/1, v/v) to give the isolated yield of **4a**.

Table S1. Optimization of reaction conditions.[a]

Entry	[Rh]	[Ag]	Oxidant	Additive	Solvent	Yield (%)[b]
1[c]	$[Cp^*RhCl_2]_2$	AgOTf	Oxygen (1 atm)	—	*t*-AmOH	27
2	$[Cp^*RhCl_2]_2$	AgOTf	Oxygen (1 atm)	TfOH	*t*-AmOH	N.D.
3	$[Cp^*RhCl_2]_2$	AgOTf	$Cu(OAc)_2·H_2O$	TfOH	*t*-AmOH	39
4	$[Cp^*RhCl_2]_2$	$AgSbF_6$	$Cu(OAc)_2·H_2O$	TfOH	*t*-AmOH	46
5[d]	$[Cp^*RhCl_2]_2$	$AgSbF_6$	$Cu(OAc)_2·H_2O$	TfOH	*t*-AmOH	57
6[e]	$[Cp^*RhCl_2]_2$	$AgSbF_6$	$Cu(OAc)_2·H_2O$	TfOH	*t*-AmOH	60
7[f]	$[Cp^*RhCl_2]_2$	$AgSbF_6$	$Cu(OAc)_2·H_2O$	TfOH	*t*-AmOH	69
8[g]	$[Cp^*RhCl_2]_2$	$AgSbF_6$	$Cu(OAc)_2·H_2O$	TfOH	*t*-AmOH	82
9[h]	$[Cp^*Co(CO)I_2]$	$AgSbF_6$	$Cu(OAc)_2·H_2O$	TfOH	*t*-AmOH	N.D.

[a]Reactions were carried out by using **1b** (0.1 mmol), **2a** (0.2 mmol), $[Cp^*RhCl_2]_2$ (5 mol%), [Ag] (20 mol%), oxidant (0.2 mmol) and additive (0.1 mmol) in 2-methylbutan-2-ol (*t*-AmOH, 1.5 mL) at 120 °C for 24 h under an N_2 atmosphere. [b]Isolated yields. [c]AgOTf (0.12 mmol). [d]$Cu(OAc)_2·H_2O$ (0.3 mmol). [e]$Cu(OAc)_2·H_2O$ (0.3 mmol), TfOH (0.15 mmol). [f]**2a** (0.3 mmol), $Cu(OAc)_2·H_2O$ (0.3 mmol), TfOH (0.15 mmol). [g]**2a** (0.3 mmol), $Cu(OAc)_2·H_2O$ (0.3 mmol), TfOH (0.15 mmol), 140 °C. [h]**2a** (0.3 mmol), $[Cp^*Co(CO)I_2]$ (10 mol%), $Cu(OAc)_2·H_2O$ (0.3 mmol), TfOH (0.15 mmol), 140 °C. AgOTf = silver trifluoromethanesulfonate. TfOH = trifluoromethanesulfonic acid. N.D. = not detected.

V. General procedure for Rh-catalyzed dual C–H activation/cyclization and the subsequent anion exchange reaction

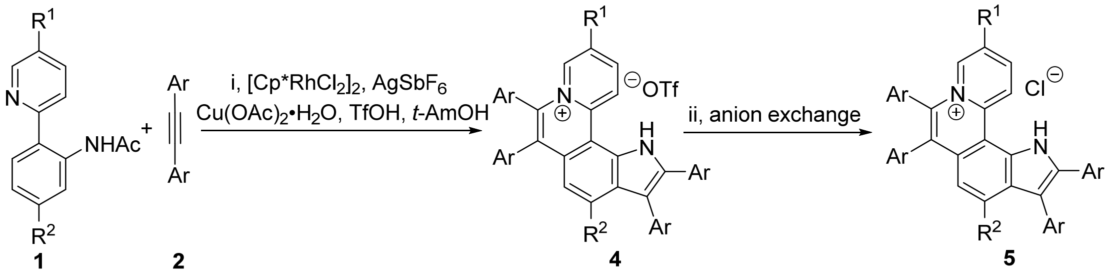

A 25 mL Schlenk sealed tube with a magnetic stir bar was charged with *N*-acetyl-2-(pyridin-2-yl)aniline derivative **1** (0.1 mmol), diarylacetylene **2** (0.3 mmol),

$[Cp^*RhCl_2]_2$ (5 mol%), $AgSbF_6$ (20 mol%), $Cu(OAc)_2{\cdot}H_2O$ (0.3 mmol), trifluoromethanesulfonic acid (0.15 mmol) and 2-methylbutan-2-ol (*t*-AmOH) under an N_2 atmosphere. The resulting mixture was stirred at 140 °C for 24 h and then diluted with 10 mL of dichloromethane. The mixture was filtered through a celite pad and washed with 20 mL of dichloromethane. Then the filtrate was concentrated under reduced pressure. The residue was purified by column chromatography on silica gel (dichloromethane/methanol = 30/1, v/v) to give the trifluoromethanesulfonate product **4**.

Compound **4** was dissolved in ethanol and water (3:1, v/v), and eluted *via* column chromatography on chlorine-ion exchange resin. The resulting solution was concentrated under reduced pressure. The residue was dissolved in dichloromethane, washed with diluted hydrochloric acid, dried over Na_2SO_4, filtered. The filtrate was concentrated under reduced pressure, and the residue was purified by column chromatography on silica gel (dichloromethane/methanol = 20/1, v/v) to give the desired product **5**.

VI. Synthesis of 6a

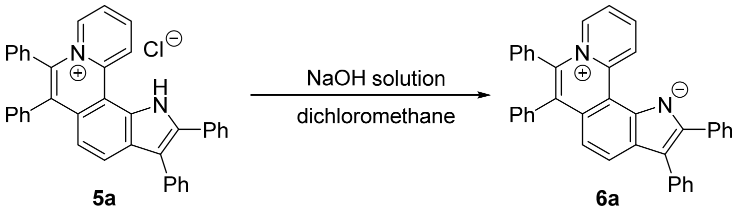

2 M of NaOH solution (1 mL) was added to the dichloromethane solution (5 mL) of compound **5a** (50.0 mg, 89 μmol). After stirred at room temperature for 5 min, the resulting mixture was stratified and separated. The organic phase was dried over Na_2SO_4, and filtered. Dichloromethane was removed under reduced pressure to give product **6a** as a dark red solid in nearly 100% yield (46.4 mg).

VII. Photophysical properties and calculation of pK_a values of 5

7.1 Absorption and emission spectra of 5 in dichloromethane

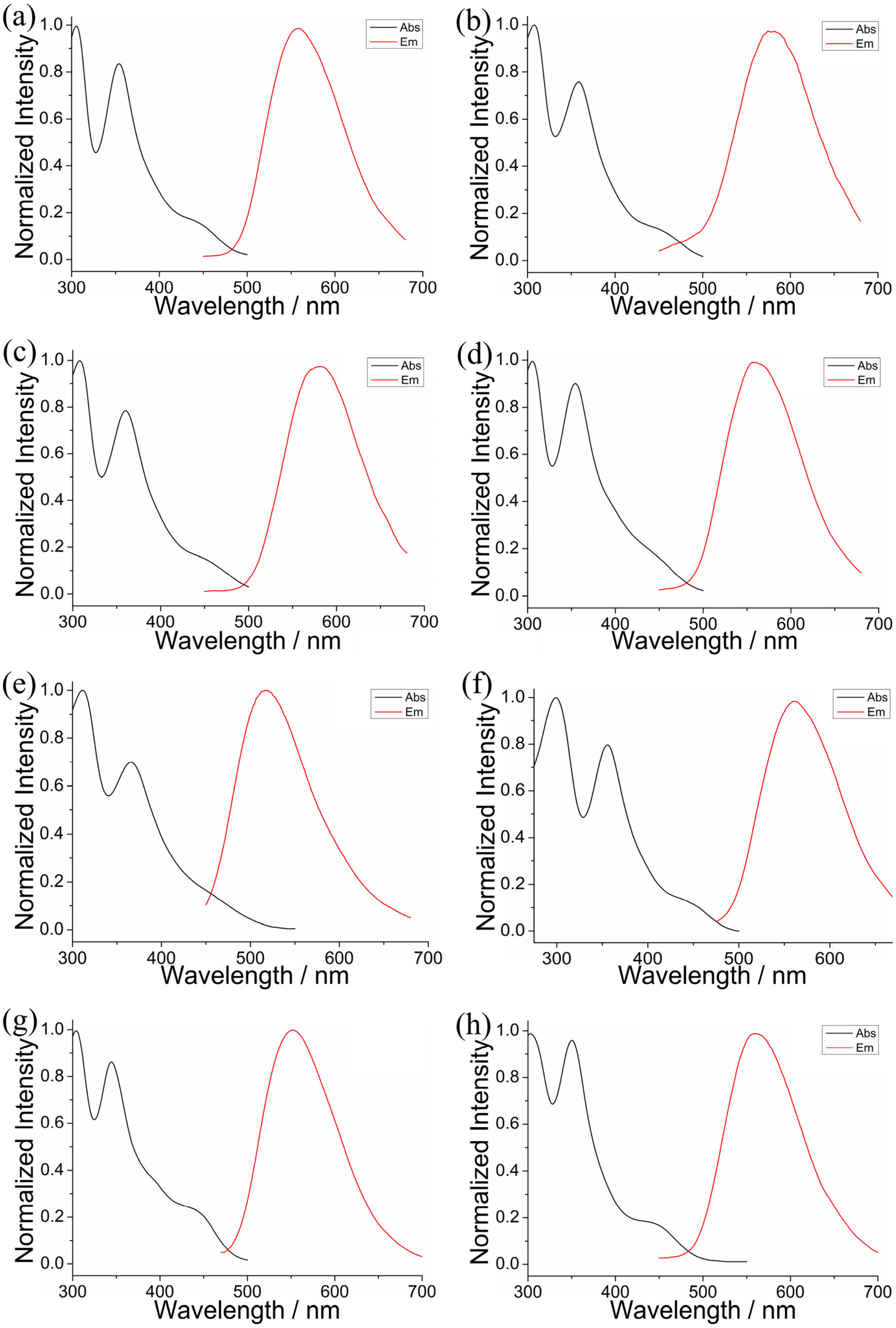
(a)
(b)
(c)
(d)
(e)
(f)
(g)
(h)
Normalized Intensity
Wavelength / nm
Abs
Em
0.0
0.2
0.4
0.6
0.8
1.0
300
400
500
600
700

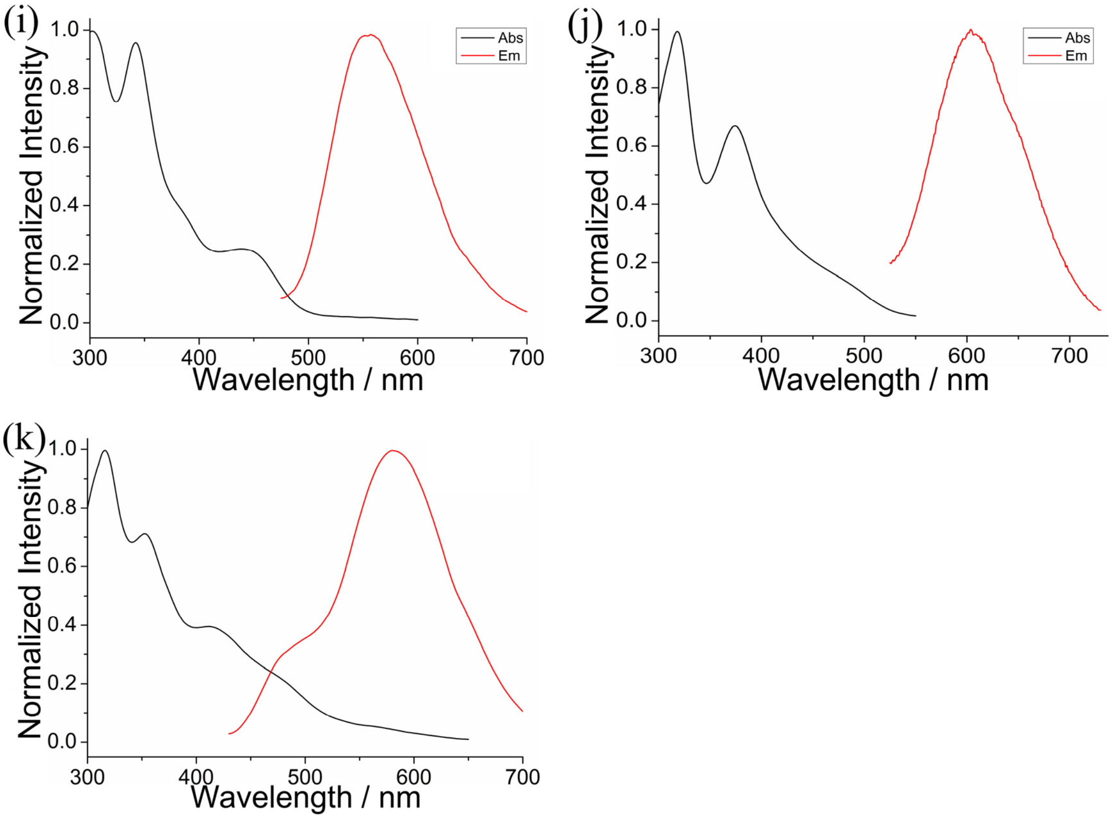

Fig. S1 Absorption and emission spectra of **5** in dichloromethane (1×10^{-5} M). (a) **5a**, 9%; (b) **5b**, 4%; (c) **5c**, 4%; (d) **5d**, 6%; (e) **5e**, 3%; (f) **5f**, 9%; (g) **5g**, 27%; (h) **5h**, 8%; (i) **5i**, 16%; (j) **5j**, 1%; (k) **5k**, 2%. The absolute quantum yields of **5a**-**5k** in CH_2Cl_2 (1×10^{-5} M), which are collected with an integrating sphere system, are shown after the identifiers of corresponding compounds.

7.2 Fluorescence spectra of 5 in phosphate-buffered saline with different pH values

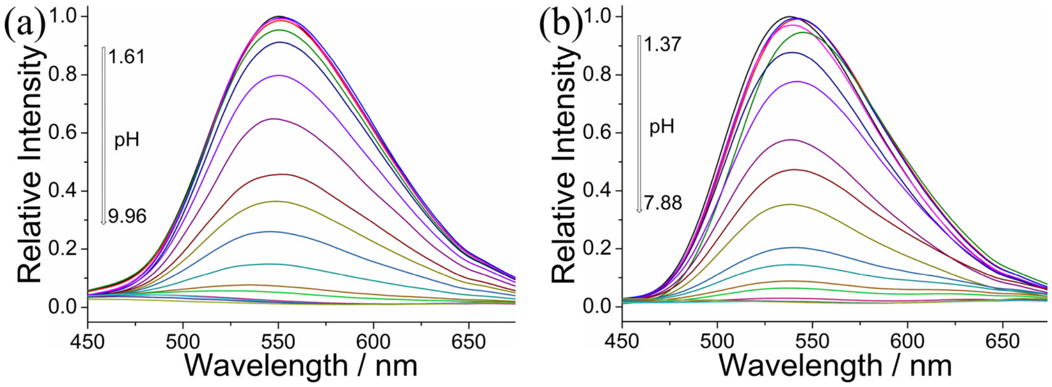

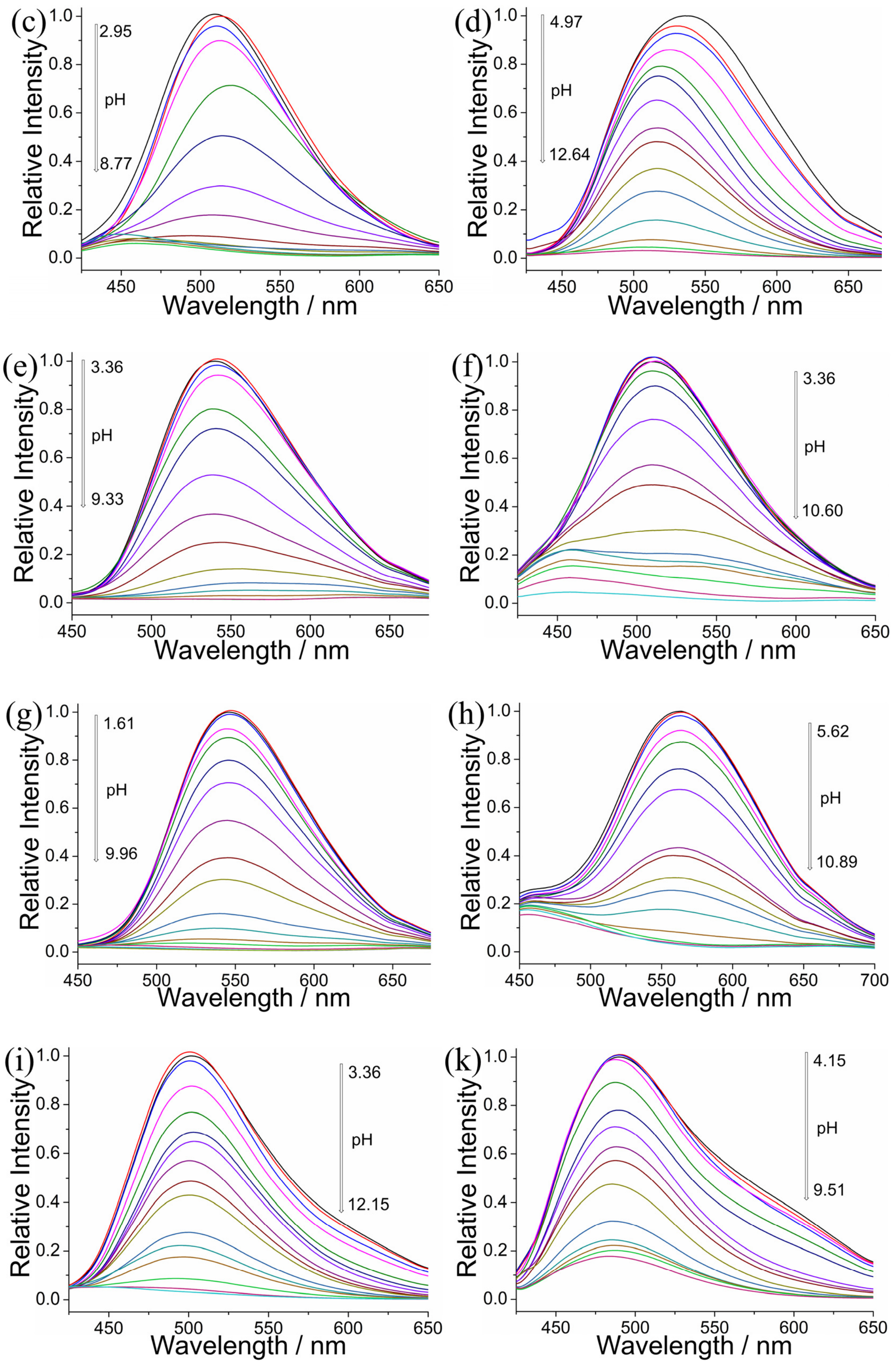

Fig. S2 Fluorescence spectra of **5** in phosphate-buffered saline with different pH values (2×10^{-5} M). (a) **5b**, DMSO/H_2O = 1:9, v/v; (b) **5c**, DMSO/H_2O = 1:9, v/v; (c) **5d**, DMSO/H_2O = 1:9, v/v; (d) **5e**, DMSO/H_2O = 1:9, v/v; (e) **5f**, DMSO/H_2O = 1:9, v/v; (f) **5g**, DMSO/H_2O = 1:9, v/v; (g) **5h**, DMSO/H_2O = 1:9, v/v; (h) **5i**, DMSO/H_2O = 1:1, v/v; (i) **5j**, DMSO/H_2O = 1:9, v/v; (j) **5k**, DMSO/H_2O

= 1:9, v/v.

7.3 Calculation of pK_a values of 5

The analysis on fluorescence intensities of compound **5** with different pH values by using Henderson-Hasselbach equation

$$\mathrm{LOG}\ [(I_{max} - I)/(I - I_{min})] = \mathrm{p}K_a - \mathrm{pH}$$

where I represents the fluorescence intensity at a certain wavelength, I_{max} and I_{min} are corresponding maximum and minimum limiting values of I, respectively.[3]

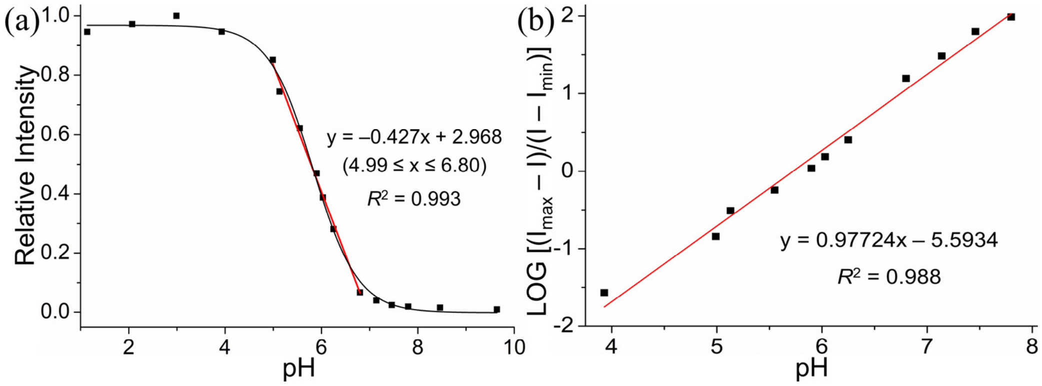

Fig. S3 (a) Relative fluorescence intensities of **5a** at 538 nm in phosphate-buffered saline with different pH values (2×10^{-5} M, pH = 1.14-9.64, DMSO/H_2O = 1:9, v/v). Inset: The linear relationship between relative fluorescence intensity of **5a** at 538 nm and pH value ranging from 4.99 to 6.80. (b) Linear relationship between LOG $[(I_{max}-I)/(I-I_{min})]$ and pH values. Based on the pH titration results, the pK_a value of **5a** was calculated to be 5.72 in DMSO–water buffer system.

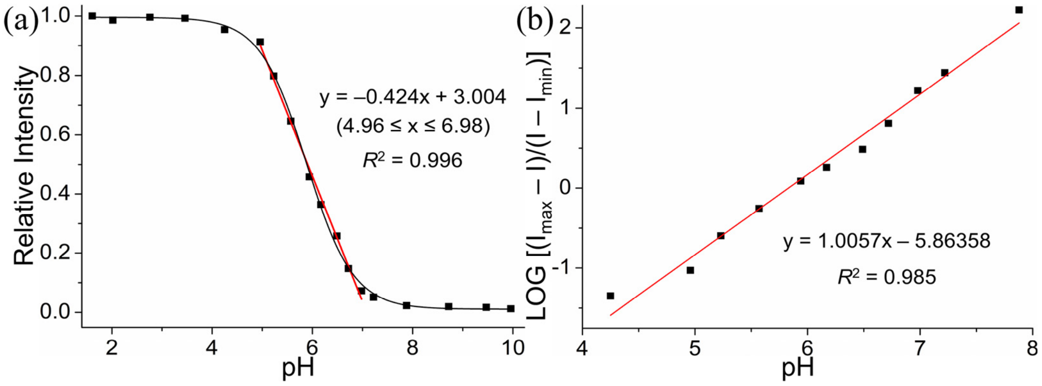

Fig. S4 (a) Relative fluorescence intensities of **5b** at 551 nm in phosphate-buffered saline with different pH values (2×10^{-5} M, pH = 1.61-9.96, DMSO/H_2O = 1:9, v/v). Inset: The linear relationship between relative fluorescence intensity of **5b** at 551 nm and pH value ranging from 4.96 to 6.98. (b) Linear relationship between LOG $[(I_{max}-I)/(I-I_{min})]$ and pH values. Based on the pH titration results, the pK_a value of **5b** was calculated to be 5.83 in DMSO–water buffer system.

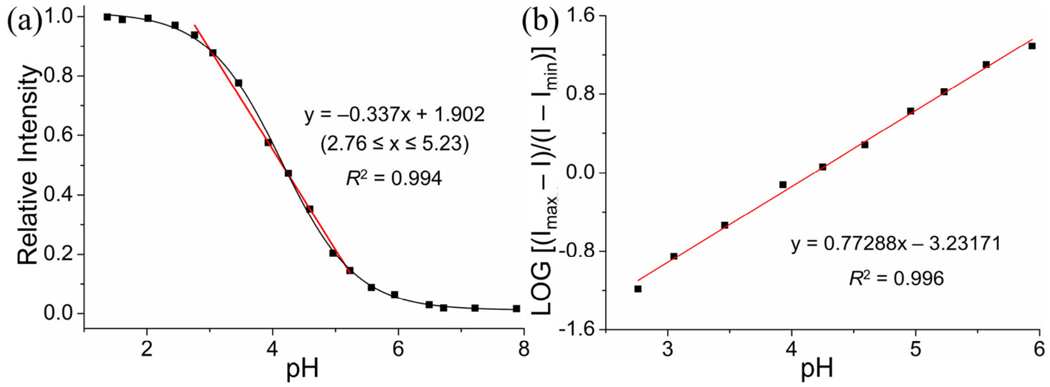

Fig. S5 (a) Relative fluorescence intensities of **5c** at 540 nm in phosphate-buffered saline with different pH values (2×10^{-5} M, pH = 1.37-7.88, DMSO/H_2O = 1:9, v/v). Inset: The linear relationship between relative fluorescence intensity of **5c** at 540 nm and pH value ranging from 2.76 to 5.23. (b) Linear relationship between LOG [(I_{max}–I)/(I–I_{min})] and pH values. Based on the pH titration results, the pK_a value of **5c** was calculated to be 4.18 in DMSO–water buffer system.

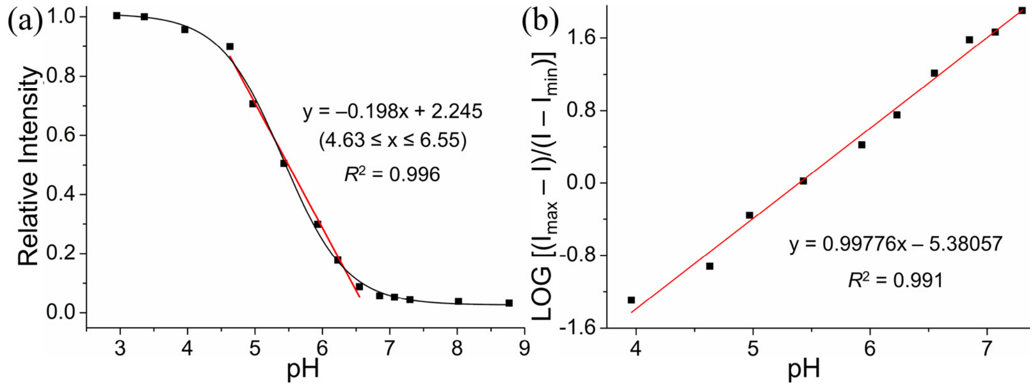

Fig. S6 (a) Relative fluorescence intensities of **5d** at 513 nm in phosphate-buffered saline with different pH values (2×10^{-5} M, pH = 2.95-8.77, DMSO/H_2O = 1:9, v/v). Inset: The linear relationship between relative fluorescence intensity of **5d** at 513 nm and pH value ranging from 4.63 to 6.55. (b) Linear relationship between LOG [(I_{max}–I)/(I–I_{min})] and pH values. Based on the pH titration results, the pK_a value of **5d** was calculated to be 5.39 in DMSO–water buffer system.

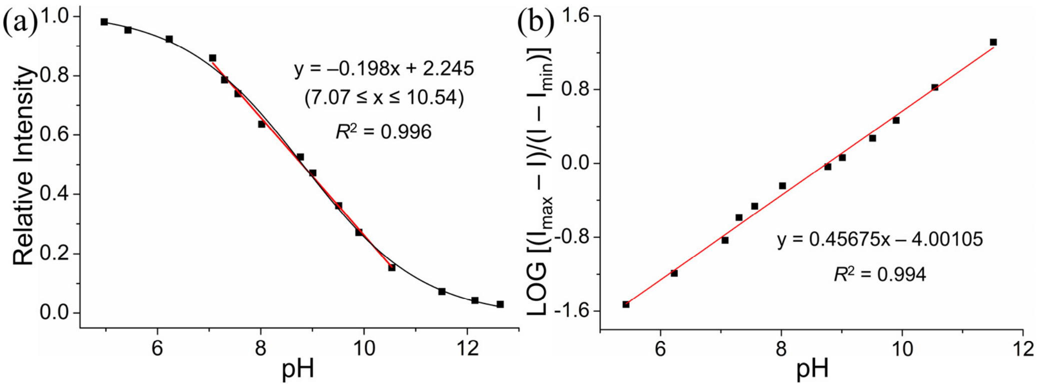

Fig. S7 (a) Relative fluorescence intensities of **5e** at 525 nm in phosphate-buffered saline with different pH values (2×10^{-5} M, pH = 4.97-12.64, DMSO/H_2O = 1:9, v/v). Inset: The linear

relationship between relative fluorescence intensity of **5e** at 525 nm and pH value ranging from 7.07 to 10.54. (b) Linear relationship between LOG [(I_{max}–I)/(I–I_{min})] and pH values. Based on the pH titration results, the pK_a value of **5e** was calculated to be 8.76 in DMSO–water buffer system.

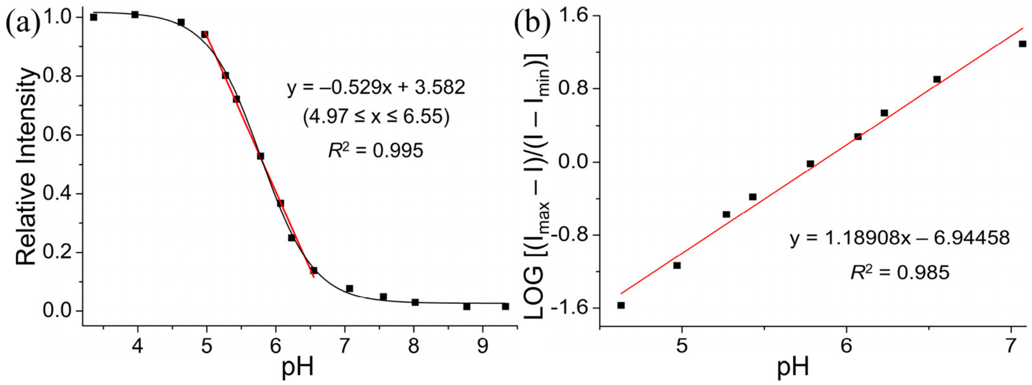

Fig. S8 (a) Relative fluorescence intensities of **5f** at 540 nm in phosphate-buffered saline with different pH values (2 × 10^{-5} M, pH = 3.36-9.33, DMSO/H_2O = 1:9, v/v). Inset: The linear relationship between relative fluorescence intensity of **5f** at 540 nm and pH value ranging from 4.97 to 6.55. (b) Linear relationship between LOG [(I_{max}–I)/(I–I_{min})] and pH values. Based on the pH titration results, the pK_a value of **5f** was calculated to be 5.84 in DMSO–water buffer system.

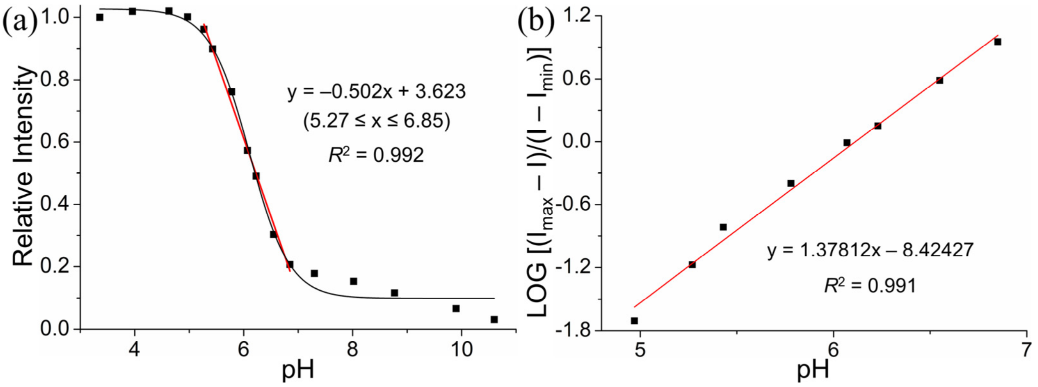

Fig. S9 (a) Relative fluorescence intensities of **5g** at 510 nm in phosphate-buffered saline with different pH values (2 × 10^{-5} M, pH = 3.36-10.60, DMSO/H_2O = 1:9, v/v). Inset: The linear relationship between relative fluorescence intensity of **5g** at 510 nm and pH value ranging from 5.27 to 6.85. (b) Linear relationship between LOG [(I_{max}–I)/(I–I_{min})] and pH values. Based on the pH titration results, the pK_a value of **5g** was calculated to be 6.11 in DMSO–water buffer system.

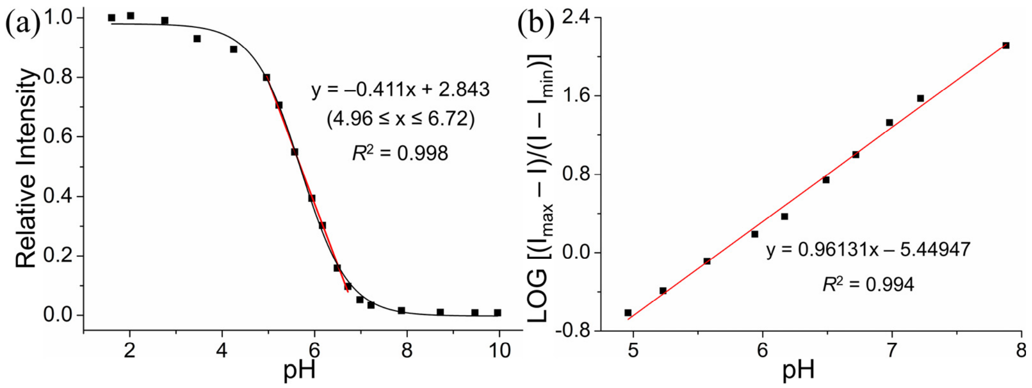

Fig. S10 (a) Relative fluorescence intensities of **5h** at 546 nm in phosphate-buffered saline with different pH values (2×10^{-5} M, pH = 1.61-9.96, DMSO/H_2O = 1:9, v/v). Inset: The linear relationship between relative fluorescence intensity of **5h** at 546 nm and pH value ranging from 4.96 to 6.72. (b) Linear relationship between LOG [$(I_{max}–I)/(I–I_{min})$] and pH values. Based on the pH titration results, the pK_a value of **5h** was calculated to be 5.67 in DMSO–water buffer system.

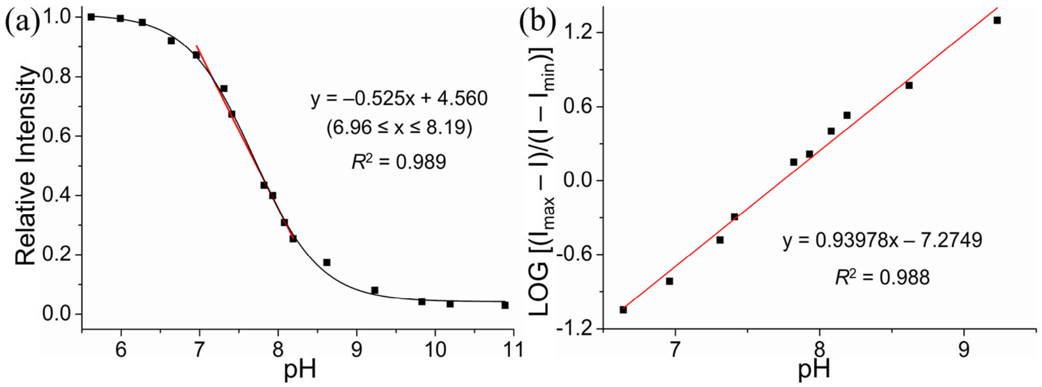

Fig. S11 (a) Relative fluorescence intensities of **5i** at 563 nm in phosphate-buffered saline with different pH values (2×10^{-5} M, pH = 5.62-10.89, DMSO/H_2O = 1:1, v/v). Inset: The linear relationship between relative fluorescence intensity of **5i** at 563 nm and pH value ranging from 6.96 to 8.19. (b) Linear relationship between LOG [$(I_{max}–I)/(I–I_{min})$] and pH values. Based on the pH titration results, the pK_a value of **5i** was calculated to be 7.74 in DMSO–water buffer system.

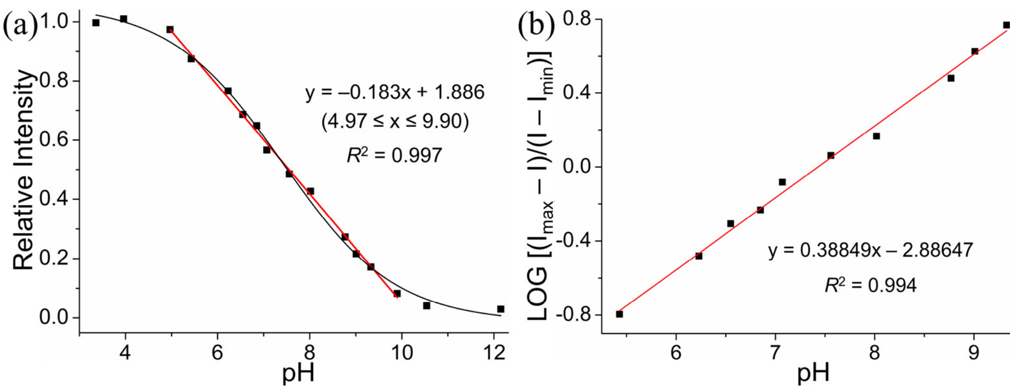

Fig. S12 (a) Relative fluorescence intensities of **5j** at 505 nm in phosphate-buffered saline with different pH values (2×10^{-5} M, pH = 3.36-12.15, DMSO/H_2O = 1:9, v/v). Inset: The linear

relationship between relative fluorescence intensity of **5j** at 505 nm and pH value ranging from 4.97 to 9.90. (b) Linear relationship between LOG [$(I_{max}-I)/(I-I_{min})$] and pH values. Based on the pH titration results, the pK_a value of **5j** was calculated to be 7.43 in DMSO–water buffer system.

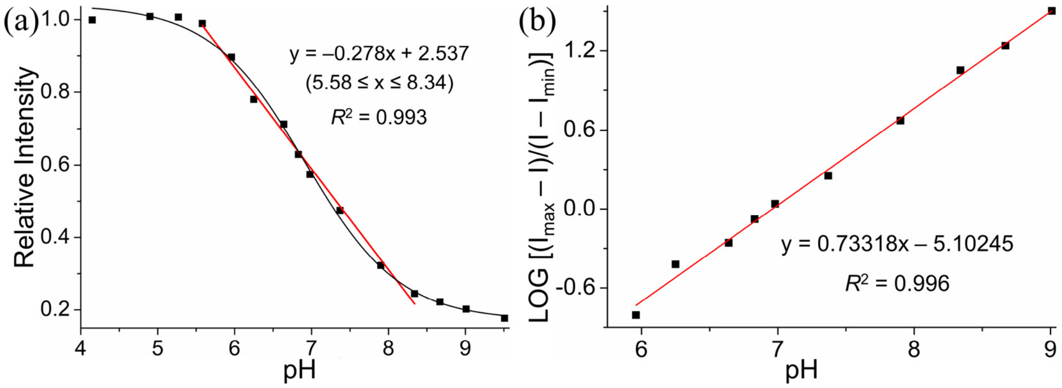

Fig. S13 (a) Relative fluorescence intensities of **5k** at 488 nm in phosphate-buffered saline with different pH values (2×10^{-5} M, pH = 4.15-9.51, DMSO/H_2O = 1:9, v/v). Inset: The linear relationship between relative fluorescence intensity of **5k** at 488 nm and pH value ranging from 5.58 to 8.34. (b) Linear relationship between LOG [$(I_{max}-I)/(I-I_{min})$] and pH values. Based on the pH titration results, the pK_a value of **5k** was calculated to be 6.96 in DMSO–water buffer system.

VIII. Fluorescence spectra of 5a and 6a in solid state

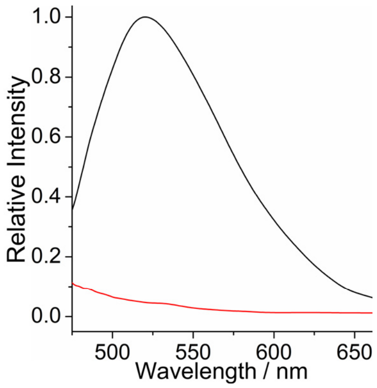

Fig. S14 Fluorescence spectra of **5a** (black) and **6a** (red) in solid state.

IX. Cell experiments

9.1 Cell culture

HepG2 cells were incubated in Dublecco's Modified Eagle's Medium (DMEM) supplemented with 10% (v/v) fetal bovine serum (FBS), 100 mg/mL of streptomycin and 100 units/mL of penicillin at 37 °C in humidified atmosphere containing 5% CO_2.

9.2 Confocal fluorescent imaging experiments

Confocal fluorescent imaging experiments incubated with 5: HepG2 cells were incubated with **5** (2 μM) in DMEM containing 1‰ DMSO for 20 min at 37 °C. Then, HepG2 cells were washed twice with phosphate buffered saline (PBS solution, 1 mL × 2), and covered with 1 mL of DMEM before imaging. The cells were observed with a Zeiss LSM 780 confocal fluorescent microscope.

Confocal fluorescent imaging experiments incubated with 5c and treated with acetic acid: HepG2 cells were incubated with **5c** (2 μM) in DMEM containing 1‰ DMSO for 20 min at 37 °C. Then, HepG2 cells were washed twice with phosphate buffered saline (PBS solution, 1 mL × 2), covered with 1 mL of DMEM, and treated with acetic acid (20 μL) for 30 min before imaging. The cells were observed with a Zeiss LSM 780 confocal fluorescent microscope.

Co-staining experiments: HepG2 cells were incubated with compound **5** or **6a** (2 μM) in DMEM containing 1‰ DMSO for 20 min at 37 °C. After washed twice with phosphate buffered saline (PBS solution, 1 mL × 2), LysoTracker^{TM} Deep Red (50 nM) was added to incubate for another 20 min. Finally, HepG2 cells were washed twice with PBS solution (1 mL × 2) and covered with 1 mL of DMEM before imaging. The cells were observed with a Zeiss LSM 780 confocal fluorescent microscope. In order to show effective contrasts, appropriate colors were chose to the labeled images of organelles, thus giving pseudo colors.

Confocal fluorescent imaging experiments incubated with 5a and treated with hydroxychloroquine sulfate: Two groups of comparative experiments were carried out simultaneously. HepG2 cells were incubated with **5a** (2 μM) only, or incubated with **5a** (2 μM) and hydroxychloroquine sulfate (150 μM), in DMEM containing 1‰ DMSO for 60 min at 37 °C. Then, HepG2 cells were washed twice with phosphate buffered saline (PBS solution, 1 mL × 2), and covered with 1 mL of DMEM before imaging. The cells were observed with a Zeiss LSM 780 confocal fluorescent microscope.

Confocal fluorescent imaging experiments incubated with 5a and treated with

acetic acid: Two groups of comparative experiments were carried out simultaneously. On the one hand, HepG2 cells were incubated with **5a** (2 μM) in DMEM containing 1‰ DMSO for 30 min at 37 °C. Then, HepG2 cells were washed twice with phosphate buffered saline (PBS solution, 1 mL × 2), and covered with 1 mL of DMEM before imaging. On the other hand, HepG2 cells were incubated with **5a** (2 μM) in DMEM containing 1‰ DMSO for 20 min at 37 °C. Then, HepG2 cells were washed twice with phosphate buffered saline (PBS solution, 1 mL × 2), covered with 1 mL of DMEM, and treated with acetic acid (10 μL) for 10 min before imaging. The cells were observed with a Zeiss LSM 780 confocal fluorescent microscope.

Confocal fluorescent imaging experiments incubated with 6a and treated with acetic acid: HepG2 cells were incubated with **6a** (2 μM) in DMEM containing 1‰ DMSO for 20 min at 37 °C. Then, HepG2 cells were washed twice with phosphate buffered saline (PBS solution, 1 mL × 2), covered with 1 mL of DMEM, and treated with acetic acid (10 μL) for 10 min before imaging. The cells were observed with a Zeiss LSM 780 confocal fluorescent microscope.

Confocal fluorescent imaging experiments incubated with 5a, and followed by the treatment with alkaline buffer solution: Two groups of comparative experiments were carried out simultaneously. On the one hand, HepG2 cells were incubated with **5a** (2 μM) in DMEM containing 1‰ DMSO for 20 min at 37 °C, then washed twice with phosphate buffered saline (PBS solution, 1 mL × 2), and covered with 1 mL of PBS solution before imaging. On the other hand, HepG2 cells were incubated with **5a** (2 μM) in DMEM containing 1‰ DMSO for 20 min at 37 °C, then washed twice with phosphate buffered saline (PBS solution, 1 mL × 2), covered with the mixture solution containing 1 mL of PBS solution and 2 μL of NaOH solution (1 M) for 30 min before imaging. The cells were observed with a Zeiss LSM 780 confocal fluorescent microscope.

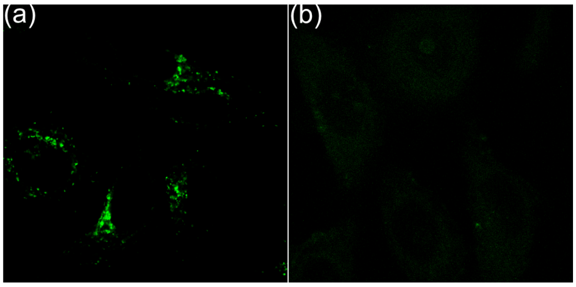

Fig. S15 Fluorescence images of HepG2 cells. (a) Incubated with **5a** for 20 min. (b) Incubated with **5a** for 20 min, and followed by the treatment with alkaline phosphate buffer solution for 30 min.

9.3 Cytotoxicity assay

The cytotoxicity experiments were investigated by CellTiler 96® AQueous One Solution Cell Proliferation Assay. HepG2 cells were seeded in 96-well culture plates and incubated for 24 h in stationary cultures. Then, the culture medium was replaced with a fresh complete medium containing compound **5** at concentrations of 0, 0.625, 1.25, 2.5, 5, 8, and 10 μM, respectively (0 μM for the control experiment). After another 24 h of incubation, 20 μL of CellTiler 96® AQueous One Solution was added to each well, and incubated for further one hour. Afterwards, the absorbance of each well was recorded on the ELISA plate reader (model 680, BioRad) at a wavelength of 490 nm. The cell viability was calculated by the following formula:

(Mean optical density in the treated well / Mean optical density in the control well) × 100%

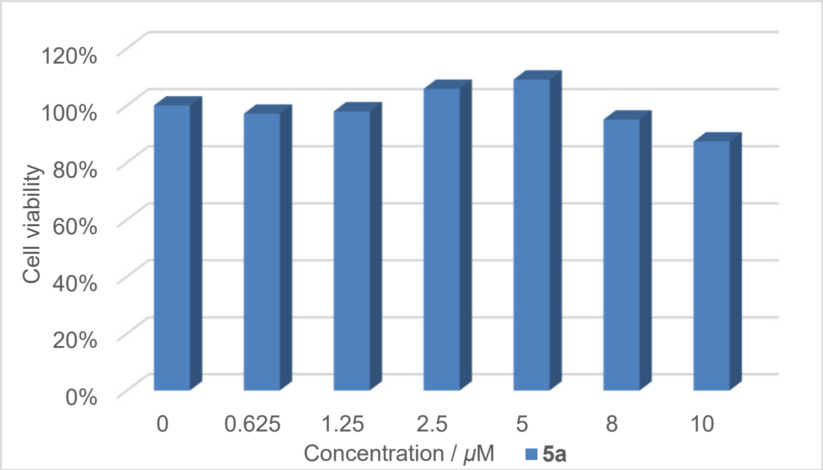

Fig. S16 Cell viability values (%) estimated by CellTiler 96® AQueous One Solution Cell Proliferation Assay, employing HepG2 cells stained with 0−10 μM of **5a** at 37 °C for 24 h.

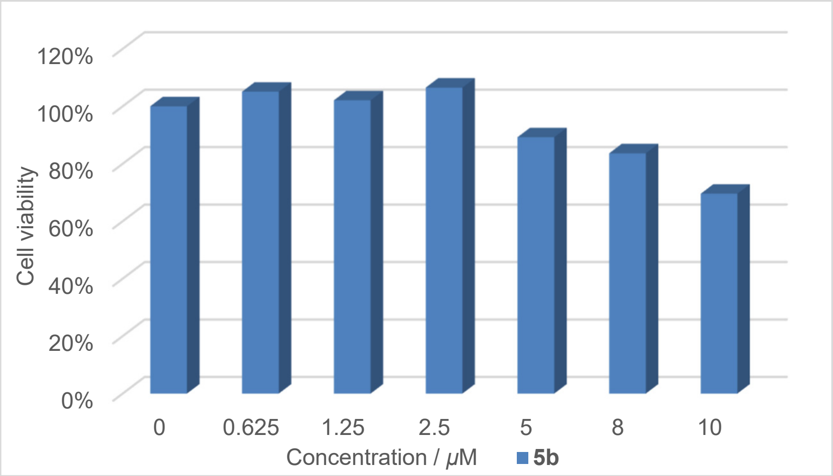

Fig. S17 Cell viability values (%) estimated by CellTiler 96® AQueous One Solution Cell Proliferation Assay, employing HepG2 cells stained with 0−10 μM of **5b** at 37 °C for 24 h.

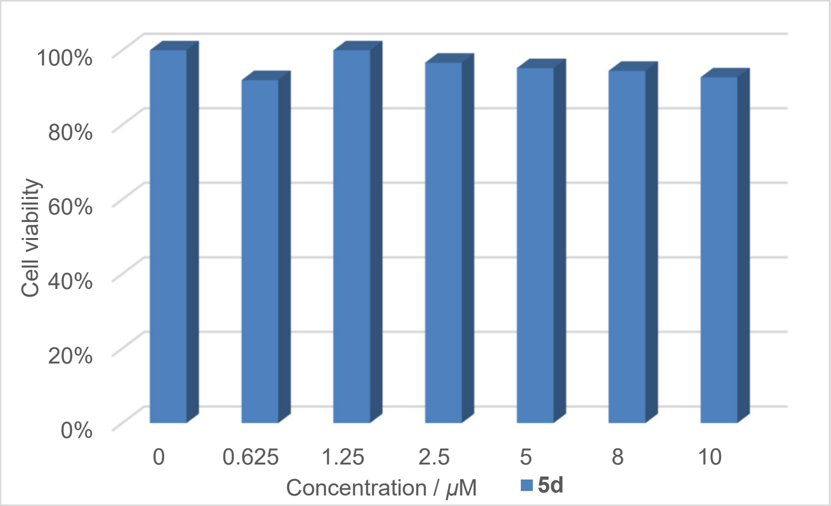

Fig. S18 Cell viability values (%) estimated by CellTiler 96® AQueous One Solution Cell Proliferation Assay, employing HepG2 cells stained with 0−10 μM of **5d** at 37 °C for 24 h.

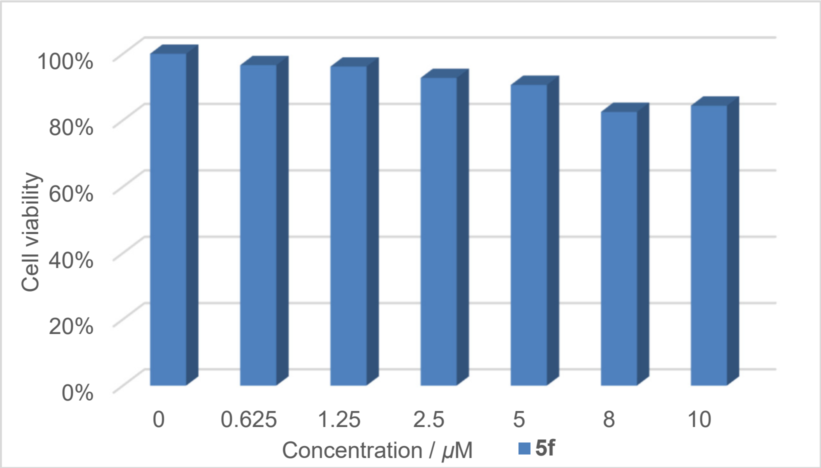

Fig. S19 Cell viability values (%) estimated by CellTiler 96® AQueous One Solution Cell

Proliferation Assay, employing HepG2 cells stained with 0−10 μM of **5f** at 37 °C for 24 h.

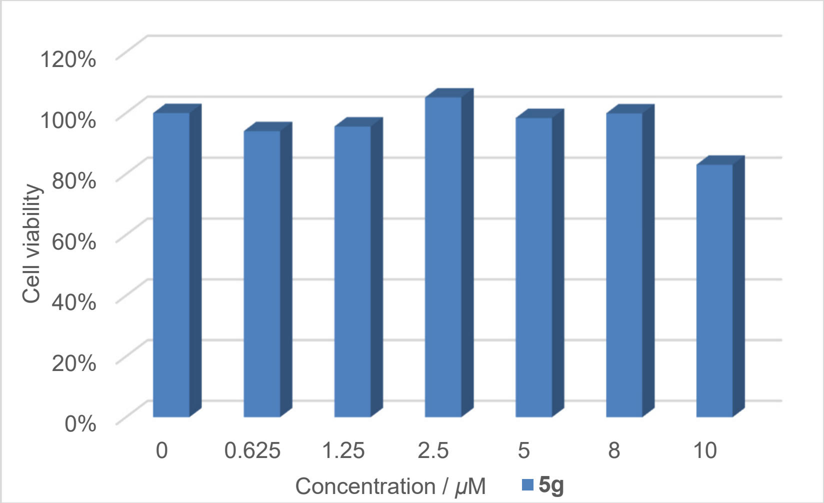

Fig. S20 Cell viability values (%) estimated by CellTiler 96® AQueous One Solution Cell Proliferation Assay, employing HepG2 cells stained with 0−10 μM of **5g** at 37 °C for 24 h.

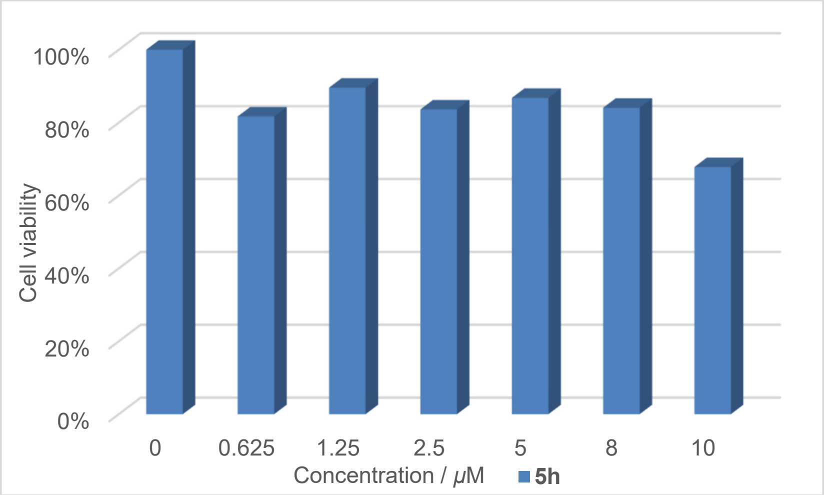

Fig. S21 Cell viability values (%) estimated by CellTiler 96® AQueous One Solution Cell Proliferation Assay, employing HepG2 cells stained with 0−10 μM of **5h** at 37 °C for 24 h.

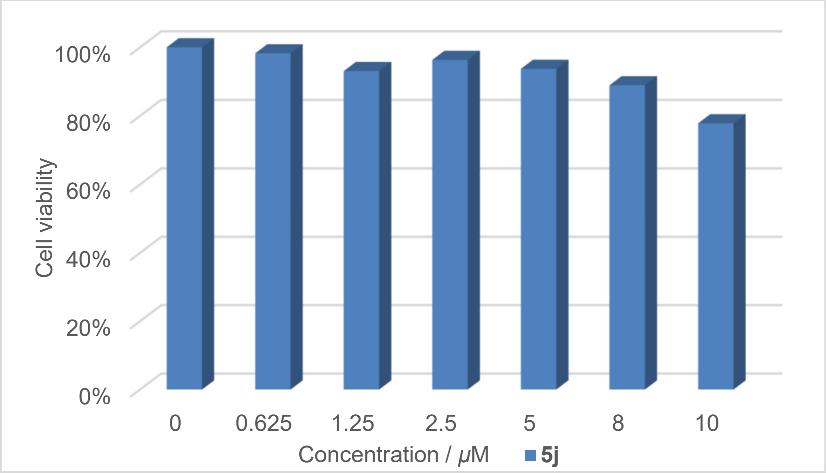

Fig. S22 Cell viability values (%) estimated by CellTiler 96® AQueous One Solution Cell Proliferation Assay, employing HepG2 cells stained with 0–10 μM of **5j** at 37 °C for 24 h.

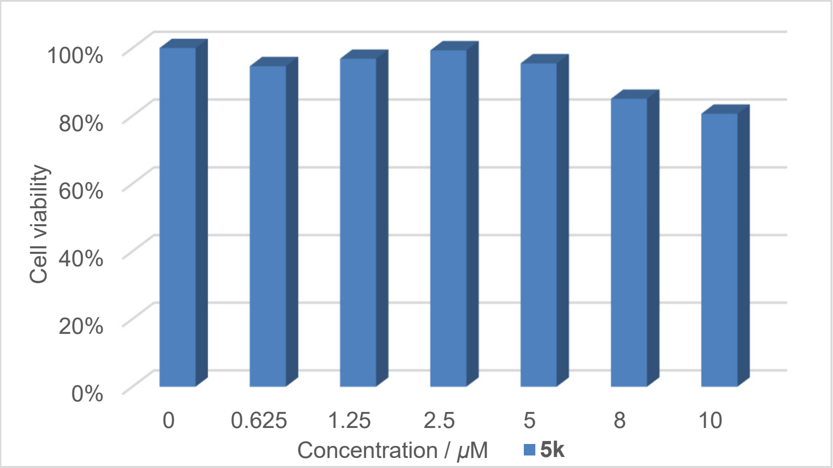

Fig. S23 Cell viability values (%) estimated by CellTiler 96® AQueous One Solution Cell Proliferation Assay, employing HepG2 cells stained with 0–10 μM of **5k** at 37 °C for 24 h.

X. Preparation and characterization of the described compounds

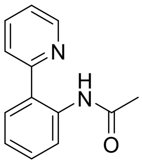

N-Acetyl-(2-pyridin-2-yl)aniline (1b)

Following general procedures for the synthesis of *N*-acetyl-2-(pyridin-2-yl)aniline derivatives, compound **1b** was obtained as a deep-yellow liquid (197.4 mg) in a total yield of 61%. ^{1}H NMR (400 MHz, $CDCl_3$): δ = 2.18 (s, 3H), 7.16 (td, J = 7.6 Hz, 1.2 Hz, 1H), 7.27-7.30 (m, 1H), 7.39-7.43 (m, 1H), 7.64 (dd, J = 8.0 Hz, 1.6 Hz, 1H), 7.74 (d, J = 8.4 Hz, 1H), 7.85 (td, J = 7.6 Hz, 2.0 Hz, 1H), 8.52 (d, J = 8.4 Hz, 1H), 8.64-8.66 (m, 1H), 12.07 (s, 1H) ppm. ^{13}C NMR (100 MHz, $CDCl_3$): δ = 25.4, 122.0, 122.1, 123.3, 123.6, 125.7, 129.0, 130.2, 137.7, 137.9, 147.5, 158.5, 168.7 ppm. ESI-HRMS: calcd for $C_{13}H_{12}N_2ONa$ $[M+Na]^+$ 235.0842, found 235.0842.

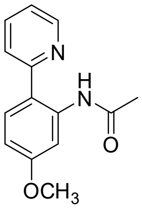

_N_-Acetyl-5-methoxy-(2-pyridin-2-yl)aniline (1c)

Following general procedures for the synthesis of *N*-acetyl-2-(pyridin-2-yl)aniline derivatives, compound **1c** was obtained as a pale-yellow solid (220.8 mg) in a total yield of 49%. ^{1}H NMR (400 MHz, $CDCl_3$): *δ* = 2.20 (s, 3H), 3.88 (s, 3H), 6.71 (dd, *J* = 8.4 Hz, 2.4 Hz, 1H), 7.20-7.23 (m, 1H), 7.60 (d, *J* = 8.8 Hz, 1H), 7.68 (d, *J* = 8.4 Hz, 1H), 7.77-7.82 (m, 1H), 8.30 (d, *J* = 2.4 Hz, 1H), 8.58-8.60 (m, 1H), 12.59 (s, 1H) ppm. ^{13}C NMR (100 MHz, $CDCl_3$): *δ* = 25.6, 55.6, 105.9, 110.4, 117.7, 121.2, 122.3, 129.8, 137.7, 139.6, 147.3, 158.4, 161.1, 169.0 ppm. ESI-HRMS: calcd for $C_{14}H_{15}N_2O_2$ $[M+H]^+$ 243.1128, found 243.1133.

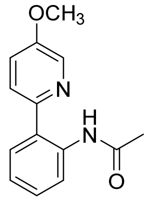

_N_-Acetyl-[2-(5-methoxypyridin-2-yl)]aniline (1d)

Following general procedures for the synthesis of *N*-acetyl-2-(pyridin-2-yl)aniline derivatives, compound **1d** was obtained as a pale-yellow solid (229.7 mg) in a total yield of 59%. ^{1}H NMR (400 MHz, $CDCl_3$): *δ* = 2.17 (s, 3H), 3.93 (s, 3H), 7.14 (td, *J* = 7.6 Hz, 1.2 Hz, 1H), 7.34-7.38 (m, 2H), 7.57 (dd, *J* = 7.6 Hz, 1.2 Hz, 1H), 7.67 (d, *J* = 8.8 Hz, 1H), 8.33 (dd, *J* = 2.8 Hz, 0.4 Hz, 1H), 8.49 (d, *J* = 8.4 Hz, 1H), 11.88 (s, 1H) ppm. ^{13}C NMR (100 MHz, $CDCl_3$): *δ* = 25.4, 55.9, 122.0, 122.6, 123.6, 123.9, 125.7, 128.5, 129.4, 134.8, 137.1, 150.8, 154.5, 168.7 ppm. ESI-HRMS: calcd for $C_{14}H_{15}N_2O_2$ $[M+H]^+$ 243.1128, found 243.1124.

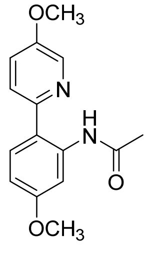

_N_-Acetyl-5-methoxy-[2-(5-methoxypyridin-2-yl)]aniline (1e)

Following general procedures for the synthesis of *N*-acetyl-2-(pyridin-2-yl)aniline

derivatives, compound **1e** was obtained as a pale-yellow solid (255.1 mg) in a total yield of 56%. ^{1}H NMR (400 MHz, $CDCl_3$): δ = 2.19 (s, 3H), 3.86 (s, 3H), 3.92 (s, 3H), 6.69 (dd, J = 8.8 Hz, 2.8 Hz, 1H), 7.34 (dd, J = 8.8 Hz, 2.8 Hz, 1H), 7.51 (d, J = 8.8 Hz, 1H), 7.61 (d, J = 8.8 Hz, 1H), 8.25 (d, J = 2.8 Hz, 1H), 8.28 (dd, J = 2.8 Hz, 0.4 Hz, 1H), 12.30 (s, 1H) ppm. ^{13}C NMR (100 MHz, $CDCl_3$): δ = 25.6, 55.6, 55.9, 105.9, 110.3, 117.9, 122.9, 123.0, 129.3, 134.3, 138.8, 150.9, 154.0, 160.4, 168.9 ppm. ESI-HRMS: calcd for $C_{15}H_{17}N_2O_3$ $[M+H]^+$ 273.1234, found 273.1236.

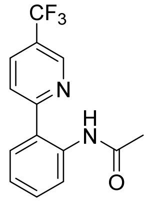

_N_-Acetyl-2-[5-(trifluoromethyl)pyridin-2-yl]aniline (1f)

Following general procedures for the synthesis of *N*-acetyl-2-(pyridin-2-yl)aniline derivatives, compound **1f** was obtained as a pale-yellow solid (257.8 mg) in a total yield of 44%. ^{1}H NMR (400 MHz, $CDCl_3$): δ = 2.19 (s, 3H), 7.20 (t, J = 7.6 Hz, 1H), 7.47 (t, J = 7.6 Hz, 1H), 7.66 (d, J = 7.6 Hz, 1H), 7.87 (d, J = 8.4 Hz, 1H), 8.08 (d, J = 8.4 Hz, 1H), 8.53 (d, J = 8.0 Hz, 1H), 8.92 (s, 1H), 11.70 (s, 1H) ppm. ^{13}C NMR (100 MHz, $CDCl_3$): δ = 25.4, 122.5, 123.0, 123.5 (q, J = 270.3 Hz), 123.9, 124.6, 129.4, 131.3, 134.9 (q, J = 3.3 Hz), 137.9, 144.7 (q, J = 4.0 Hz), 161.8, 168.8 ppm. ^{19}F NMR (376 MHz, $CDCl_3$): δ = -62.37 ppm. ESI-HRMS: calcd for $C_{14}H_{11}F_3N_2ONa$ $[M+Na]^+$ 303.0716, found 303.0712.

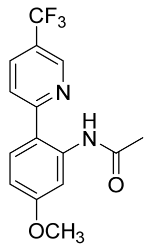

_N_-Acetyl-5-methoxy-2-[5-(trifluoromethyl)pyridin-2-yl]aniline (1g)

Following general procedures for the synthesis of *N*-acetyl-2-(pyridin-2-yl)aniline derivatives, compound **1g** was obtained as a pale-yellow solid (285.5 mg) in a total

yield of 46%. ^{1}H NMR (400 MHz, $CDCl_3$): δ = 2.21 (s, 3H), 3.88 (s, 3H), 6.72 (dd, J = 9.2 Hz, 2.8 Hz, 1H), 7.62 (d, J = 8.8 Hz, 1H), 7.80 (d, J = 8.4 Hz, 1H), 7.99-8.02 (m, 1H), 8.30 (d, J = 2.4 Hz, 1H), 8.85 (dd, J = 1.2 Hz, 0.8 Hz, 1H), 12.29 (s, 1H) ppm. ^{13}C NMR (100 MHz, $CDCl_3$): δ = 25.6, 55.6, 106.0, 110.7, 121.9, 123.6 (q, J = 270.4 Hz), 123.8 (q, J = 33.4 Hz), 130.3, 134.6 (q, J = 3.2 Hz), 140.1, 144.4 (q, J = 4.0 Hz), 161.6, 162.0, 169.0 ppm. ^{19}F NMR (376 MHz, $CDCl_3$): δ = -62.32 ppm. ESI-HRMS: calcd for $C_{15}H_{13}F_3N_2O_2Na$ $[M+Na]^+$ 333.0821, found 333.0826.

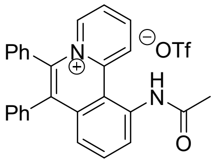

11-Acetamido-6,7-diphenylphenanthridizin-5-ium trifluoromethanesulfonate (3c)

Compound **3c** was obtained as a grayish-white solid (36.7 mg) in 68% yield *via* Rh-catalyzed C–H activation/cyclization of 2-(pyridin-2-yl)aniline **1a** with diphenylacetylene **2a**. ^{1}H NMR (400 MHz, $CDCl_3$): δ = 2.42 (s, 3H), 7.13-7.15 (m, 2H), 7.30-7.33 (m, 5H), 7.39 (dd, J = 8.0 Hz, 0.8 Hz, 1H), 7.43-7.44 (m, 3H), 7.75 (td, J = 7.2 Hz, 1.6 Hz, 1H), 7.85 (t, J = 8.0 Hz, 1H), 8.22 (d, J = 7.2 Hz, 1H), 8.35-8.40 (m, 1H), 8.67 (d, J = 6.8 Hz, 1H), 9.82 (d, J = 9.2 Hz, 1H), 10.47 (s, 1H) ppm. ^{13}C NMR (100 MHz, $CDCl_3$): δ = 24.2, 120.1, 123.6, 125.0, 126.7, 128.7, 128.8, 130.0, 130.1, 130.3, 130.6, 130.7, 130.9, 133.6, 134.0, 134.3, 135.4, 137.1, 137.2, 137.4, 138.4, 144.4, 170.8 ppm. ^{19}F NMR (376 MHz, $CDCl_3$): δ = -78.43 ppm. ESI-HRMS: calcd for $C_{27}H_{21}N_2O$ $[M]^+$ 389.1648, found 389.1644.

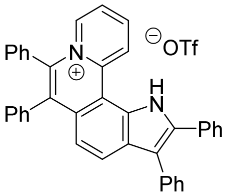

2,3,6,7-Tetraphenyl-1*H*-pyrrolo[3,2-*k*]phenanthridizin-8-ium trifluoromethanesulfonate (4a)

Compound **4a** was obtained as a yellow solid (55.2 mg) in 82% yield *via* Rh-catalyzed dual C–H activation/cyclization of *N*-acetyl-2-(pyridin-2-yl)aniline **1b** with

diphenylacetylene **2a**. ^1H NMR (400 MHz, DMSO-d_6): δ = 7.24 (d, J = 8.8 Hz, 1H), 7.28-7.53 (m, 16H), 7.55-7.57 (m, 2H), 7.69-7.71 (m, 2H), 8.10 (td, J = 6.8 Hz, 1.2 Hz, 1H), 8.20 (d, J = 8.8 Hz, 1H), 8.73 (td, J = 8.4 Hz, 1.2 Hz, 1H), 8.83 (d, J = 6.8 Hz, 1H), 9.86 (d, J = 8.8 Hz, 1H), 12.81 (s, 1H) ppm. ^{13}C NMR (100 MHz, DMSO-d_6): δ = 109.6, 113.0, 116.4, 119.0, 123.6, 126.7, 126.8, 127.1, 128.2, 128.3, 128.6, 128.7, 129.0, 129.3, 129.5, 129.8, 129.96, 130.04, 130.11, 130.15, 131.19, 131.21, 131.7, 133.3, 135.5, 136.0, 136.1, 136.3, 138.7, 139.4, 140.3 ppm. ^{19}F NMR (376 MHz, DMSO-d_6): δ = -77.74 ppm. ESI-HRMS: calcd for $C_{39}H_{27}N_2$ $[M]^+$ 523.2169, found 523.2162.

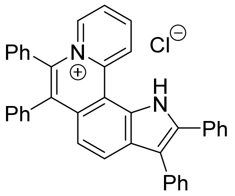

2,3,6,7-Tetraphenyl-1*H*-pyrrolo[3,2-*k*]phenanthridizin-8-ium chloride (5a)

Following the general procedure for Rh-catalyzed dual C–H activation/cyclization and the subsequent anion exchange reaction, compound **5a** was obtained as a yellow solid (42.5 mg) in a total yield of 76%. ^1H NMR (400 MHz, DMSO-d_6): δ = 7.23 (d, J = 8.4 Hz, 1H), 7.28-7.51 (m, 16H), 7.55-7.57 (m, 2H), 7.70-7.72 (m, 2H), 8.11 (td, J = 7.2 Hz, 1.2 Hz, 1H), 8.20 (d, J = 8.4 Hz, 1H), 8.73 (td, J = 7.2 Hz, 1.2 Hz, 1H), 8.82 (d, J = 7.2 Hz, 1H), 9.88 (d, J = 8.8 Hz, 1H), 12.88 (s, 1H) ppm. ^{13}C NMR (100 MHz, DMSO-d_6): δ = 109.6, 113.0, 116.4, 118.9, 123.6, 126.67, 127.1, 128.1, 128.2, 128.5, 128.6, 128.9 , 129.3, 129.4, 129.8, 129.9, 130.00, 130.02, 130.11, 130.13, 130.15, 131.2, 131.6, 133.3, 135.5, 135.9, 136.1, 136.2, 138.7, 139.4, 140.2 ppm. ESI-HRMS: calcd for $C_{39}H_{27}N_2$ $[M]^+$ 523.2169, found 523.2168.

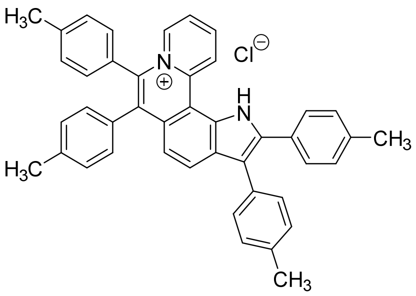

2,3,6,7-Tetra(4-tolyl)-1*H*-pyrrolo[3,2-*k*]phenanthridizin-8-ium chloride (5b)

Following the general procedure for Rh-catalyzed dual C–H activation/cyclization and the subsequent anion exchange reaction, compound **5b** was obtained as a yellow solid (46.6 mg) in a total yield of 76%. ^{1}H NMR (400 MHz, DMSO-d_6): δ = 2.30 (s, 3H), 2.34 (s, 3H), ,2.36 (s, 3H), 2.38 (s, 3H), 7.16-7.21 (m, 5H), 7.24-7.32 (m, 8H), 7.43 (d, J = 8.0 Hz, 2H), 7.58 (d, J = 8.0 Hz, 2H), 8.07 (t, J = 7.2 Hz, 1H), 8.15 (d, J = 8.8 Hz, 1H), 8.69 (t, J = 8.4 Hz, 1H), 8.76 (d, J = 6.8 Hz, 1H), 9.83 (d, J = 8.4 Hz, 1H), 12.69 (s, 1H) ppm. ^{13}C NMR (100 MHz, $CDCl_3$): δ = 21.4, 21.5, 21.6, 113.0, 116.7, 119.1, 122.6, 127.2, 127.7, 128.0, 128.4, 129.2, 129.3, 129.5, 129.6, 130.0, 130.3, 130.68, 130.75, 130.8, 131.0, 132.1, 132.3, 134.3, 135.2, 136.7, 137.9, 138.2, 138.6, 139.4, 140.6, 140.7, 141.5 ppm. ESI-HRMS: calcd for $C_{43}H_{35}N_2$ $[M]^+$ 579.2795, found 579.2792.

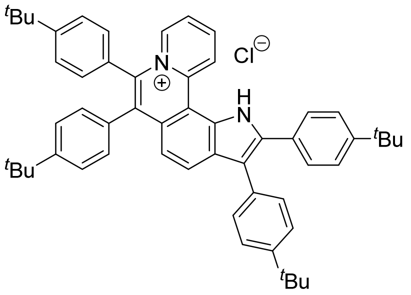

2,3,6,7-Tetra[4-(*tert*-butyl)phenyl]-1*H*-pyrrolo[3,2-*k*]phenanthridizin-8-ium chloride (5c)

Following the general procedure for Rh-catalyzed dual C–H activation/cyclization and the subsequent anion exchange reaction, compound **5c** was obtained as a yellow solid (56.2 mg) in a total yield of 72%. ^{1}H NMR (400 MHz, DMSO-d_6): δ = 1.22 (s, 9H), 1.24 (s, 9H), 1.33 (s, 9H), 1.34 (s, 9H), 7.14 (d, J = 8.0 Hz, 2H), 7.31-7.54 (m, 13H), 7.65 (d, J = 8.4 Hz, 2H), 8.11 (t, J = 6.8 Hz, 1H), 8.20 (d, J = 8.8 Hz, 1H), 8.69 (t, J = 7.6 Hz, 1H), 8.95 (d, J = 6.8 Hz, 1H), 9.82 (br, 1H), 12.67 (s, 1H) ppm. ^{13}C NMR (100 MHz, $CDCl_3$): δ = 31.2, 31.3, 31.4, 31.6, 34.71, 34.74, 34.8, 35.0, 113.3, 116.7, 118.9, 122.6, 125.1, 125.6, 125.7, 126.6, 127.7, 128.2, 128.3, 128.6, 129.3, 129.9, 130.2, 130.8, 131.0, 131.3, 132.4, 132.6, 133.9, 135.0, 138.1, 138.6, 141.0, 141.3, 149.8, 151.2, 151.3, 153.5 ppm. ESI-HRMS: calcd for $C_{55}H_{59}N_2$ $[M]^+$ 747.4673, found 747.4672.

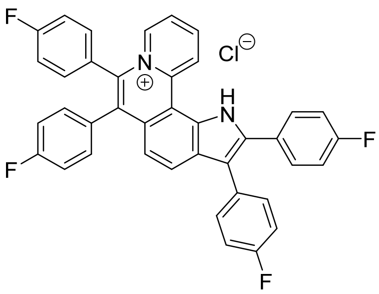

2,3,6,7-Tetra(4-fluorophenyl)-1*H*-pyrrolo[3,2-*k*]phenanthridizin-8-ium chloride (5d)

Following the general procedure for Rh-catalyzed dual C–H activation/addition/cyclization and the subsequent anion exchange reaction, compound **5d** was obtained as a yellow solid (41.7 mg) in a total yield of 66%. ^{1}H NMR (400 MHz, DMSO-d_6): δ = 7.21-7.40 (m, 11H), 7.42-7.46 (m, 2H), 7.62-7.65 (m, 2H), 7.73-7.76 (m, 2H), 8.10 (td, *J* = 7.2 Hz, 1.2 Hz, 1H), 8.18 (d, *J* = 8.8 Hz, 1H), 8.74 (td, *J* = 7.6 Hz, 1.2 Hz, 1H), 8.88 (d, *J* = 6.8 Hz, 1H), 9.92 (d, *J* = 7.6 Hz, 1H), 12.94 (s, 1H) ppm. ^{13}C NMR (100 MHz, DMSO-d_6): δ = 109.6, 113.0, 115.3, 115.4 (d, *J* = 21.7 Hz), 115.6 (d, *J* = 21.5 Hz), 116.0 (d, *J* = 21.3 Hz), 116.5 (d, *J* = 21.9 Hz), 118.8, 123.6, 126.6 (d, *J* = 5.0 Hz), 127.5, 128.0 (d, *J* = 3.4 Hz), 128.2, 129.3, 129.5 (d, *J* = 2.1 Hz), 130.1, 130.2, 130.9, 131.8 (d, *J* = 3.3 Hz), 131.9 (d, *J* = 8.2 Hz), 132.2 (d, *J* = 8.2 Hz), 133.8 (d, *J* = 8.7 Hz), 135.6 (d, *J* = 8.2 Hz), 136.3, 138.7, 138.9, 140.3, 161.3 (d, *J* = 243.0 Hz), 161.6 (d, *J* = 244.3 Hz), 162.2 (d, *J* = 245.3 Hz), 162.6 (d, *J* = 246.7 Hz) ppm. ^{19}F NMR (376 MHz, DMSO-d_6): δ = -110.57, -112.56, -113.30, -115.12 ppm. ESI-HRMS: calcd for $C_{39}H_{23}F_4N_2$ $[M]^+$ 595.1792, found 595.1787.

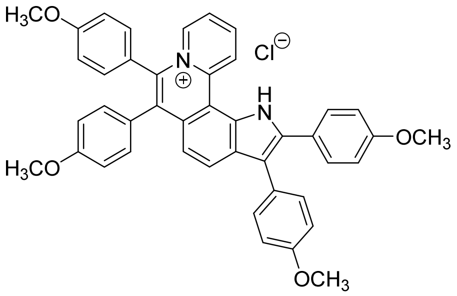

2,3,6,7-Tetra(4-methoxyphenyl)-1*H*-pyrrolo[3,2-*k*]phenanthridizin-8-ium chloride (5e)

Following the general procedure for Rh-catalyzed dual C–H activation/cyclization and

the subsequent anion exchange reaction, compound **5e** was obtained as a yellow solid (53.1 mg) in a total yield of 78%. ^{1}H NMR (400 MHz, DMSO-d_6): δ = 3.76 (s, 3H), 3.79 (s, 3H), 3.80 (s, 3H), 3.82 (s, 3H), 6.94 (d, J = 8.8 Hz, 2H), 7.02-7.07 (m, 6H), 7.19-7.24 (m, 3H), 7.32 (d, J = 8.8 Hz, 2H), 7.46 (d, J = 8.8 Hz, 2H), 7.63 (d, J = 8.8 Hz, 2H), 8.08 (t, J = 6.8 Hz, 1H), 8.12 (d, J = 8.4 Hz, 1H), 8.69 (t, J = 8.4 Hz, 1H), 8.83 (d, J = 6.8 Hz, 1H), 9.83 (d, J = 8.8 Hz, 1H), 12.66 (s, 1H) ppm. ^{13}C NMR (100 MHz, DMSO-d_6): δ = 55.07, 55.09, 55.2, 55.3, 109.6, 112.7, 113.7, 114.0, 114.4, 114.8, 115.3, 118.7, 123.3, 123.6, 123.8, 125.5, 126.4, 126.6, 127.8, 129.6, 129.9, 130.5, 130.9, 131.1, 131.3, 132.6, 135.8, 136.3, 138.4, 139.1, 140.2, 158.2, 158.6, 159.5, 159.8 ppm. ESI-HRMS: calcd for $C_{43}H_{35}N_2O_4$ $[M]^+$ 643.2591, found 643.2584.

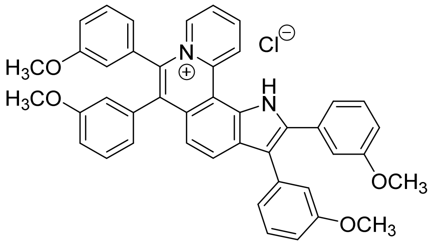

2,3,6,7-Tetra(3-methoxyphenyl)-1*H*-pyrrolo[3,2-*k*]phenanthridizin-8-ium chloride (5f)

Following the general procedure for Rh-catalyzed dual C–H activation/cyclization and the subsequent anion exchange reaction, compound **5f** was obtained as a yellow solid (39.5 mg) in a total yield of 58%. ^{1}H NMR (400 MHz, DMSO-d_6): δ = 3.69 (d, J = 3.2 Hz, 3H), 3.72 (d, J = 7.2 Hz, 3H), 3.74 (s, 3H), 3.76 (s, 3H), 6.86-7.44 (m, 17H), 8.10 (t, J = 7.2 Hz, 1H), 8.24 (d, J = 8.8 Hz, 1H), 8.73 (t, J = 8.4 Hz, 1H), 8.86 (d, J = 7.2 Hz, 1H), 9.86 (d, J = 8.4 Hz, 1H), 12.80 (s, 1H) ppm. ^{13}C NMR (100 MHz, DMSO-d_6): δ = 55.06, 55.14 (d, J = 3.2 Hz), 55.2, 55.3 (d, J = 5.1 Hz), 109.6, 112.7, 112.9, 113.5, 113.7, 113.9, 115.47, 115.52, 115.8, 115.9, 116.1, 116.4, 116.7, 116.8, 119.0, 122.1, 122.4, 123.16, 123.23, 123.6, 126.7, 126.9, 129.3, 129.5, 129.7, 130.0, 130.1, 130.63, 130.64, 132.4, 132.8, 134.7, 135.7, 135.9, 136.0, 136.7, 138.8, 139.2, 140.2, 158.8, 159.1, 159.5, 159.6 ppm. ESI-HRMS: calcd for $C_{43}H_{35}N_2O_4$ $[M]^+$ 643.2591, found 643.2592.

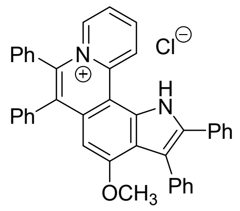

4-Methoxy-2,3,6,7-tetraphenyl-1*H*-pyrrolo[3,2-*k*]phenanthridizin-8-ium chloride (5g)

Following the general procedure for Rh-catalyzed dual C–H activation/cyclization and the subsequent anion exchange reaction, compound **5g** was obtained as a yellow solid (38.1 mg) in a total yield of 65%. ^{1}H NMR (400 MHz, DMSO-d_6): δ = 3.50 (s, 3H), 6.49 (s, 1H), 7.30-7.50 (m, 16H), 7.54-7.57 (m, 4H), 7.94 (td, J = 7.2 Hz, 1.2 Hz, 1H), 8.58 (td, J = 8.4 Hz, 1.2 Hz, 1H), 8.69 (d, J = 6.8 Hz, 1H), 9.70 (d, J = 8.8 Hz, 1H), 12.80 (s, 1H) ppm. ^{13}C NMR (100 MHz, DMSO-d_6): δ = 55.2, 97.7, 108.6, 116.4, 120.4, 122.0, 125.7, 126.6, 127.5, 128.2, 128.26, 128.30, 128.7, 129.3, 129.7, 129.87, 129.93, 131.1, 131.2, 131.3, 131.6, 131.8, 132.3, 134.5, 135.1, 135.3, 135.4, 136.7, 137.6, 138.8, 139.6, 159.0 ppm. ESI-HRMS: calcd for $C_{40}H_{29}N_2O$ $[M]^+$ 553.2274, found 553.2273.

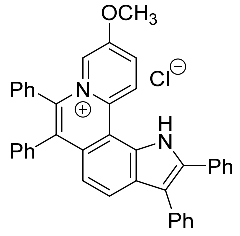

10-Methoxy-2,3,6,7-tetraphenyl-1*H*-pyrrolo[3,2-*k*]phenanthridizin-8-ium chloride (5h)

Following the general procedure for Rh-catalyzed dual C–H activation/cyclization and the subsequent anion exchange reaction, compound **5h** was obtained as a yellow solid (35.8 mg) in a total yield of 61%. ^{1}H NMR (400 MHz, DMSO-d_6): δ = 3.86 (s, 3H), 7.20 (d, J = 8.8 Hz, 1H), 7.29-7.52 (m, 16H), 7.57 (dd, J = 7.6 Hz, 1.2 Hz, 2H), 7.69 (dd, J = 8.0 Hz, 1.2 Hz, 2H), 8.12-8.15 (m, 2H), 8.53 (dd, J = 9.6 Hz, 2.4 Hz, 1H), 9.88 (d, J = 9.6 Hz, 1H), 12.79 (s, 1H) ppm. ^{13}C NMR (100 MHz, $CDCl_3$): δ = 57.3, 113.5, 117.1, 118.9, 119.3, 121.1, 122.1, 126.5, 127.1, 128.32, 128.35, 128.46, 128.48, 128.52,

128.7, 128.9, 129.5, 130.1, 130.2, 130.50, 130.53, 130.7, 131.0, 131.5, 131.9, 134.0, 134.5, 135.3, 136.0, 138.2, 140.6, 155.2 ppm. ESI-HRMS: calcd for $C_{40}H_{29}N_2O$ $[M]^+$ 553.2274, found 553.2272.

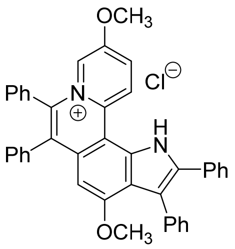

4,10-Dimethoxy-2,3,6,7-tetraphenyl-1*H*-pyrrolo[3,2-*k*]phenanthridizin-8-ium chloride (5i)

Following the general procedure for Rh-catalyzed dual C–H activation/cyclization and the subsequent anion exchange reaction, compound **5i** was obtained as a yellow solid (34.7 mg) in a total yield of 56%. ^{1}H NMR (400 MHz, DMSO-d_6): δ = 3.48 (s, 3H), 3.81 (s, 3H), 6.46 (s, 1H), 7.32-7.58 (m, 20H), 8.00 (d, J = 2.8 Hz, 1H), 8.42 (dd, J = 10.0 Hz, 2.8 Hz, 1H), 9.68 (d, J = 10.0 Hz, 1H), 12.84 (s, 1H) ppm. ^{13}C NMR (100 MHz, DMSO-d_6): δ = 55.2, 56.6, 97.5, 109.0, 116.5, 118.6, 120.6, 126.7, 127.1, 127.5, 128.20, 128.22, 128.25, 128.31, 128.4, 128.6, 129.5, 129.7, 129.9, 130.1, 131.0, 131.1, 131.2, 131.3, 131.8, 134.6, 135.1, 135.5, 135.8, 136.2, 138.9, 153.1, 158.3 ppm. ESI-HRMS: calcd for $C_{41}H_{31}N_2O_2$ $[M]^+$ 583.2380, found 583.2383.

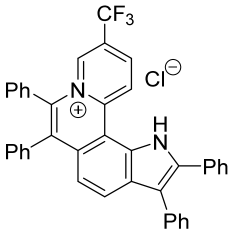

2,3,6,7-Tetraphenyl-10-trifluoromethyl-1*H*-pyrrolo[3,2-*k*]phenanthridizin-8-ium chloride (5j)

Following the general procedure for Rh-catalyzed dual C–H activation/cyclization and the subsequent anion exchange reaction, compound **5j** was obtained as a yellow solid (39.6 mg) in a total yield of 63%. ^{1}H NMR (400 MHz, DMSO-d_6): δ = 7.28-7.54 (m, 17H), 7.58-7.60 (m, 2H), 7.70-7.72 (m, 2H), 8.33 (d, J = 8.8 Hz, 1H), 8.86 (s, 1H), 9.05

(dd, J = 9.2 Hz, 1.6 Hz, 1H), 10.03 (d, J = 9.2 Hz, 1H), 13.10 (s, 1H) ppm. ^{13}C NMR (100 MHz, DMSO-d_6): δ = 112.7, 116.7, 119.0, 120.6, 123.2 (q, J = 34.0 Hz), 127.3, 128.45, 128.50, 128.6, 128.8, 129.0, 129.4, 129.8, 129.9, 130.0, 130.2, 130.4, 130.6, 130.7, 131.0, 131.3, 133.0, 133.1, 135.0, 137.1, 137.3, 139.9, 141.9 ppm. ^{19}F NMR (376 MHz, DMSO-d_6): δ = -62.11 ppm. ESI-HRMS: calcd for $C_{40}H_{26}F_3N_2$ [M]$^+$ 591.2043, found 591.2040.

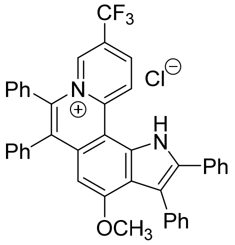

4-Methoxy-2,3,6,7-tetraphenyl-10-trifluoromethyl-1*H*-pyrrolo[3,2-*k*]phenanthridizin-8-ium chloride (5k)

Following the general procedure for Rh-catalyzed dual C–H activation/cyclization and the subsequent anion exchange reaction, compound **5k** was obtained as a yellow solid (38.8 mg) in a total yield of 59%. ^{1}H NMR (400 MHz, DMSO-d_6): δ = 3.55 (s, 3H), 6.56 (s, 1H), 7.31-7.45 (m, 13H), 7.49-7.53 (m, 3H), 7.57-7.61 (m, 4H), 8.74 (s, 1H), 8.86 (d, J = 9.6 Hz, 1H), 9.83 (d, J = 9.2 Hz, 1H), 13.08 (s, 1H) ppm. ^{13}C NMR (100 MHz, $CDCl_3$): δ = 55.6, 98.5, 109.4, 117.5, 121.8 (q, J = 271.4 Hz), 122.5, 124.2 (q, J = 35.6 Hz), 126.8, 127.7, 128.3, 128.4, 128.8, 129.0, 129.8, 129.9, 130.2, 130.7, 130.79, 130.85, 131.0, 131.1, 131.5, 132.2, 132.5, 133.6, 134.95, 135.01, 136.2, 137.4, 141.0, 141.4, 161.7 ppm. ^{19}F NMR (376 MHz, $CDCl_3$): δ = -63.15 ppm. ESI-HRMS: calcd for $C_{41}H_{28}F_3N_2O$ [M]$^+$ 621.2148, found 621.2143.

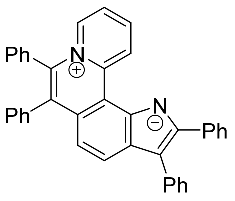

2,3,6,7-Tetraphenylpyrrolo[3,2-*k*]phenanthridizin-8-ium-1-ide (6a)

Compound **6a** was obtained as a dark red solid (46.4 mg) in nearly 100% yield *via* the

reaction of pyrrolo[3,2-*k*]phenanthridizinium **5a** with NaOH solution. ^{1}H NMR (400 MHz, DMSO-d_6): δ = 6.73 (d, J = 8.4 Hz, 1H), 7.18-7.42 (m, 16H), 7.47 (dd, J = 7.2 Hz, 1.2 Hz, 2H), 7.76 (td, J = 7.2 Hz, 1.2 Hz, 1H), 7.87 (d, J = 7.2 Hz, 2H), 7.98 (d, J = 8.4 Hz, 1H), 8.51 (t, J = 6.8 Hz, 2H), 12.29 (d, J = 8.8 Hz, 1H) ppm. ^{13}C NMR (100 MHz, DMSO-d_6): δ = 112.5, 114.0, 114.4, 120.7, 124.8, 125.7, 125.8, 127.2, 127.5, 127.8, 128.0, 128.4, 128.5, 128.7, 129.1, 129.4, 129.9, 130.1, 131.5, 132.5, 132.7, 132.8, 133.6, 135.6, 136.5, 136.8, 138.3, 139.2, 141.5, 141.6, 148.6 ppm. ESI-HRMS: calcd for $C_{39}H_{27}N_2$ $[M+H]^+$ 523.2169, found 523.2170.

XI. References

1 K. Park, G. Bae, J. Moon, J. Choe, K. H. Song and S. Lee, *J. Org. Chem.*, 2010, **75**, 6244–6251.

2 J. Peng, M. Chen, Z. Xie, S. Luo and Q. Zhu, *Org. Chem. Front.*, 2014, **1**, 777–781.

3 C. N. Baki and E. U. Akkaya, *J. Org. Chem.*, 2001, **66**, 1512–1513.

XII. Copies of ^{1}H, ^{13}C and ^{19}F NMR spectra

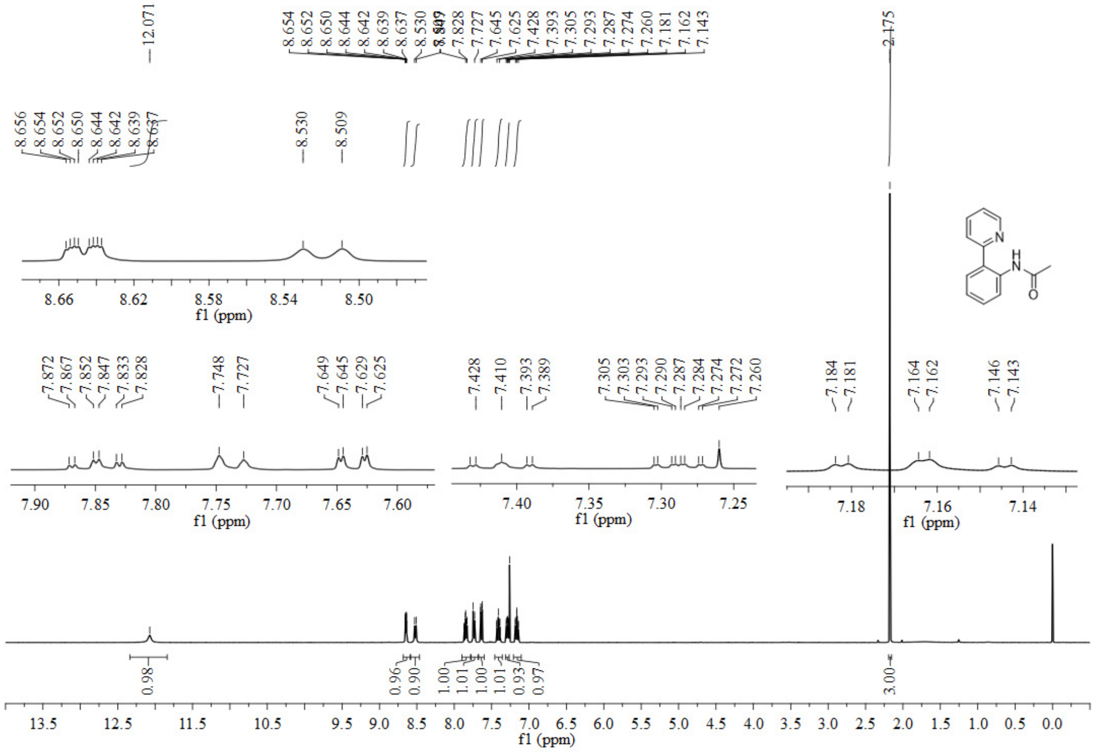

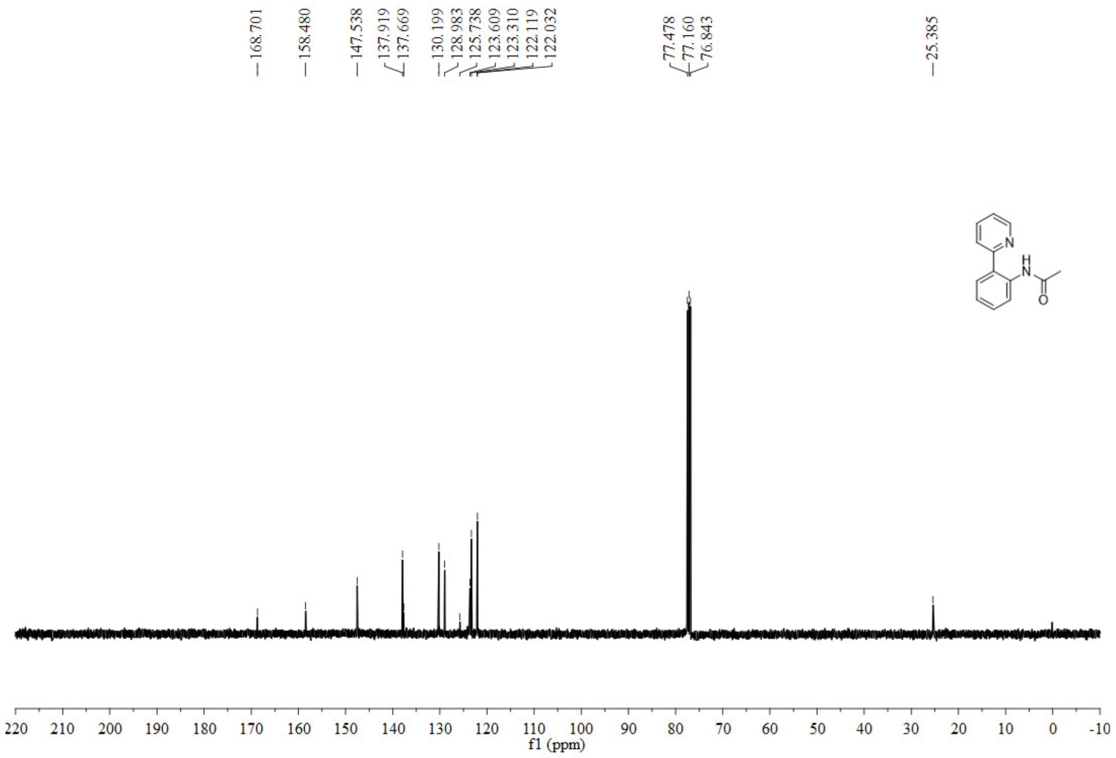

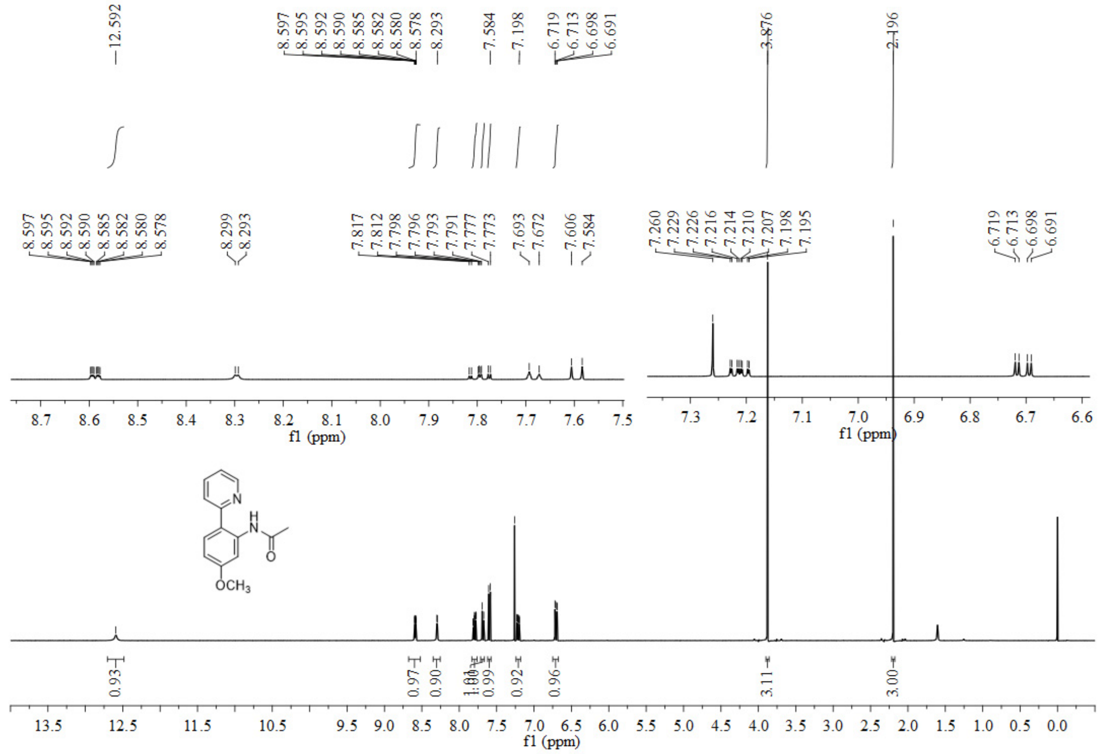
12.592
8.597 8.595 8.592 8.590 8.585 8.582 8.580 8.578 8.293 7.584 7.198 6.719 6.713 6.698 6.691
3.876
2.196
8.597 8.595 8.592 8.590 8.585 8.582 8.580 8.578 8.299 8.293 7.817 7.812 7.798 7.796 7.793 7.791 7.777 7.773 7.693 7.672 7.606 7.584
7.260 7.229 7.226 7.216 7.214 7.210 7.207 7.198 7.195 6.719 6.713 6.698 6.691
f1 (ppm)
N
H
N
O
OCH3
0.93 0.97 0.90 1.00 1.00 0.99 0.92 0.96 3.11 3.00
f1 (ppm)

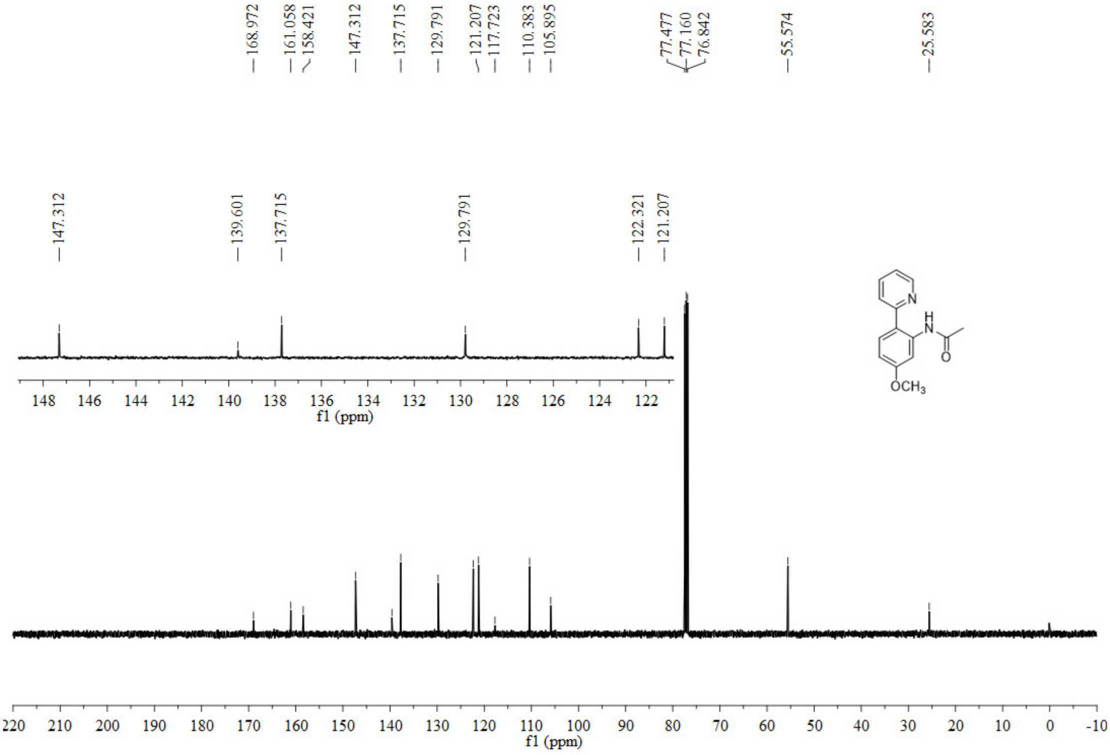
168.972 161.058 158.421 147.312 137.715 129.791 121.207 117.723 110.383 105.895
77.477 77.160 76.842
55.574
25.583
147.312 139.601 137.715 129.791 122.321 121.207
f1 (ppm)
N
H
N
O
OCH3
f1 (ppm)

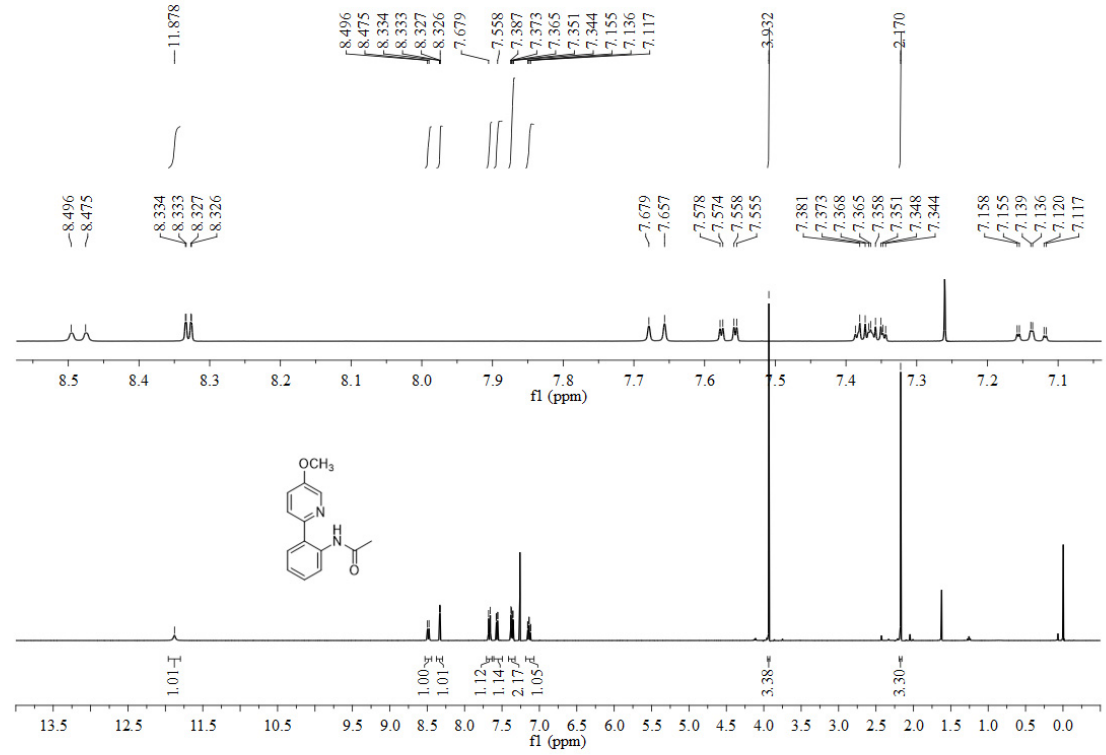

11.878
8.496
8.475
8.334
8.333
8.327
8.326
7.679
7.558
7.387
7.373
7.365
7.351
7.344
7.155
7.136
7.117
3.932
2.170
8.496
8.475
8.334
8.333
8.327
8.326
7.679
7.657
7.578
7.574
7.558
7.555
7.381
7.373
7.368
7.365
7.358
7.351
7.348
7.344
7.158
7.155
7.139
7.136
7.120
7.117
OCH3
N
H
O
1.01
1.00
1.01
1.12
1.14
2.17
1.05
3.38
3.30
f1 (ppm)

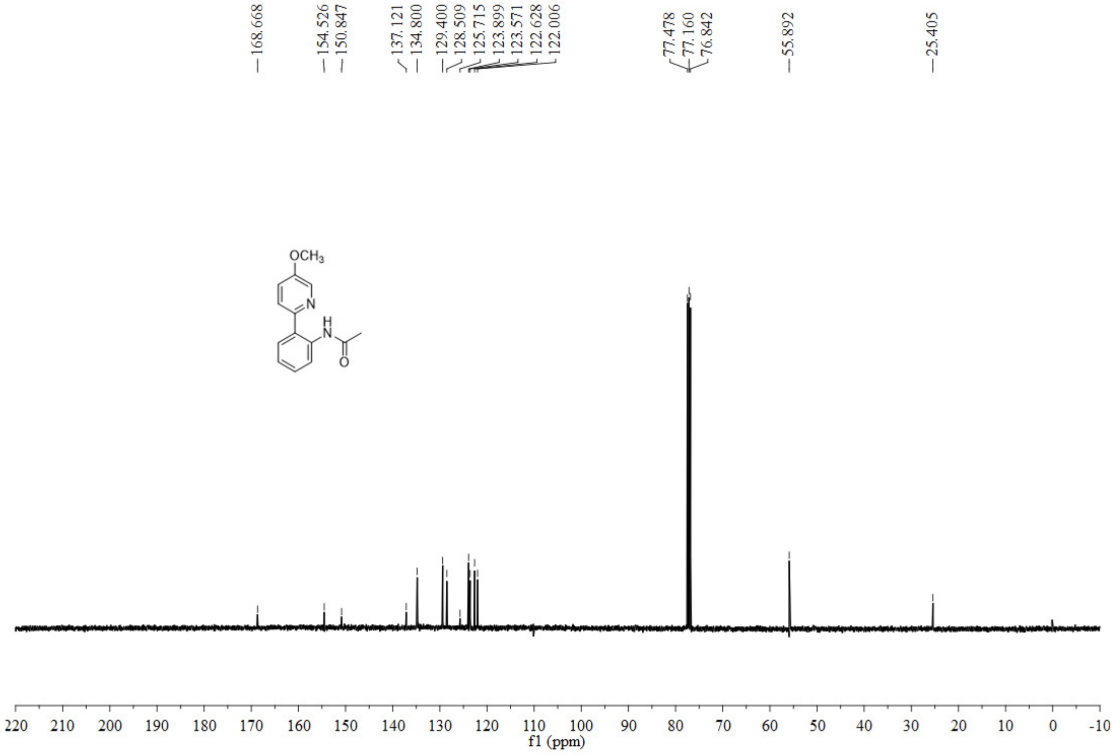

168.668
154.526
150.847
137.121
134.800
129.400
128.509
125.715
123.899
123.571
122.628
122.006
77.478
77.160
76.842
55.892
25.405
OCH3
N
H
O
f1 (ppm)

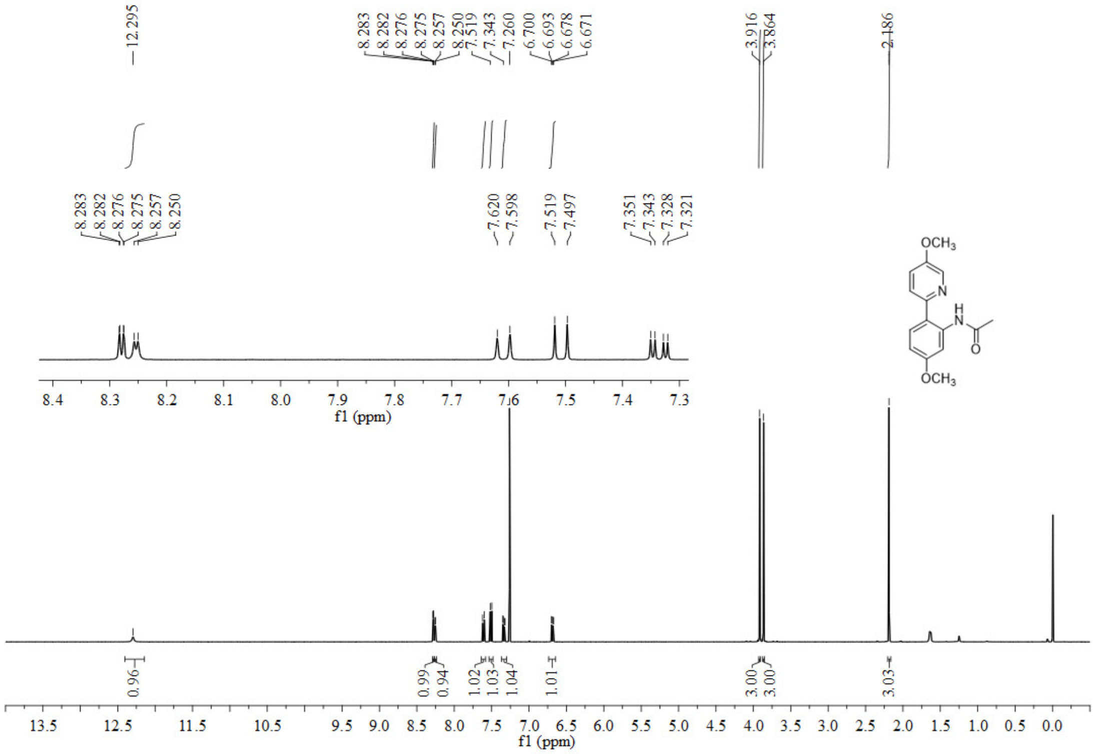

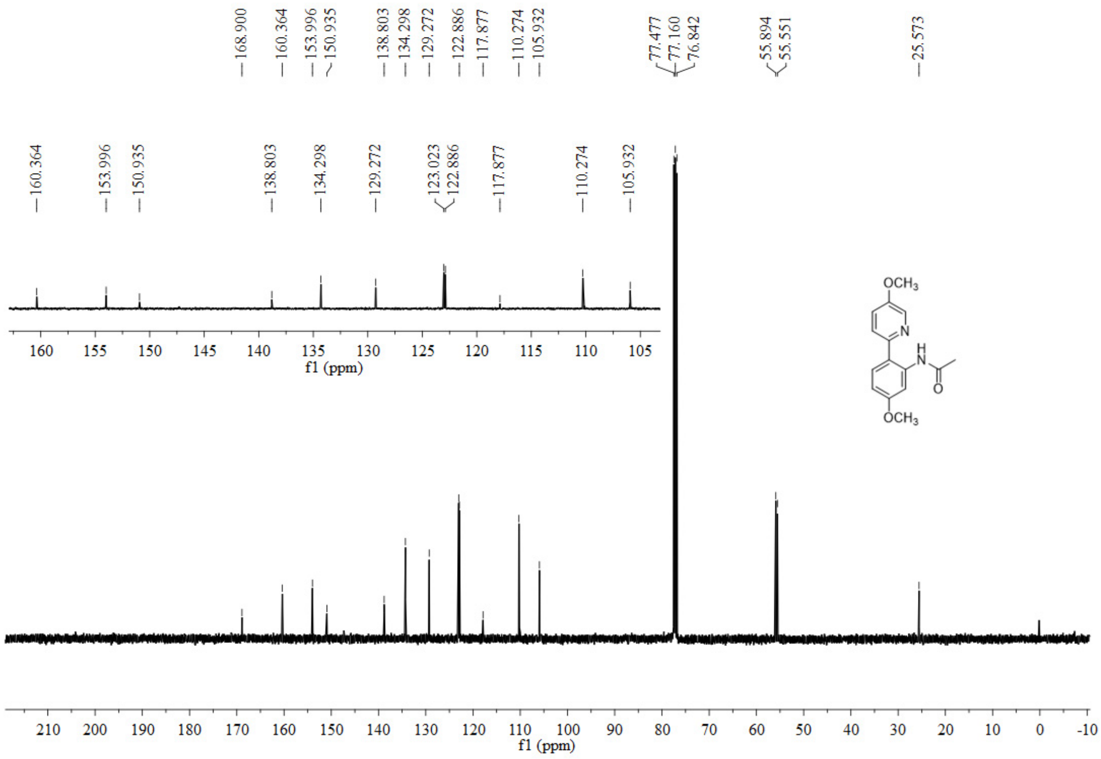

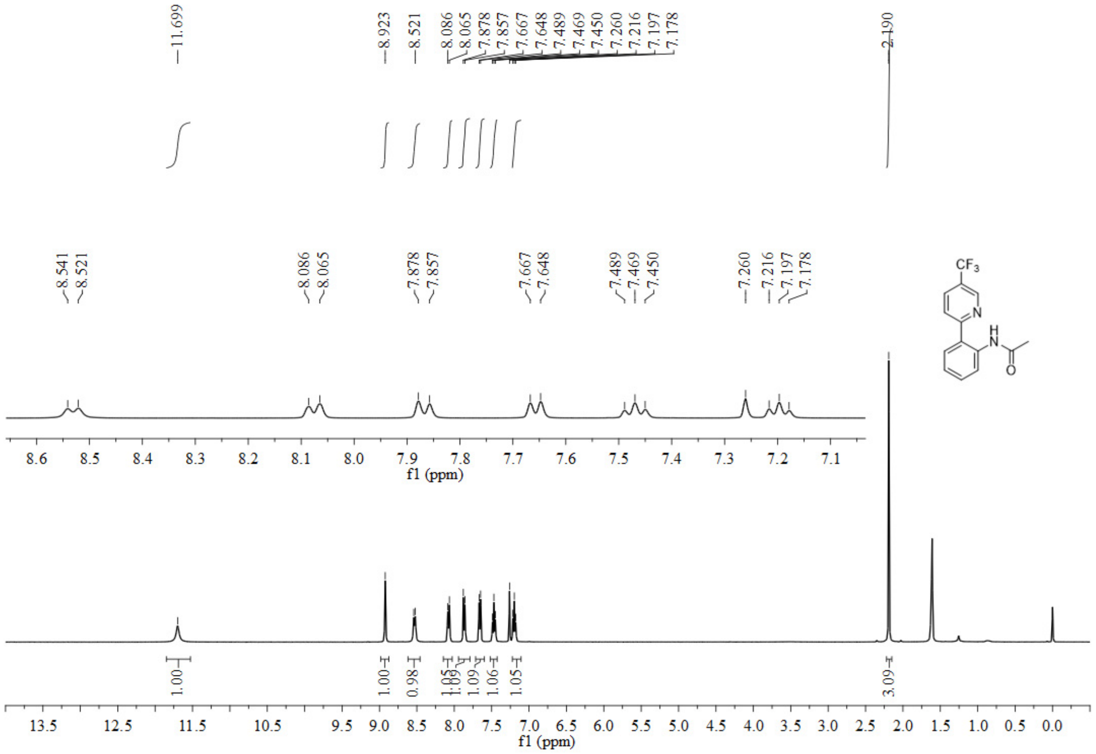

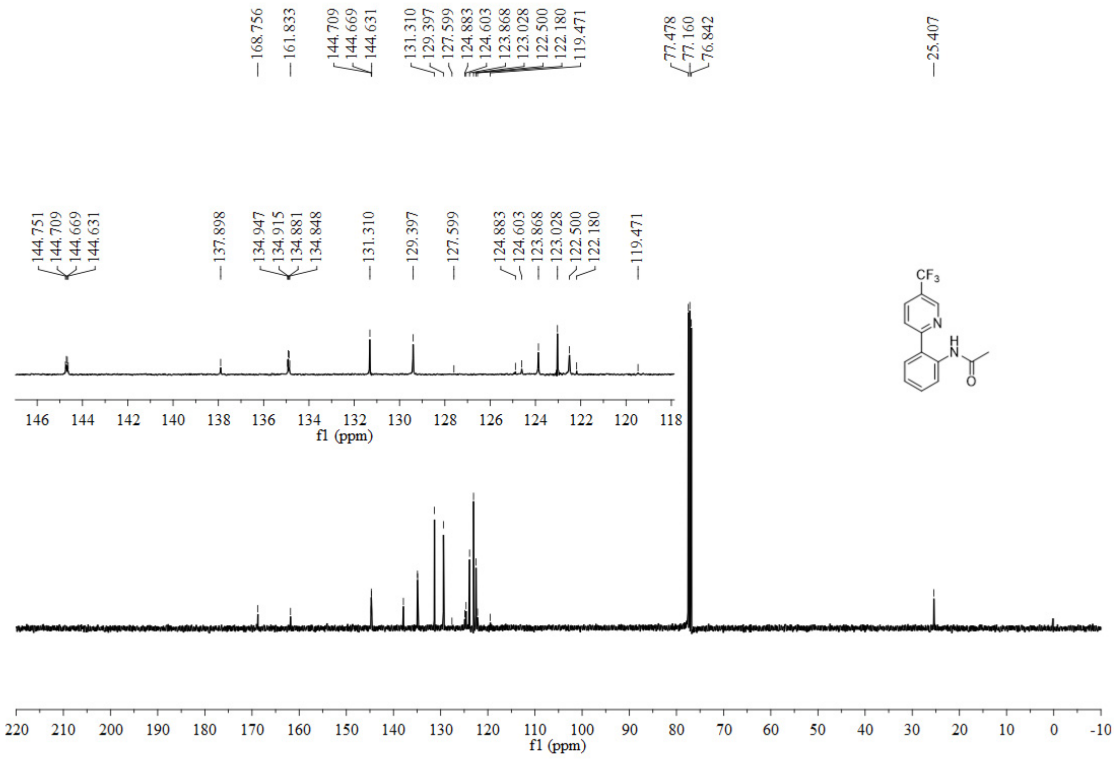

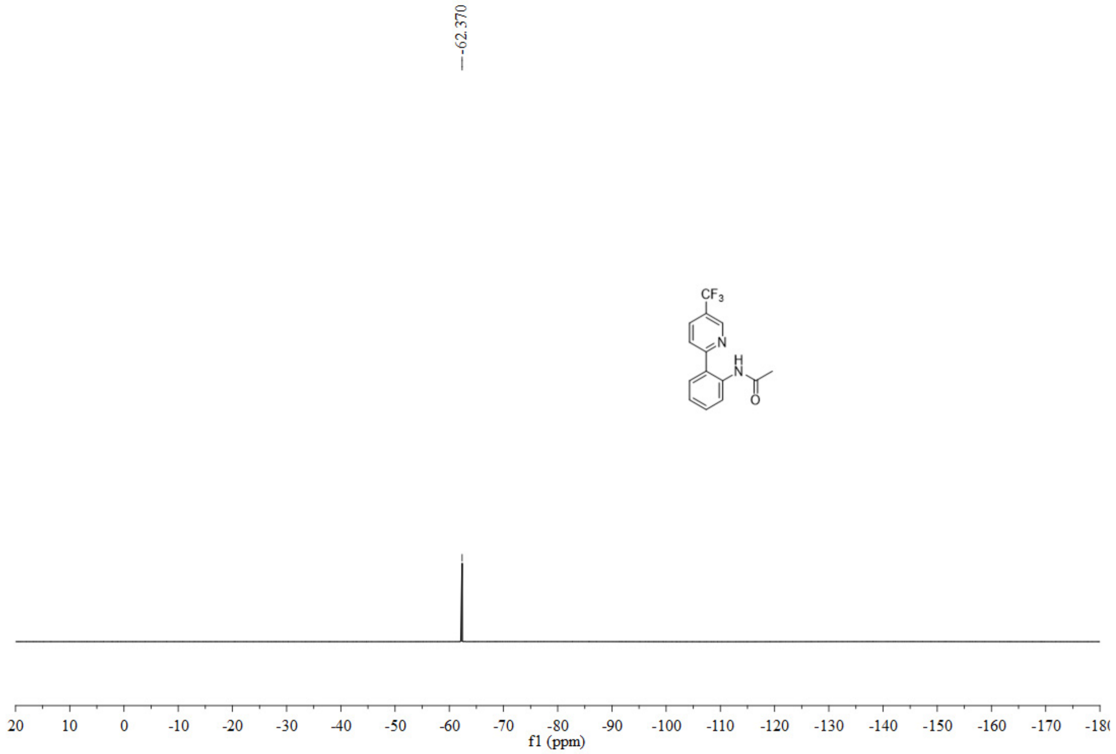
-62.370
CF3
N
H
N
O
20 10 0 -10 -20 -30 -40 -50 -60 -70 -80 -90 -100 -110 -120 -130 -140 -150 -160 -170
f1 (ppm)

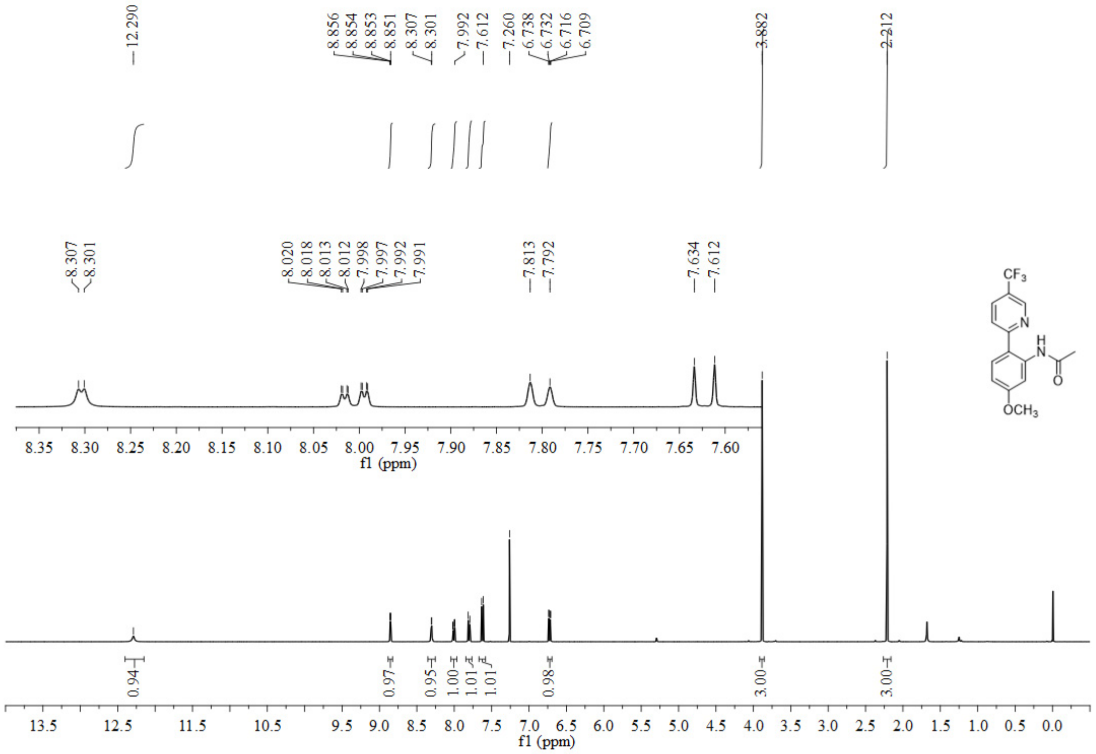
12.290
8.856
8.854
8.853
8.851
8.307
8.301
7.992
7.612
7.260
6.738
6.732
6.716
6.709
3.882
2.212
8.307
8.301
8.020
8.018
8.013
8.012
7.998
7.997
7.992
7.991
7.813
7.792
7.634
7.612
CF3
N
H
N
O
OCH3
8.35 8.30 8.25 8.20 8.15 8.10 8.05 8.00 7.95 7.90 7.85 7.80 7.75 7.70 7.65 7.60
f1 (ppm)
0.94
0.97
0.95
1.00
1.01
1.01
0.98
3.00
3.00
13.5 12.5 11.5 10.5 9.5 9.0 8.5 8.0 7.5 7.0 6.5 6.0 5.5 5.0 4.5 4.0 3.5 3.0 2.5 2.0 1.5 1.0 0.5 0.0
f1 (ppm)

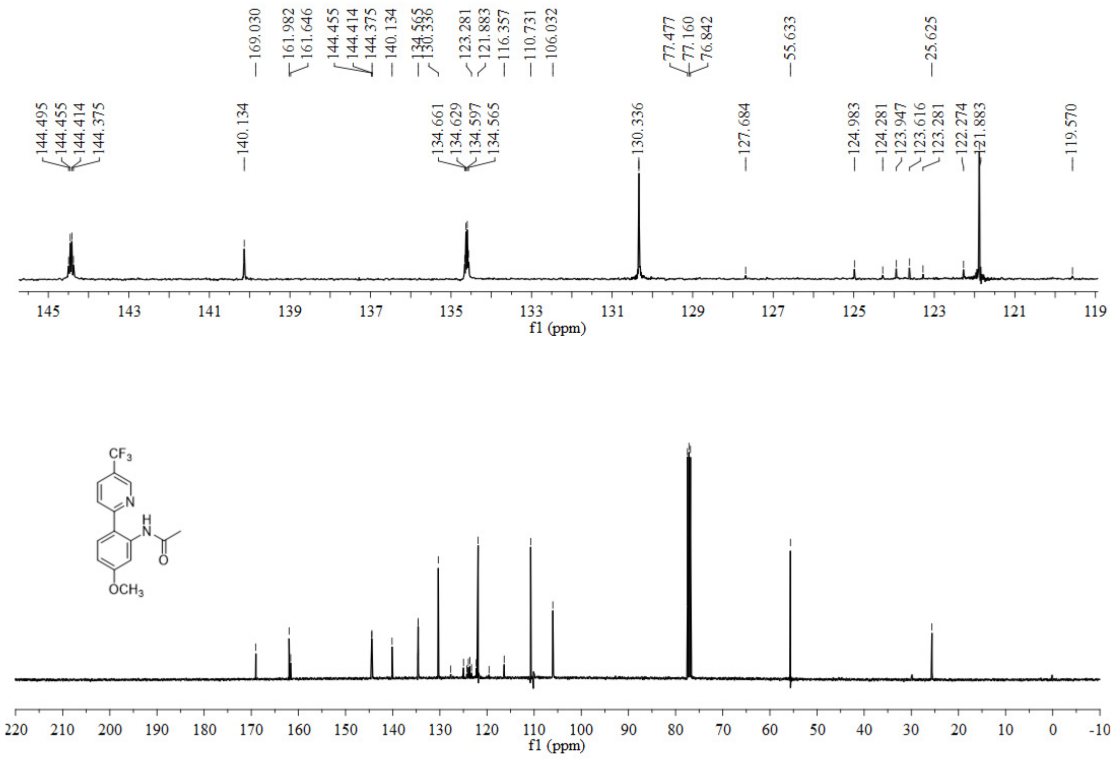
169.030
161.982
161.646
144.455
144.414
144.375
140.134
134.565
130.336
123.281
121.883
116.357
110.731
106.032
77.477
77.160
76.842
55.633
25.625
144.495
144.455
144.414
144.375
140.134
134.661
134.629
134.597
134.565
130.336
127.684
124.983
124.281
123.947
123.616
123.281
122.274
121.883
119.570
145 143 141 139 137 135 133 131 129 127 125 123 121 119
f1 (ppm)
CF3
N
H
N
O
OCH3
220 210 200 190 180 170 160 150 140 130 120 110 100 90 80 70 60 50 40 30 20 10 0 -10
f1 (ppm)

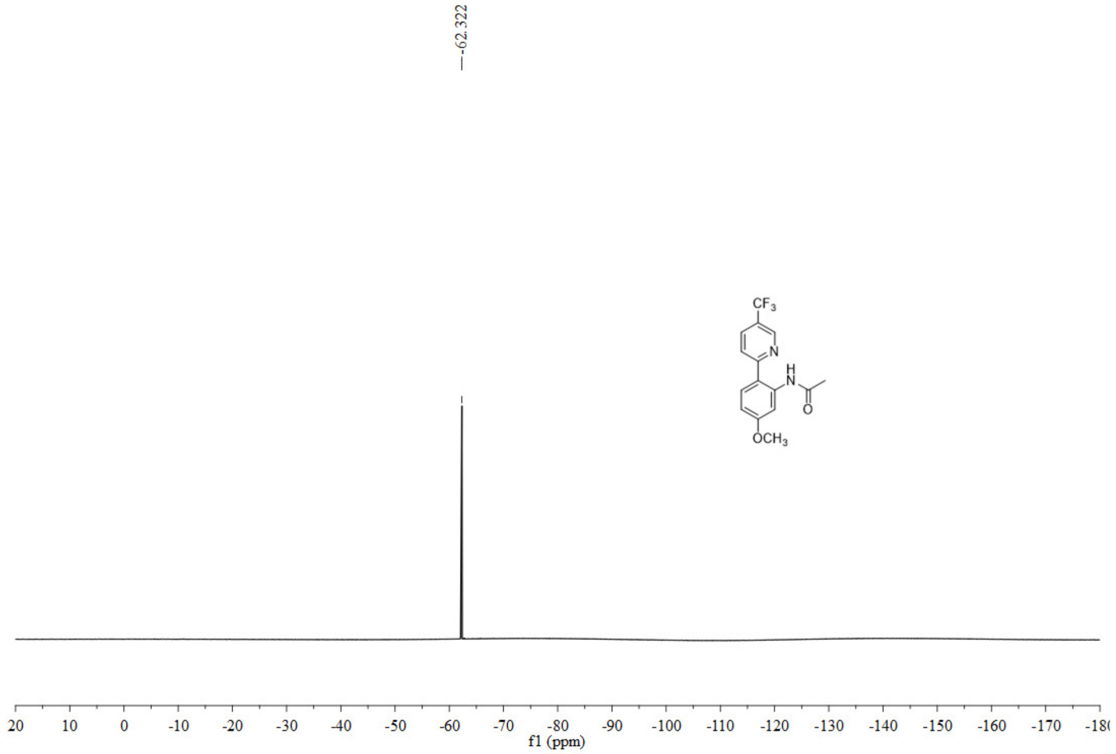

-62.322
CF3
N
H
O
OCH3
20 10 0 -10 -20 -30 -40 -50 -60 -70 -80 -90 -100 -110 -120 -130 -140 -150 -160 -170 -180
f1 (ppm)

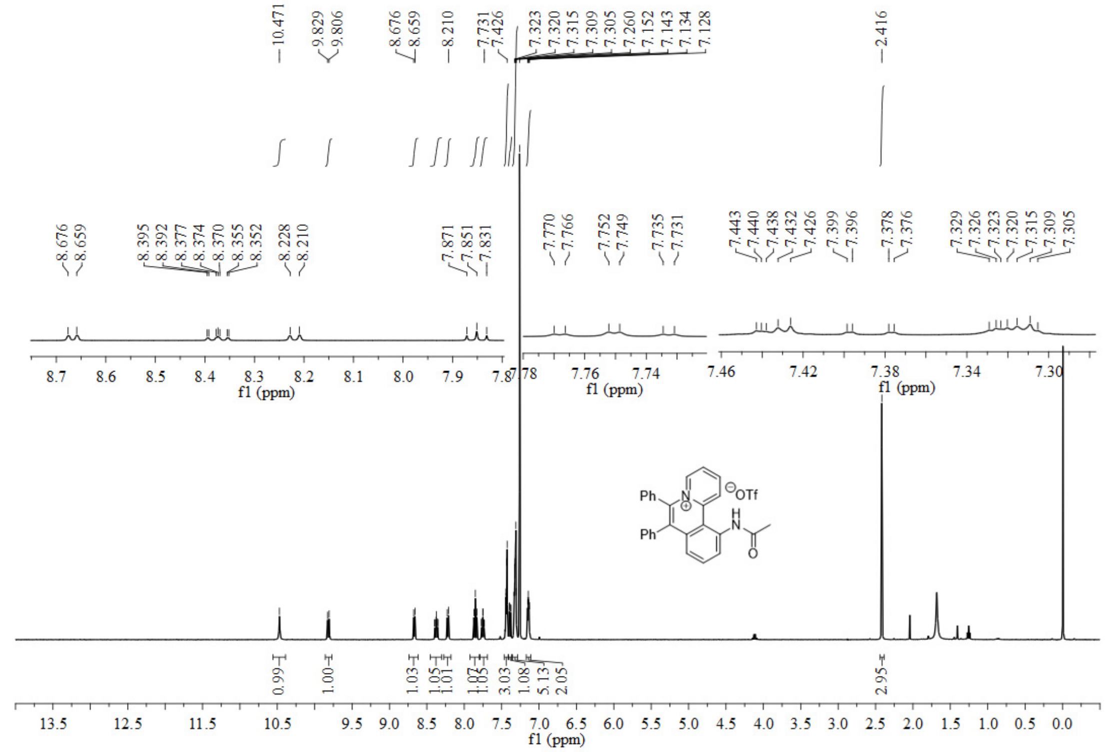
10.471
9.829
9.806
8.676
8.659
8.210
7.731
7.426
7.323
7.320
7.315
7.309
7.305
7.260
7.152
7.143
7.134
7.128
2.416
Ph
Ph
OTf
H
N
O
f1 (ppm)

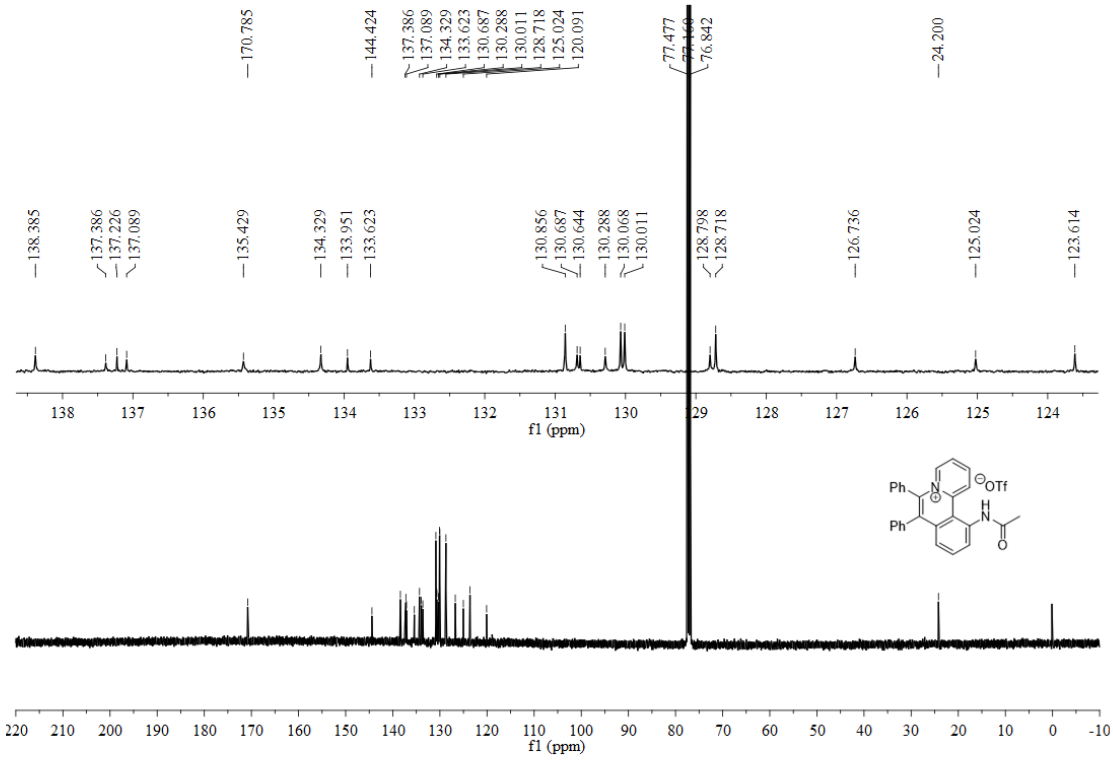
170.785
144.424
137.386
137.089
134.329
133.623
130.687
130.288
130.011
128.718
125.024
120.091
77.477
76.842
24.200
138.385
137.386
137.226
137.089
135.429
134.329
133.951
133.623
130.856
130.687
130.644
130.288
130.068
130.011
128.798
128.718
126.736
125.024
123.614
Ph
Ph
OTf
H
N
O
f1 (ppm)

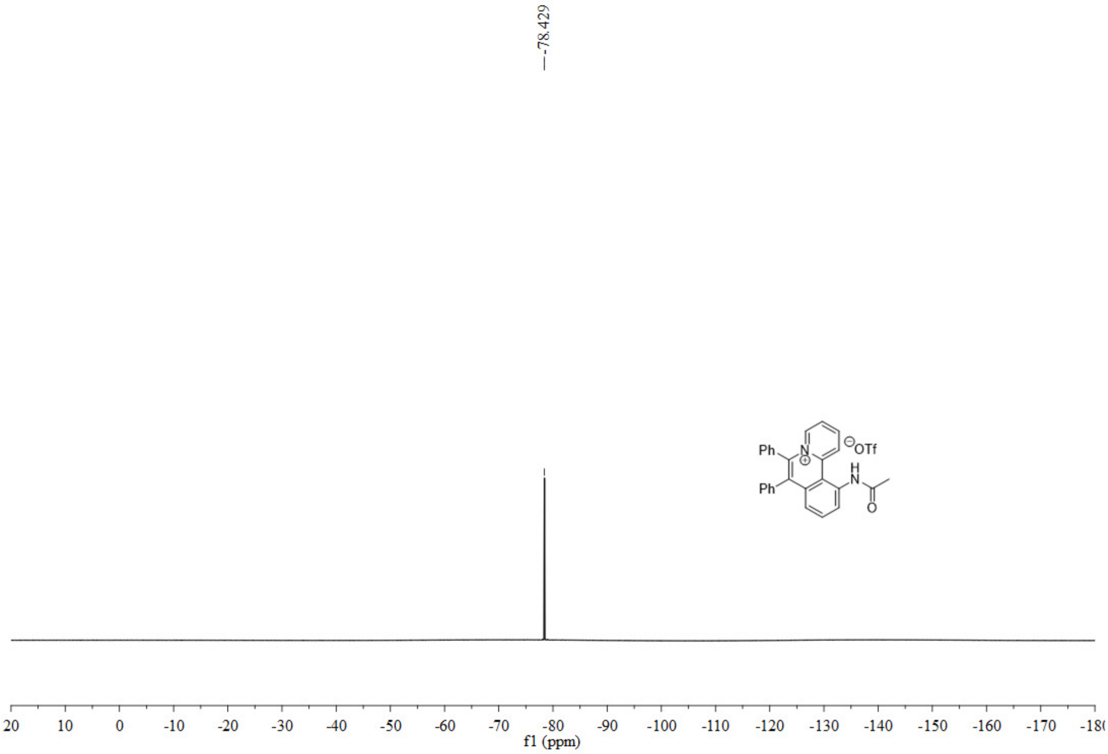

—78.429
Ph
Ph
N
OTf
H
N
O
20 10 0 -10 -20 -30 -40 -50 -60 -70 -80 -90 -100 -110 -120 -130 -140 -150 -160 -170
f1 (ppm)

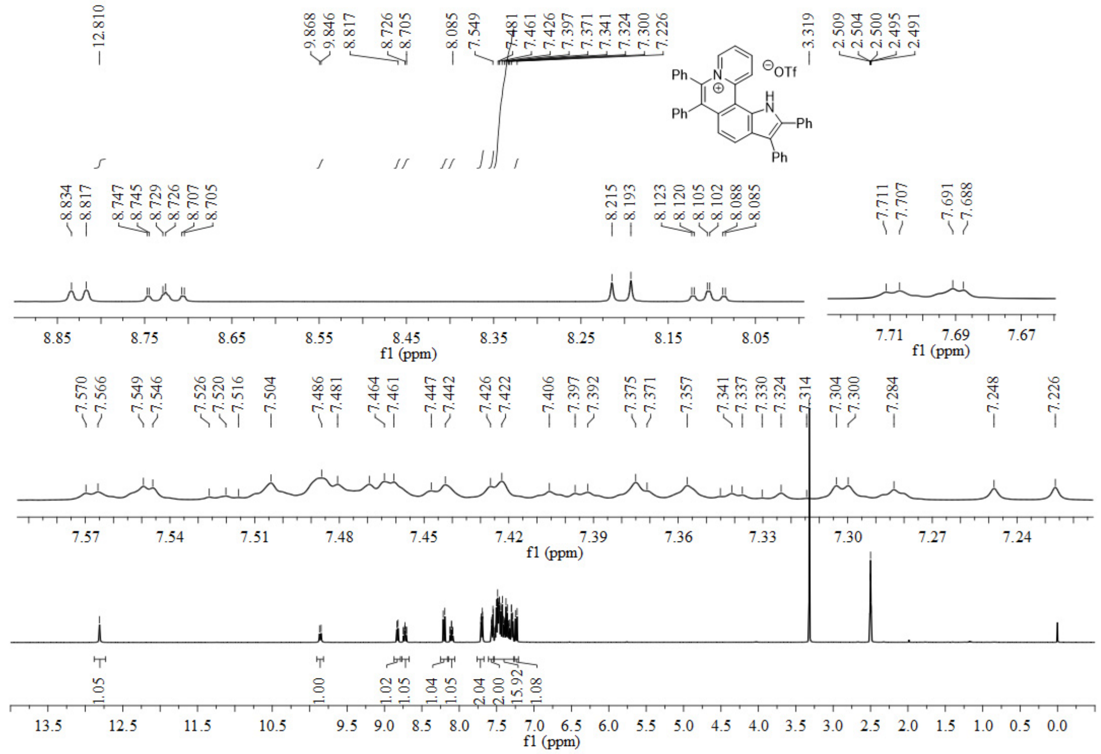

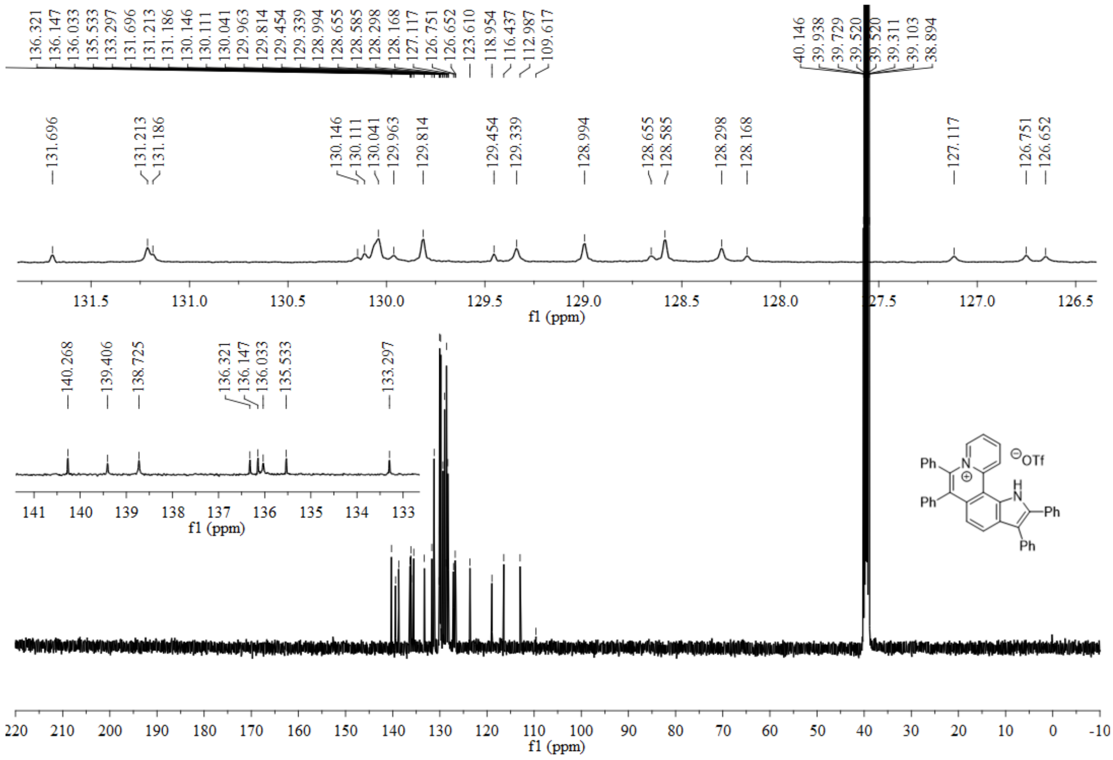

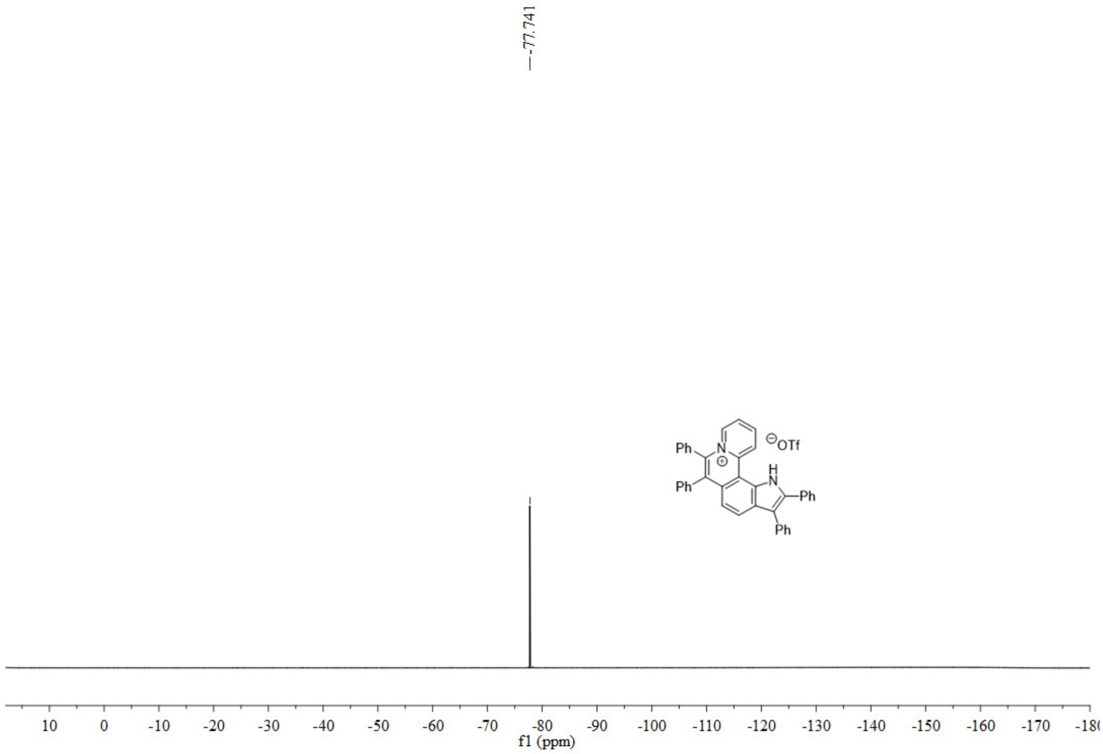

—-77.741
Ph
Ph
N
OTf
H
N
Ph
Ph
10 0 -10 -20 -30 -40 -50 -60 -70 -80 -90 -100 -110 -120 -130 -140 -150 -160 -170
f1 (ppm)

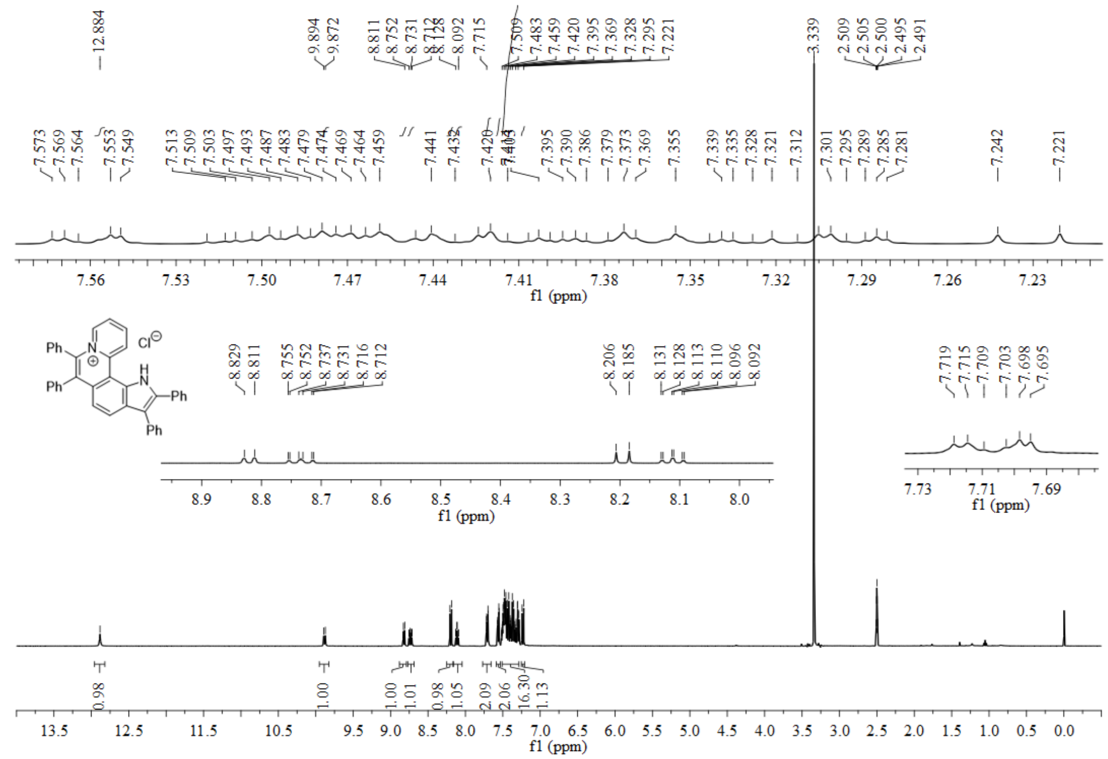
12.884
9.894
9.872
8.811
8.752
8.731
8.712
8.128
8.092
7.715
7.509
7.483
7.459
7.420
7.395
7.369
7.328
7.295
7.221
3.339
2.509
2.505
2.500
2.495
2.491
f1 (ppm)
Ph
Cl⊖
NH
Ph
Ph
Ph
0.98
1.00
1.00
1.01
0.98
1.05
2.09
2.06
16.30
1.13

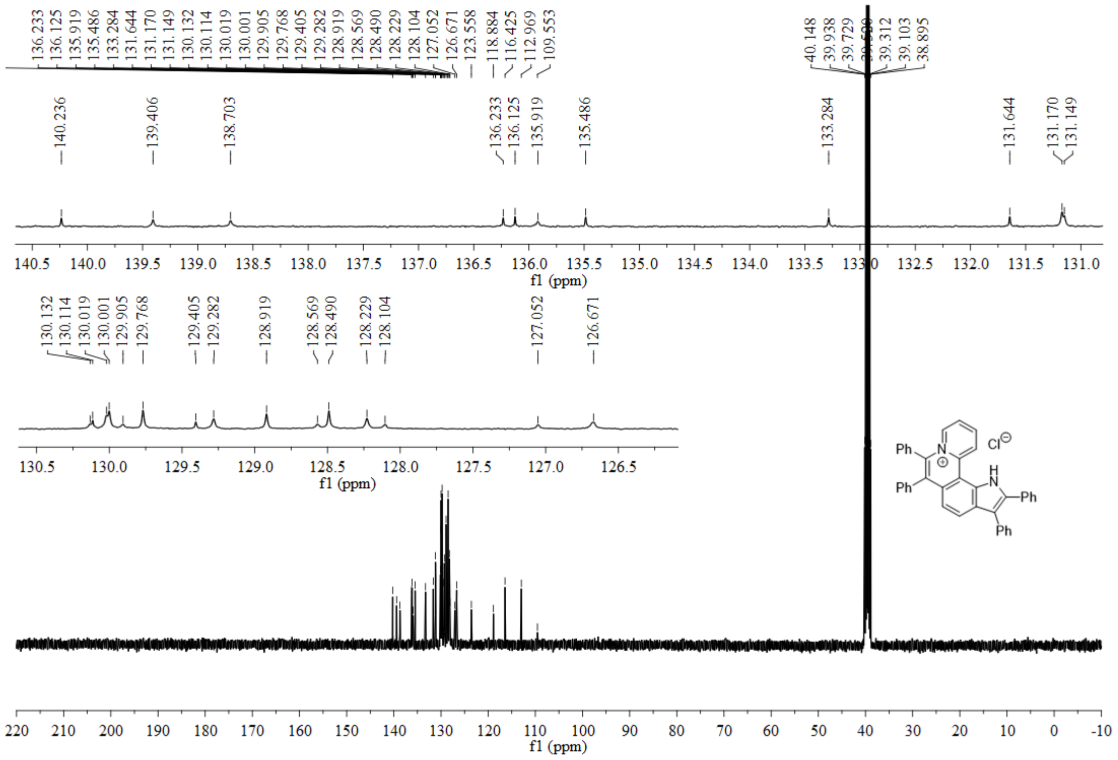
136.233
136.125
135.919
135.486
133.284
131.644
131.170
131.149
130.132
130.114
130.019
130.001
129.905
129.768
129.405
129.282
128.919
128.569
128.490
128.229
128.104
127.052
126.671
123.558
118.884
116.425
112.969
109.553
40.148
39.938
39.729
39.312
39.103
38.895
140.236
139.406
138.703
f1 (ppm)
Ph
Cl⊖
NH
Ph
Ph
Ph

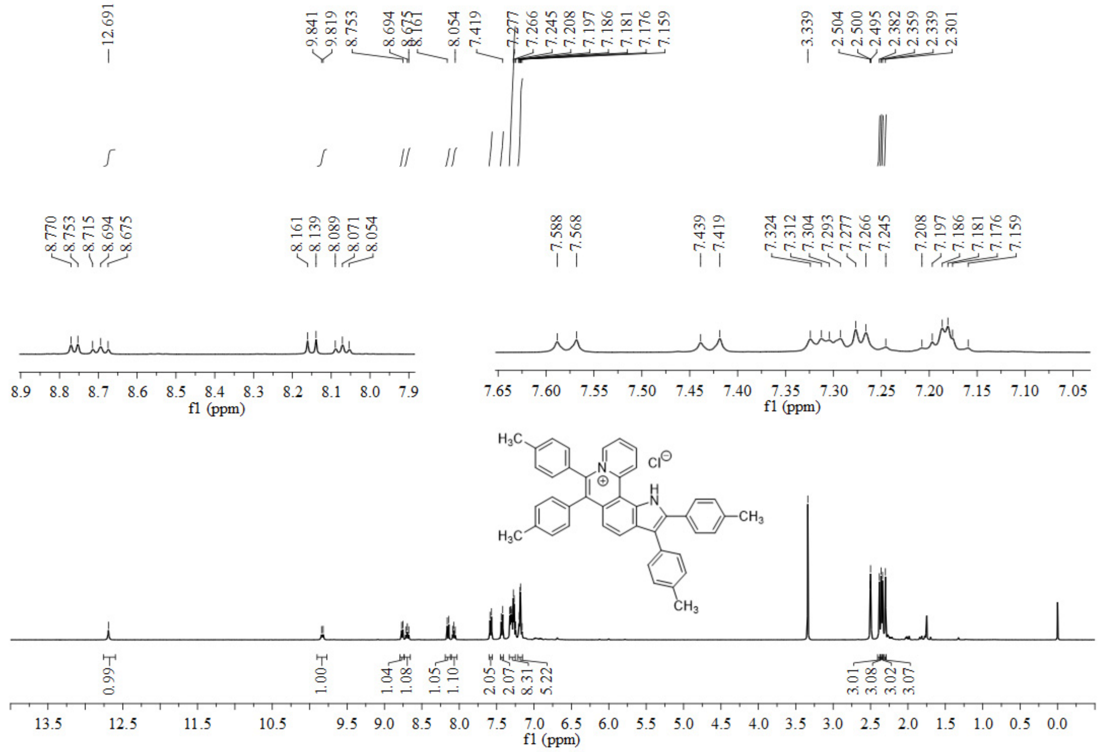
12.691
9.841
9.819
8.753
8.694
8.675
8.161
8.054
7.419
7.277
7.266
7.245
7.208
7.197
7.186
7.181
7.176
7.159
3.339
2.504
2.500
2.495
2.382
2.359
2.339
2.301
8.770
8.753
8.715
8.694
8.675
8.161
8.139
8.089
8.071
8.054
7.588
7.568
7.439
7.419
7.324
7.312
7.304
7.293
7.277
7.266
7.245
7.208
7.197
7.186
7.181
7.176
7.159
f1 (ppm)
H3C
CH3
Cl⊖
NH
0.99
1.00
1.04
1.08
1.05
1.10
2.05
2.07
8.31
5.22
3.01
3.08
3.02
3.07
f1 (ppm)

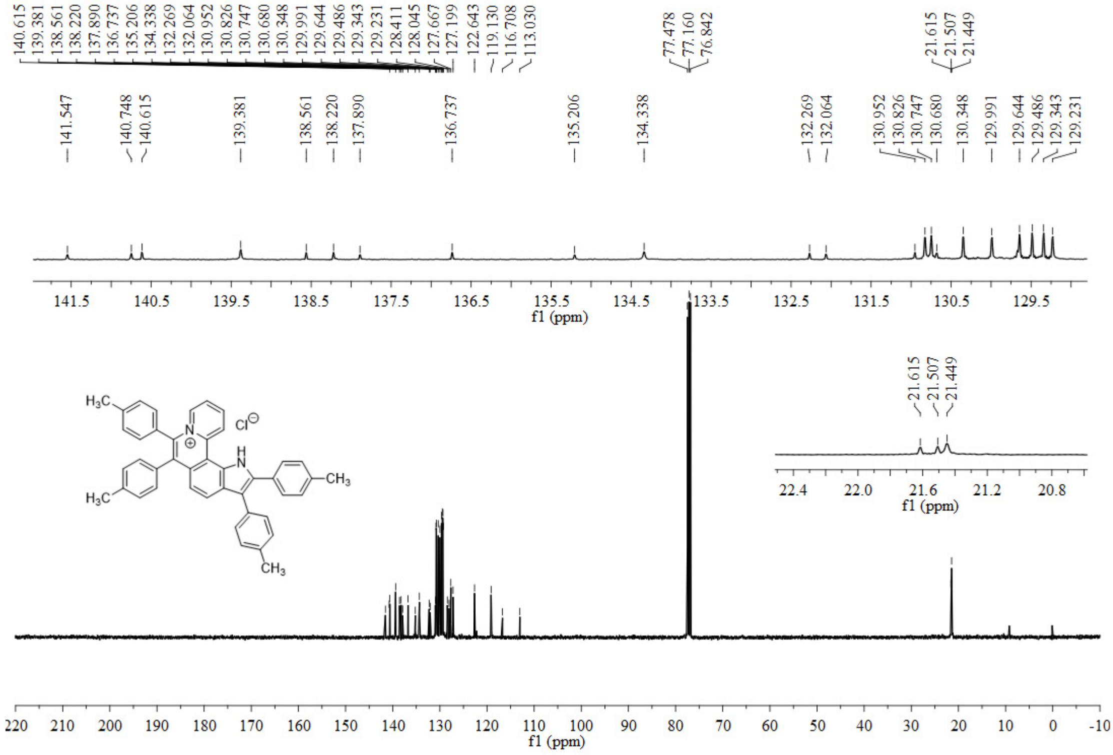
140.615
139.381
138.561
138.220
137.890
136.737
135.206
134.338
132.269
132.064
130.952
130.826
130.747
130.680
130.348
129.991
129.644
129.486
129.343
129.231
128.411
128.045
127.667
127.199
122.643
119.130
116.708
113.030
77.478
77.160
76.842
21.615
21.507
21.449
141.547
140.748
140.615
f1 (ppm)
H3C
CH3
Cl⊖
NH
f1 (ppm)
f1 (ppm)

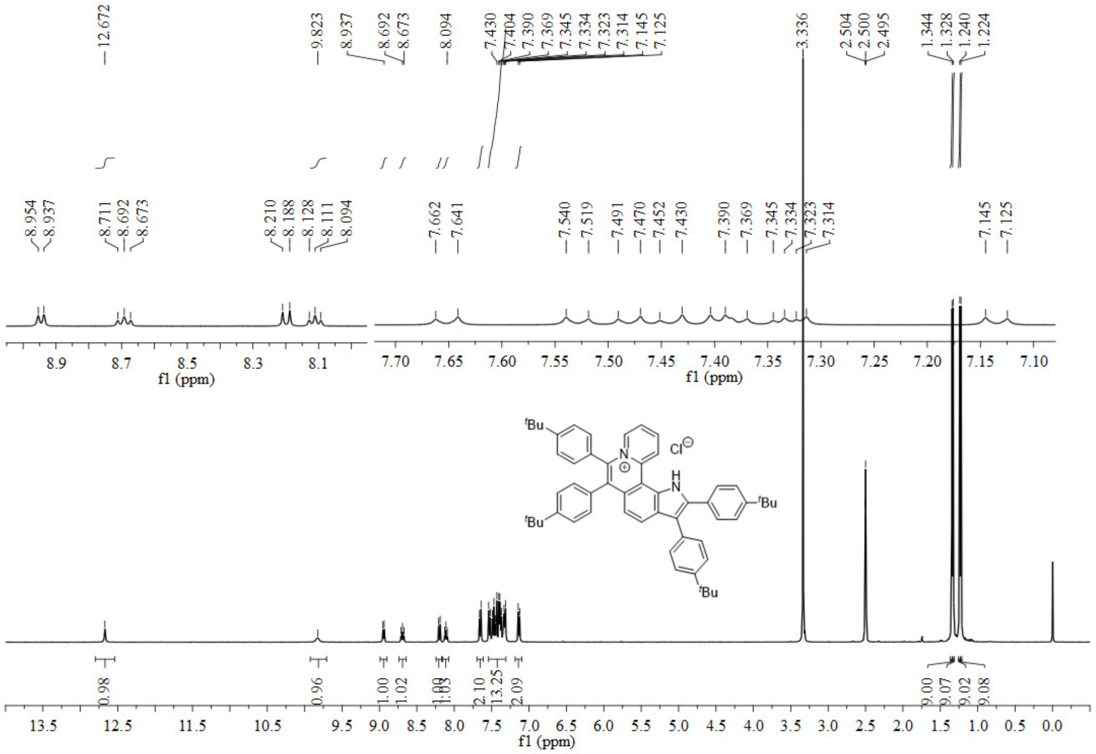
12.672
9.823
8.937
8.692
8.673
8.094
7.430
7.404
7.390
7.369
7.345
7.334
7.323
7.314
7.145
7.125
3.336
2.504
2.500
2.495
1.344
1.328
1.240
1.224
8.954
8.937
8.711
8.692
8.673
8.210
8.188
8.128
8.111
8.094
7.662
7.641
7.540
7.519
7.491
7.470
7.452
7.430
7.390
7.369
7.345
7.334
7.323
7.314
7.145
7.125
f1 (ppm)
tBu
Cl⊖
N
H
0.98
0.96
1.00
1.02
1.00
1.03
2.10
13.25
2.09
9.00
9.07
9.02
9.08
f1 (ppm)

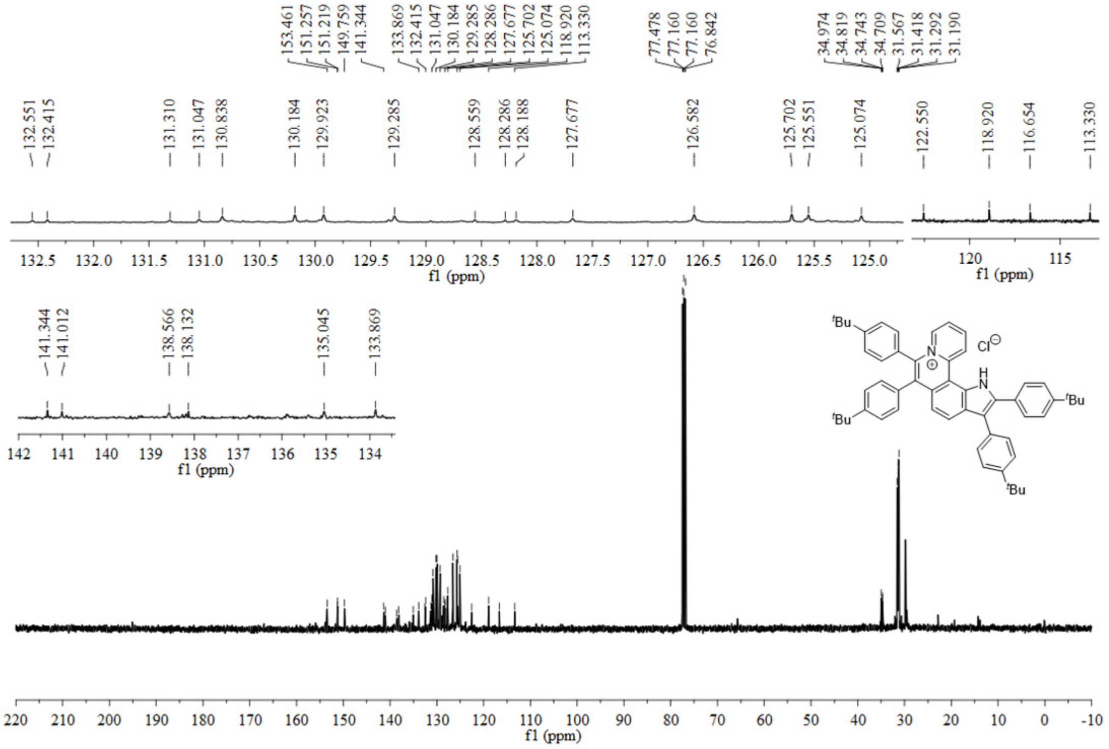
153.461
151.257
151.219
149.759
141.344
133.869
132.415
131.047
130.184
129.285
128.286
127.677
125.702
125.074
118.920
113.330
77.478
77.160
77.160
76.842
34.974
34.819
34.743
34.709
31.567
31.418
31.292
31.190
132.551
132.415
131.310
131.047
130.838
130.184
129.923
129.285
128.559
128.286
128.188
127.677
126.582
125.702
125.551
125.074
122.550
118.920
116.654
113.330
f1 (ppm)
141.344
141.012
138.566
138.132
135.045
133.869
f1 (ppm)
tBu
Cl⊖
N
H
f1 (ppm)

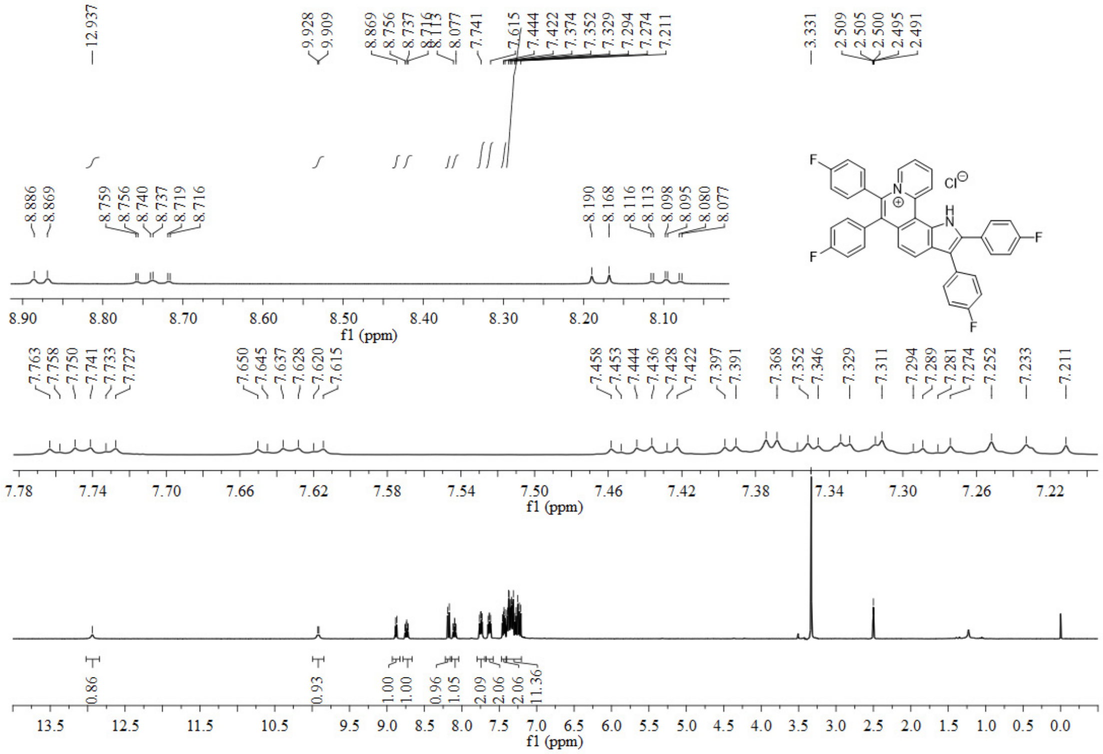
12.937
9.928
9.909
8.869
8.756
8.737
8.716
8.113
8.077
7.741
7.615
7.444
7.422
7.374
7.352
7.329
7.294
7.274
7.211
3.331
2.509
2.505
2.500
2.495
2.491
8.886
8.869
8.759
8.756
8.740
8.737
8.719
8.716
8.190
8.168
8.116
8.113
8.098
8.095
8.080
8.077
f1 (ppm)
7.763
7.758
7.750
7.741
7.733
7.727
7.650
7.645
7.637
7.628
7.620
7.615
7.458
7.453
7.444
7.436
7.428
7.422
7.397
7.391
7.368
7.352
7.346
7.329
7.311
7.294
7.289
7.281
7.274
7.252
7.233
7.211
f1 (ppm)
0.86
0.93
1.00
1.00
0.96
1.05
2.09
2.06
2.06
11.36
f1 (ppm)
Cl⊖
N
⊕
H
N
F
F
F
F

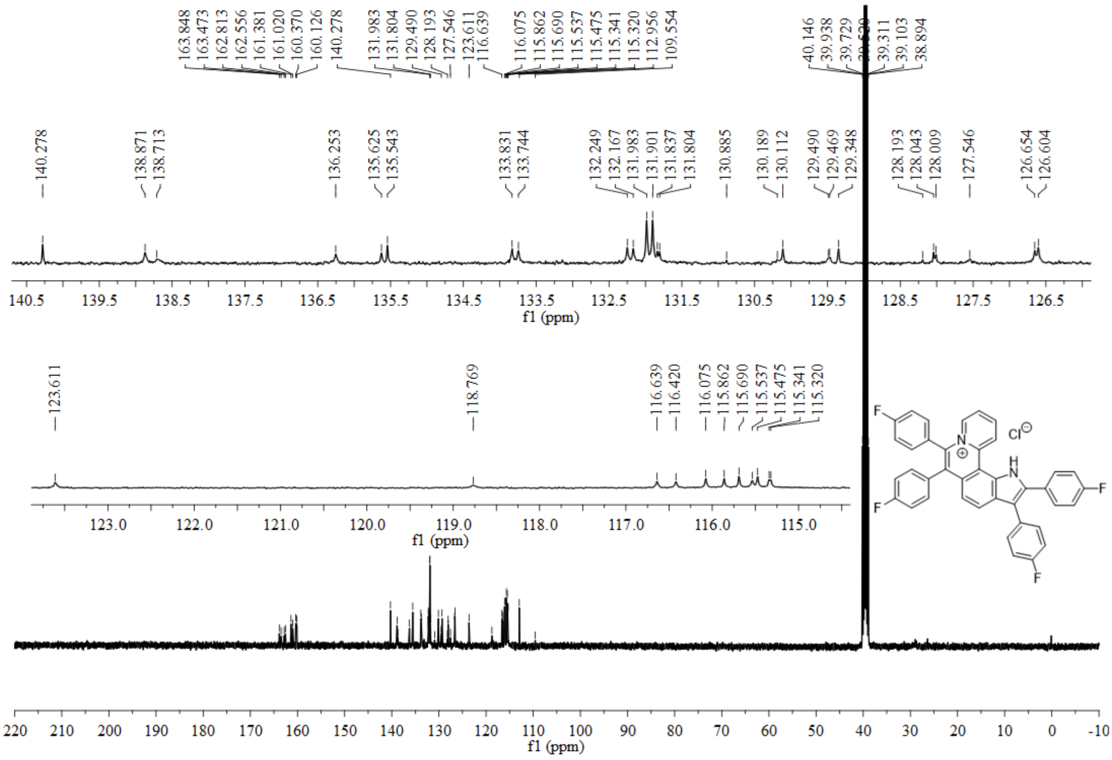
163.848
163.473
162.813
162.556
161.381
161.020
160.370
160.126
140.278
131.983
131.804
129.490
128.193
127.546
123.611
116.639
116.075
115.862
115.690
115.537
115.475
115.341
115.320
112.956
109.554
40.146
39.938
39.729
39.311
39.103
38.894
140.278
138.871
138.713
136.253
135.625
135.543
133.831
133.744
132.249
132.167
131.983
131.901
131.837
131.804
130.885
130.189
130.112
129.490
129.469
129.348
128.193
128.043
128.009
127.546
126.654
126.604
f1 (ppm)
123.611
118.769
116.639
116.420
116.075
115.862
115.690
115.537
115.475
115.341
115.320
f1 (ppm)
f1 (ppm)
Cl⊖
N
⊕
H
N
F
F
F
F

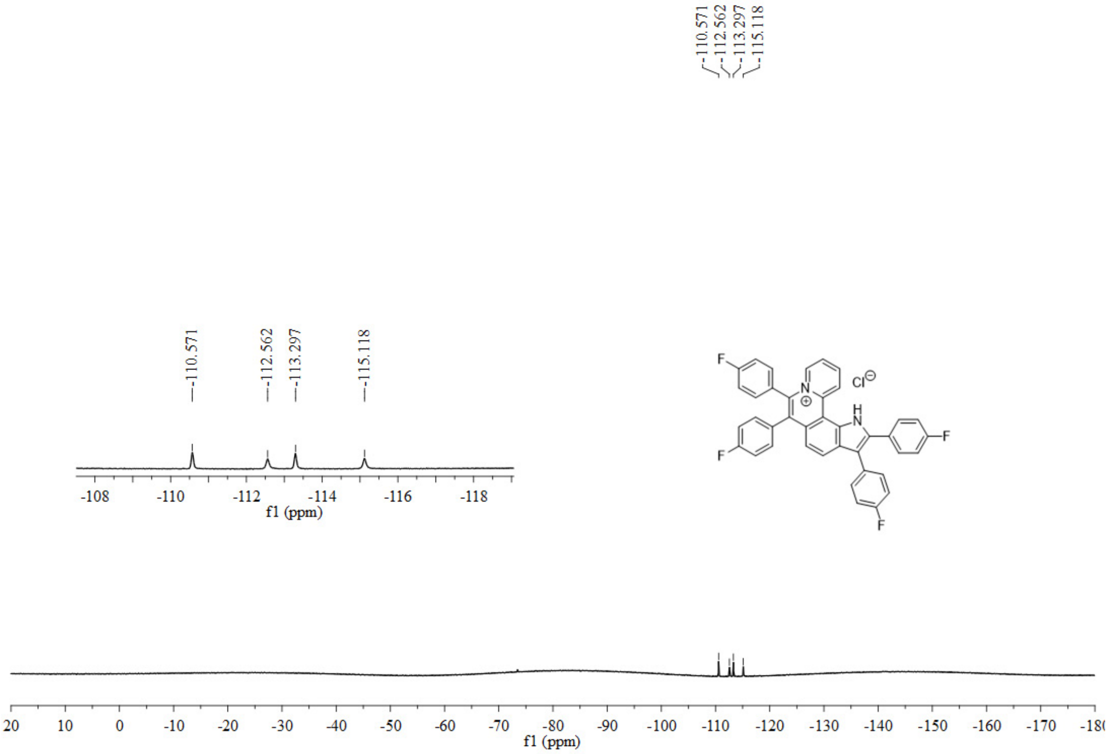
-110.571
-112.562
-113.297
-115.118
-108
-110
-112
-114
-116
-118
f1 (ppm)
F
F
F
F
N
⊕
Cl⊖
H
N
20
10
0
-10
-20
-30
-40
-50
-60
-70
-80
-90
-100
-110
-120
-130
-140
-150
-160
-170
f1 (ppm)

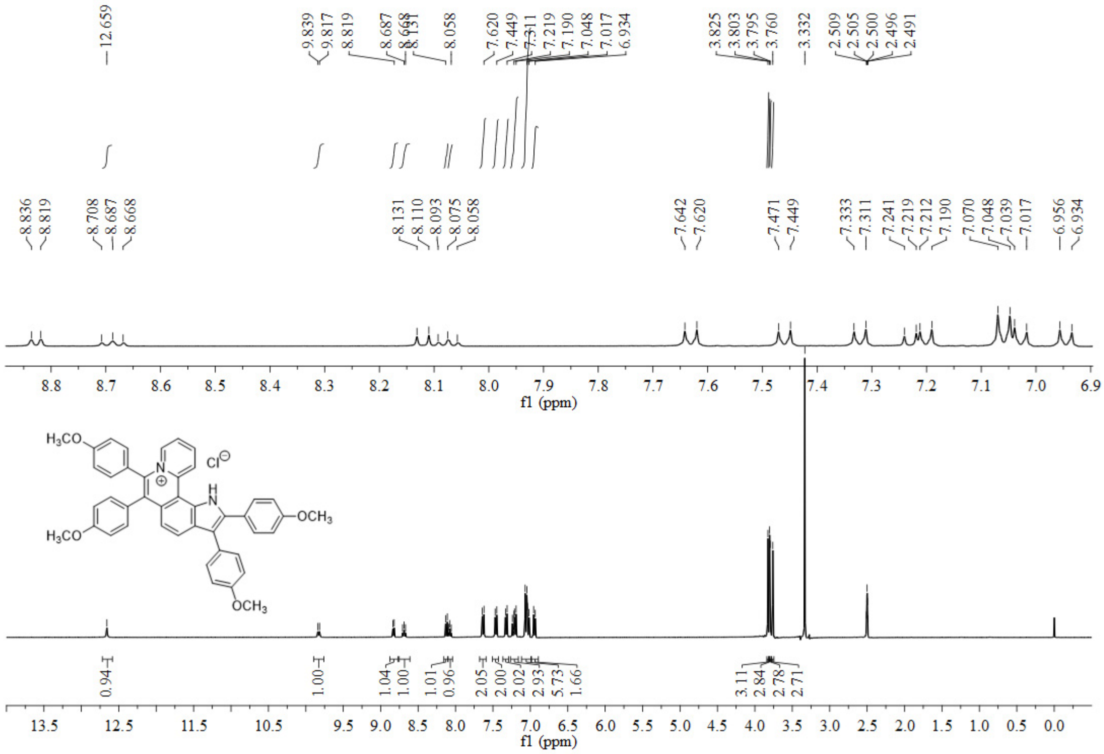
12.659
9.839 9.817 8.819 8.687 8.668 8.131 8.058 7.620 7.449 7.311 7.219 7.190 7.048 7.017 6.934
3.825 3.803 3.795 3.760 3.332 2.509 2.505 2.500 2.496 2.491
8.836 8.819 8.708 8.687 8.668 8.131 8.110 8.093 8.075 8.058 7.642 7.620 7.471 7.449 7.333 7.311 7.241 7.219 7.212 7.190 7.070 7.048 7.039 7.017 6.956 6.934
f1 (ppm)
H_3CO
OCH_3
N
H
Cl
0.94 1.00 1.04 1.00 1.01 0.96 2.05 2.00 2.02 2.93 5.73 1.66 3.11 2.84 2.78 2.71
f1 (ppm)

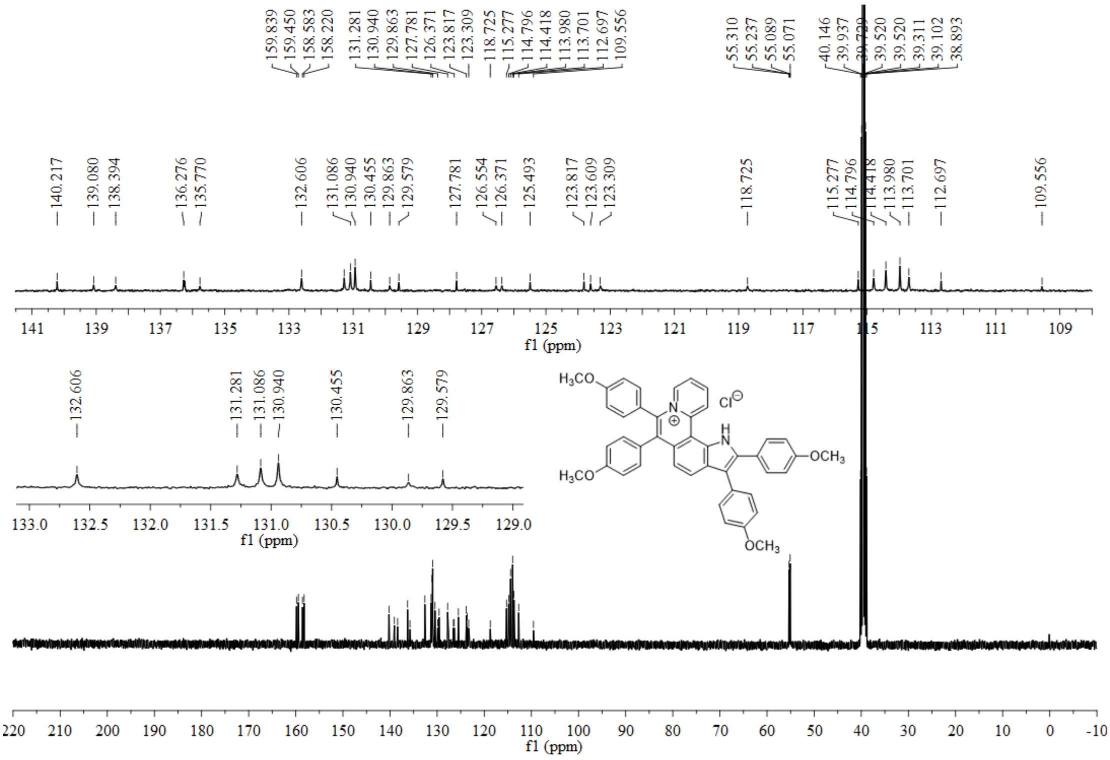
159.839 159.450 158.583 158.220 131.281 130.940 129.863 127.781 126.371 123.817 123.309 118.725 115.277 114.796 114.418 113.980 113.701 112.697 109.556
55.310 55.237 55.089 55.071 40.146 39.937 39.729 39.520 39.311 39.102 38.893
140.217 139.080 138.394 136.276 135.770 132.606 131.086 130.940 130.455 129.863 129.579 127.781 126.554 126.371 125.493 123.817 123.609 123.309 118.725 115.277 114.796 114.418 113.980 113.701 112.697 109.556
f1 (ppm)
132.606 131.281 131.086 130.940 130.455 129.863 129.579
f1 (ppm)
H_3CO
OCH_3
N
H
Cl
f1 (ppm)

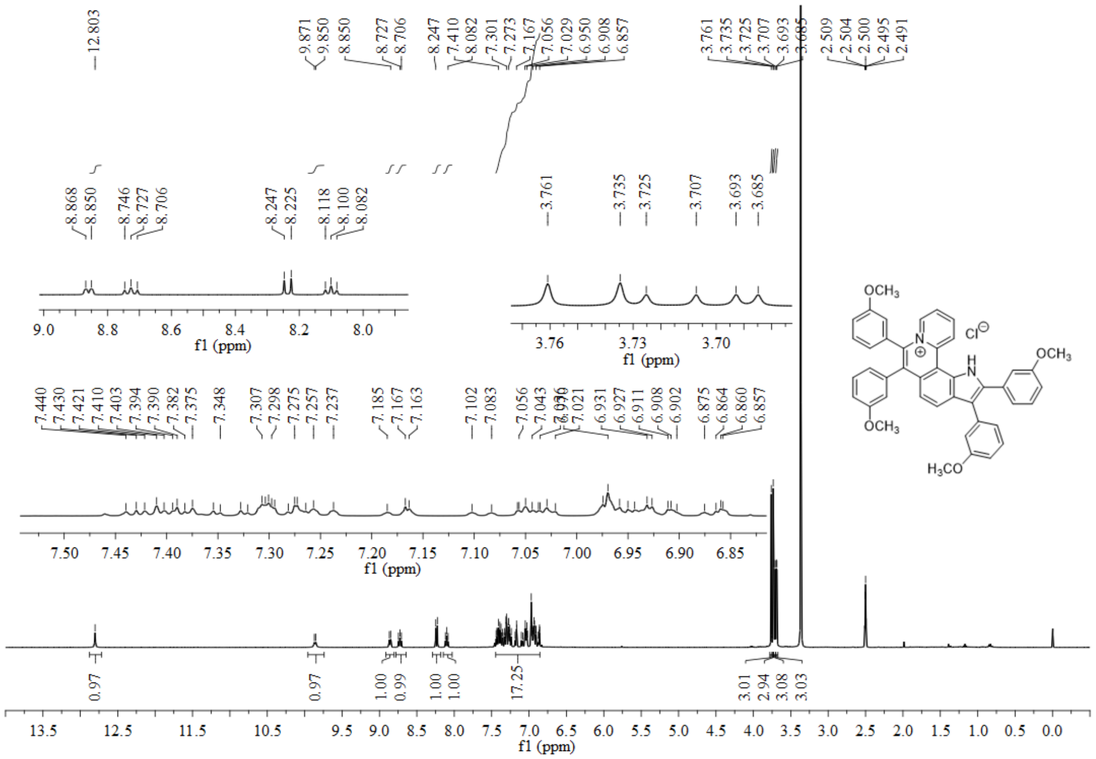
12.803
9.871
9.850
8.850
8.727
8.706
8.247
7.410
8.082
7.301
7.273
7.167
7.056
7.029
6.950
6.908
6.857
3.761
3.735
3.725
3.707
3.693
3.685
2.509
2.504
2.500
2.495
2.491
8.868
8.850
8.746
8.727
8.706
8.247
8.225
8.118
8.100
8.082
f1 (ppm)
3.761
3.735
3.725
3.707
3.693
3.685
f1 (ppm)
7.440
7.430
7.421
7.410
7.403
7.394
7.390
7.382
7.375
7.348
7.307
7.298
7.275
7.257
7.237
7.185
7.167
7.163
7.102
7.083
7.056
7.043
7.021
6.931
6.927
6.911
6.908
6.902
6.875
6.864
6.860
6.857
f1 (ppm)
OCH3
Cl⊖
OCH3
OCH3
H3CO
0.97
0.97
1.00
0.99
1.00
1.00
17.25
3.01
2.94
3.08
3.03
f1 (ppm)

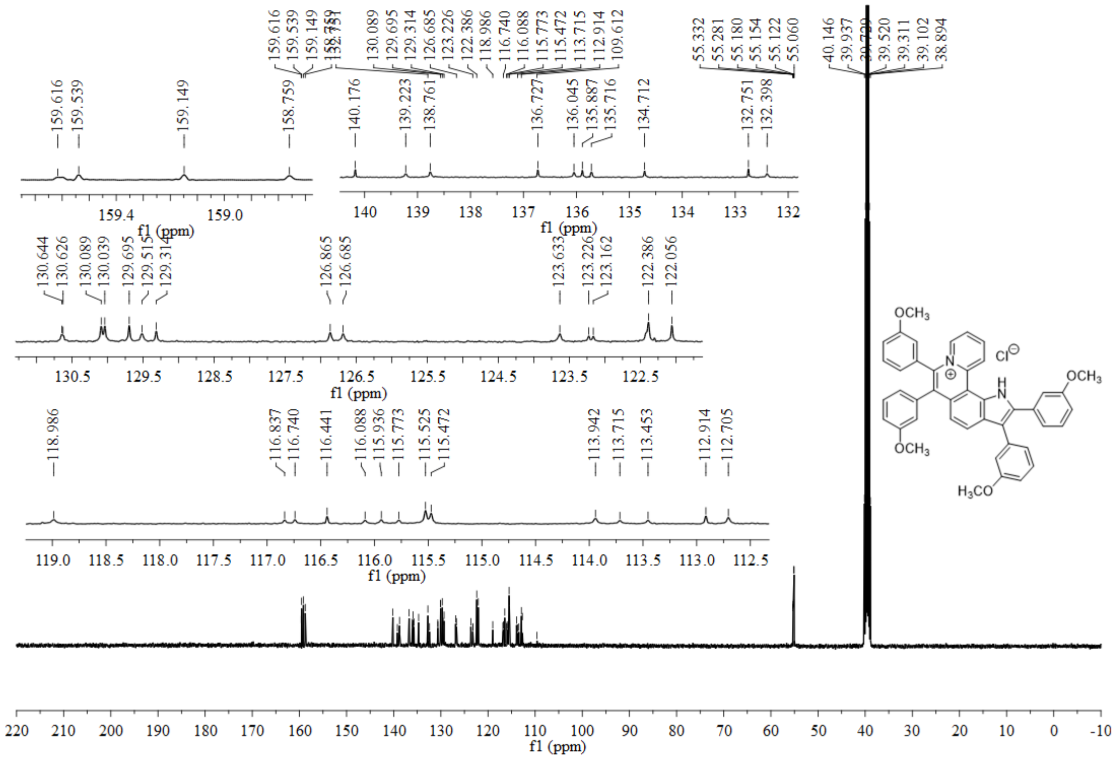
159.616
159.539
159.149
130.089
129.695
129.314
126.685
123.226
122.386
118.986
116.740
116.088
115.773
115.472
113.715
112.914
109.612
55.332
55.281
55.180
55.154
55.122
55.060
40.146
39.937
39.520
39.311
39.102
38.894
159.616
159.539
159.149
158.759
f1 (ppm)
140.176
139.223
138.761
136.727
136.045
135.887
135.716
134.712
132.751
132.398
f1 (ppm)
130.644
130.626
130.089
130.039
129.695
129.515
129.314
126.865
126.685
123.633
123.226
123.162
122.386
122.056
f1 (ppm)
118.986
116.837
116.740
116.441
116.088
115.936
115.773
115.525
115.472
113.942
113.715
113.453
112.914
112.705
f1 (ppm)
OCH3
Cl⊖
OCH3
OCH3
H3CO
f1 (ppm)

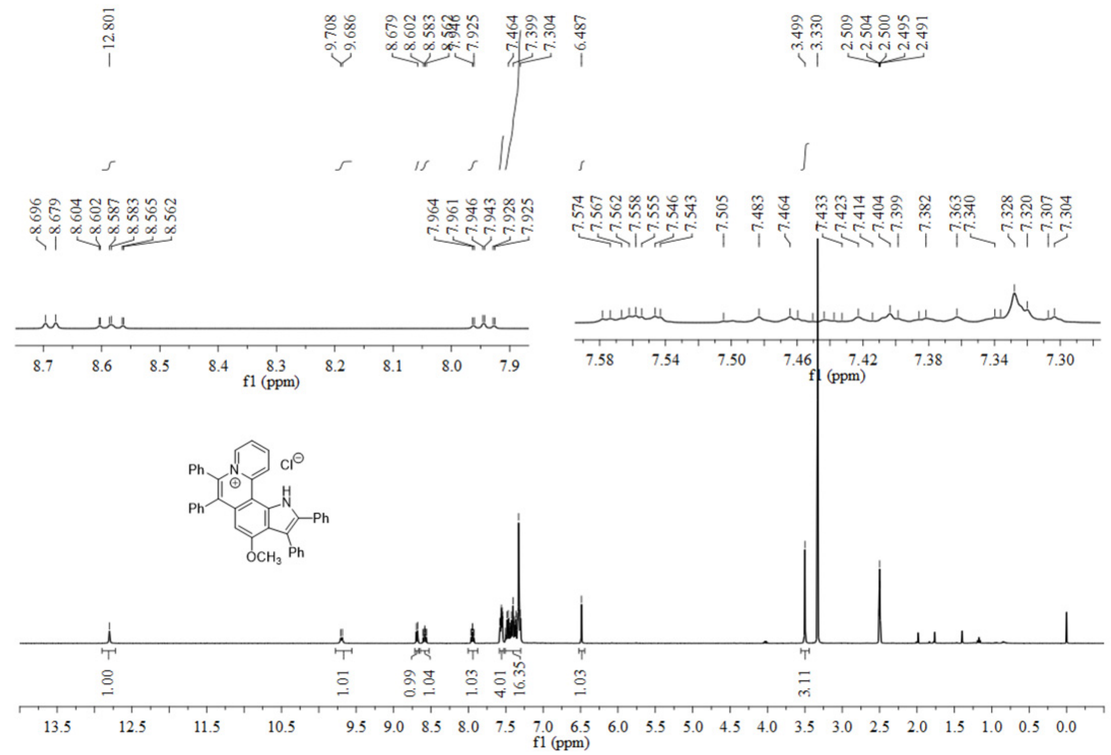
f1 (ppm)
Ph
Ph
Cl⊖
NH
Ph
OCH3
Ph

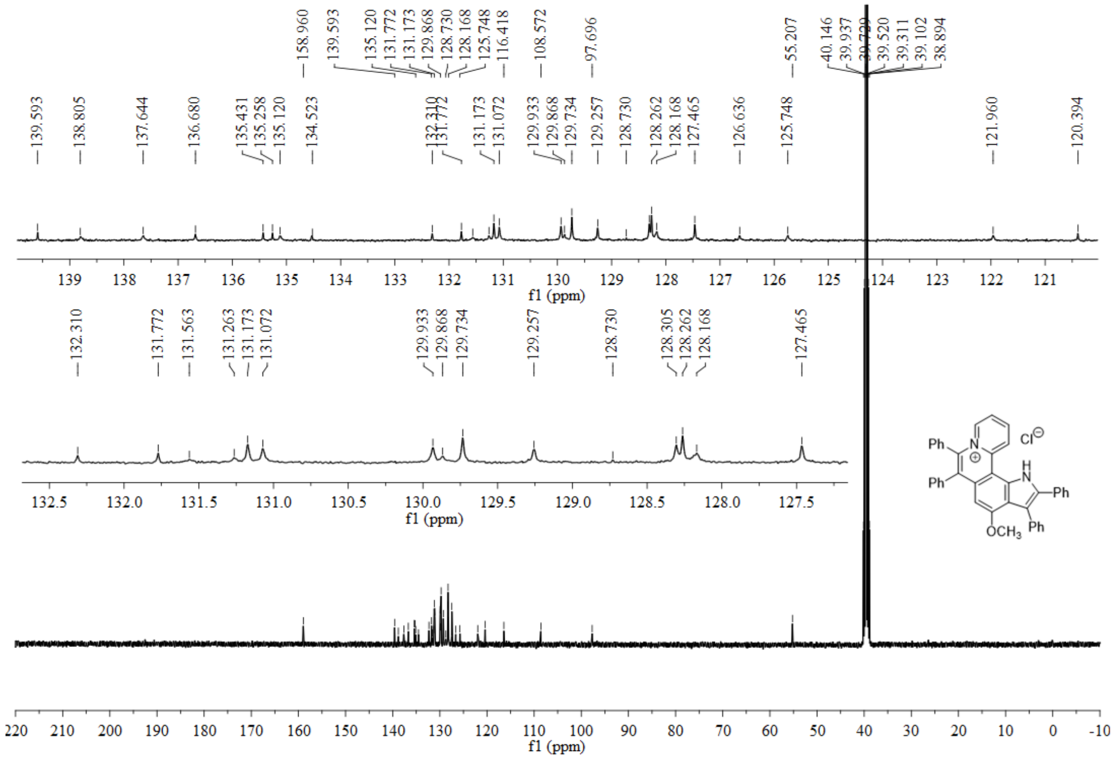
f1 (ppm)
Ph
Ph
Cl⊖
NH
Ph
OCH3
Ph

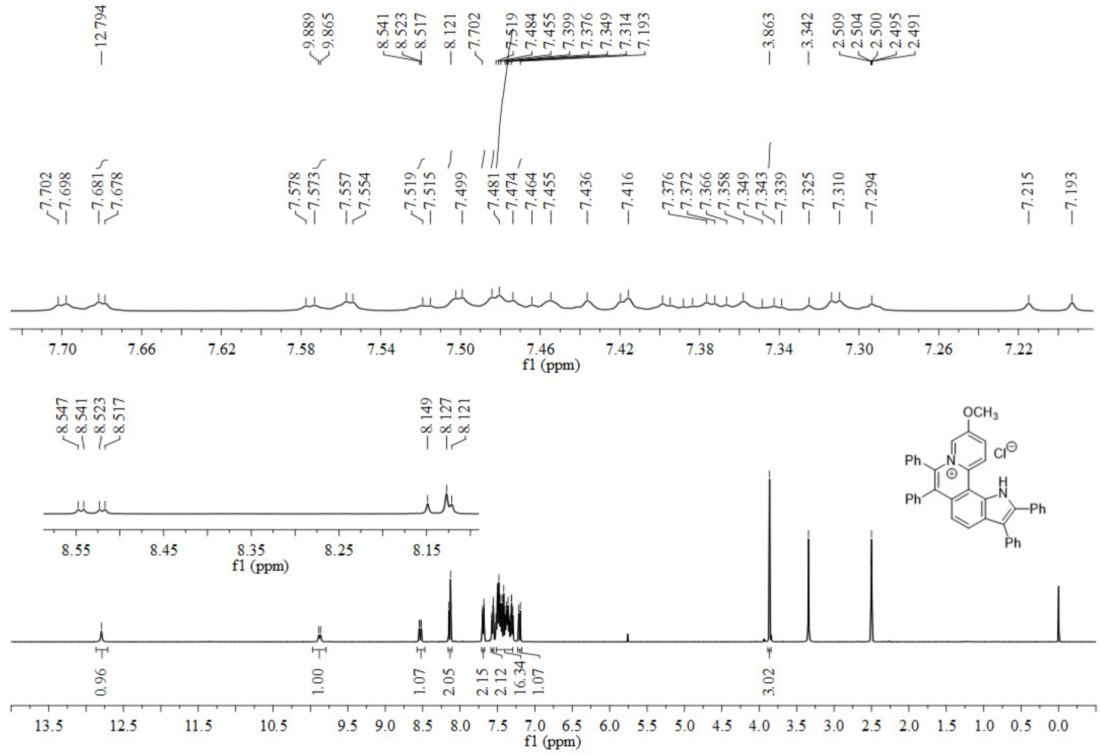
f1 (ppm)
OCH3
Cl
Ph
Ph
H
N
Ph
Ph

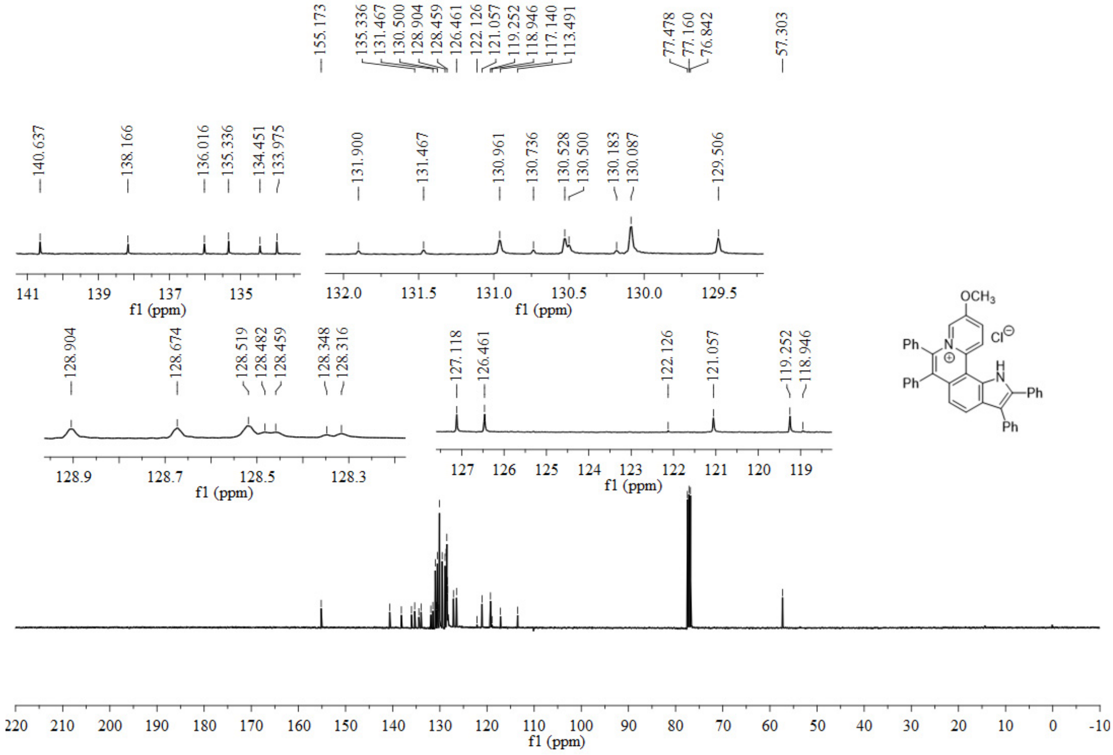
f1 (ppm)
OCH3
Cl
Ph
Ph
H
N
Ph
Ph

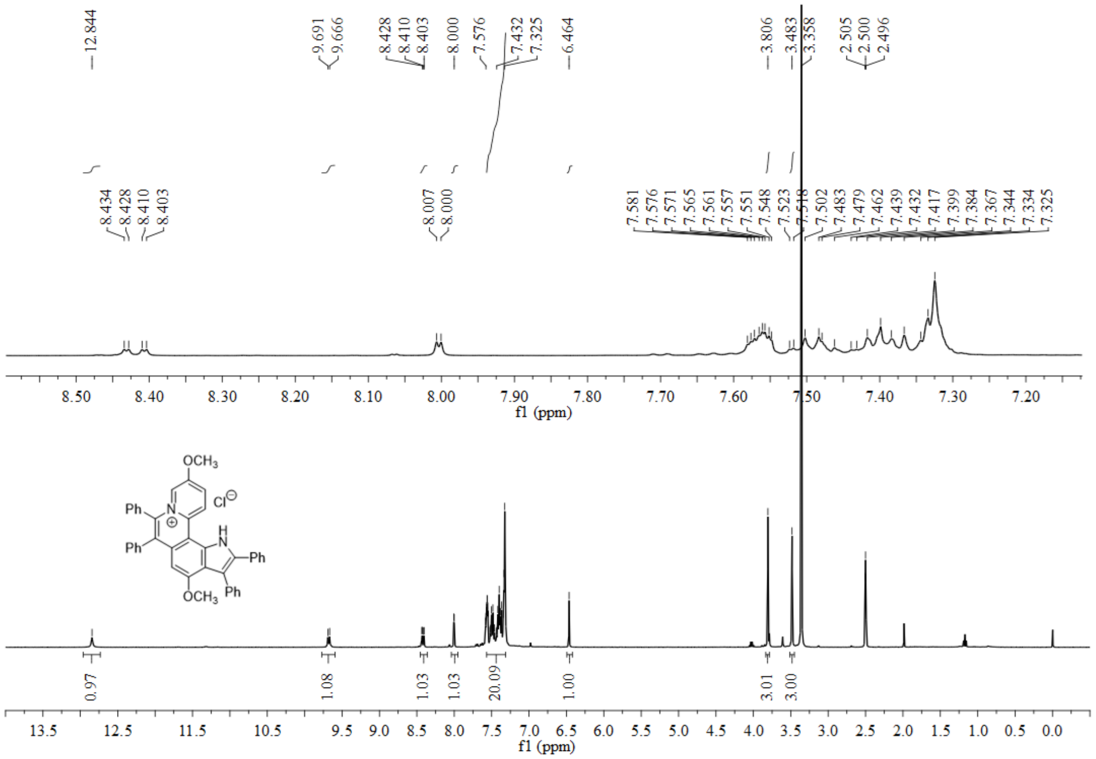
12.844
9.691
9.666
8.428
8.410
8.403
8.000
7.576
7.432
7.325
6.464
3.806
3.483
3.358
2.505
2.500
2.496
f1 (ppm)
OCH3
Cl
Ph
N
H
Ph
Ph
OCH3
Ph
0.97
1.08
1.03
1.03
20.09
1.00
3.01
3.00
f1 (ppm)

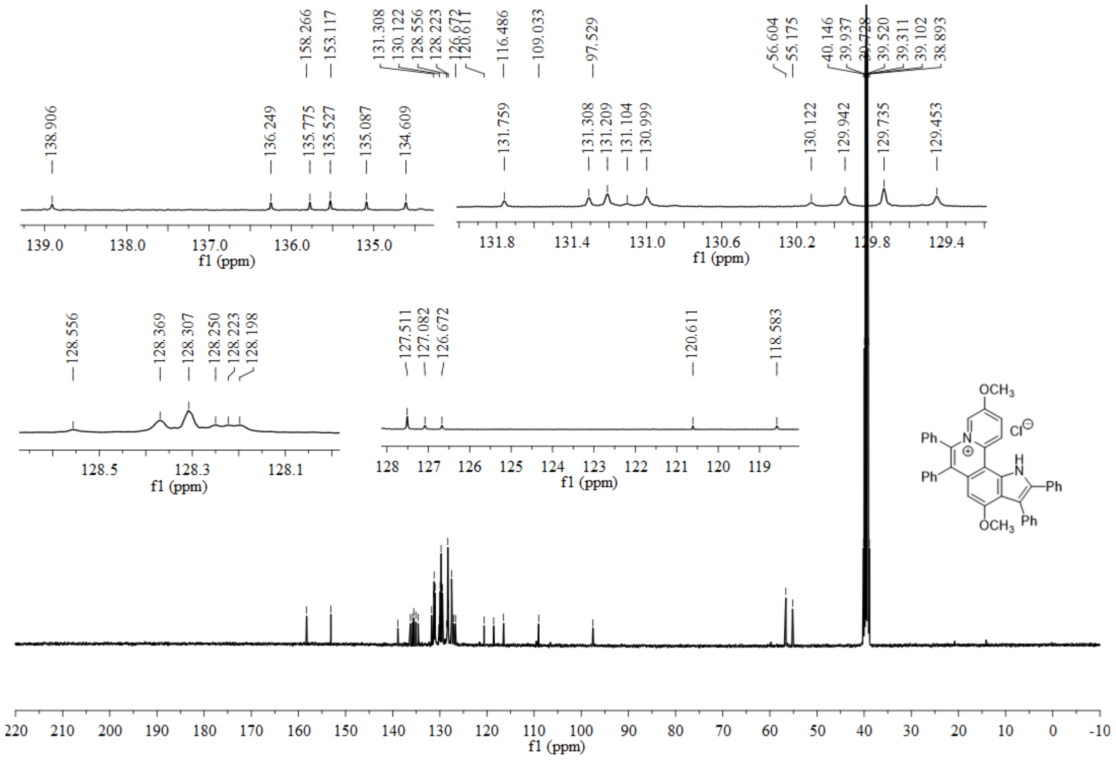
158.266
153.117
131.308
130.122
128.556
128.223
116.486
109.033
97.529
56.604
55.175
40.146
39.937
39.520
39.311
39.102
38.893
138.906
136.249
135.775
135.527
135.087
134.609
f1 (ppm)
131.759
131.308
131.209
131.104
130.999
130.122
129.942
129.735
129.453
f1 (ppm)
128.556
128.369
128.307
128.250
128.223
128.198
f1 (ppm)
127.511
127.082
126.672
120.611
118.583
f1 (ppm)
OCH3
Cl
Ph
N
H
Ph
Ph
OCH3
Ph
f1 (ppm)

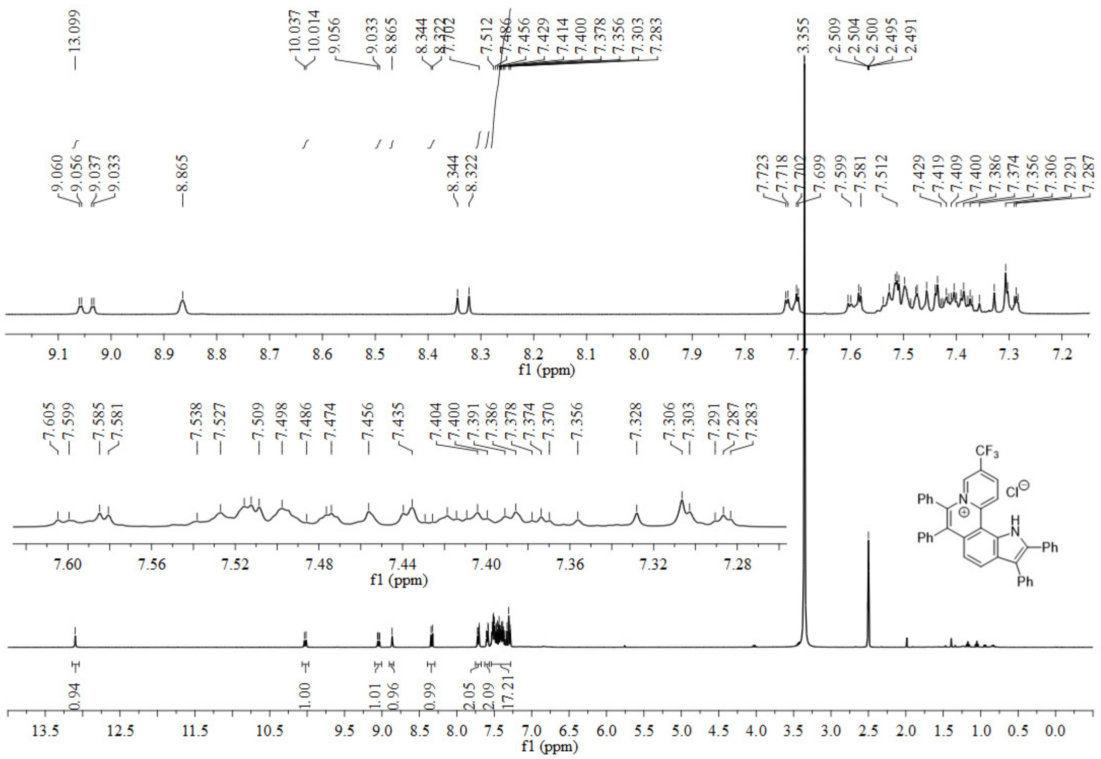

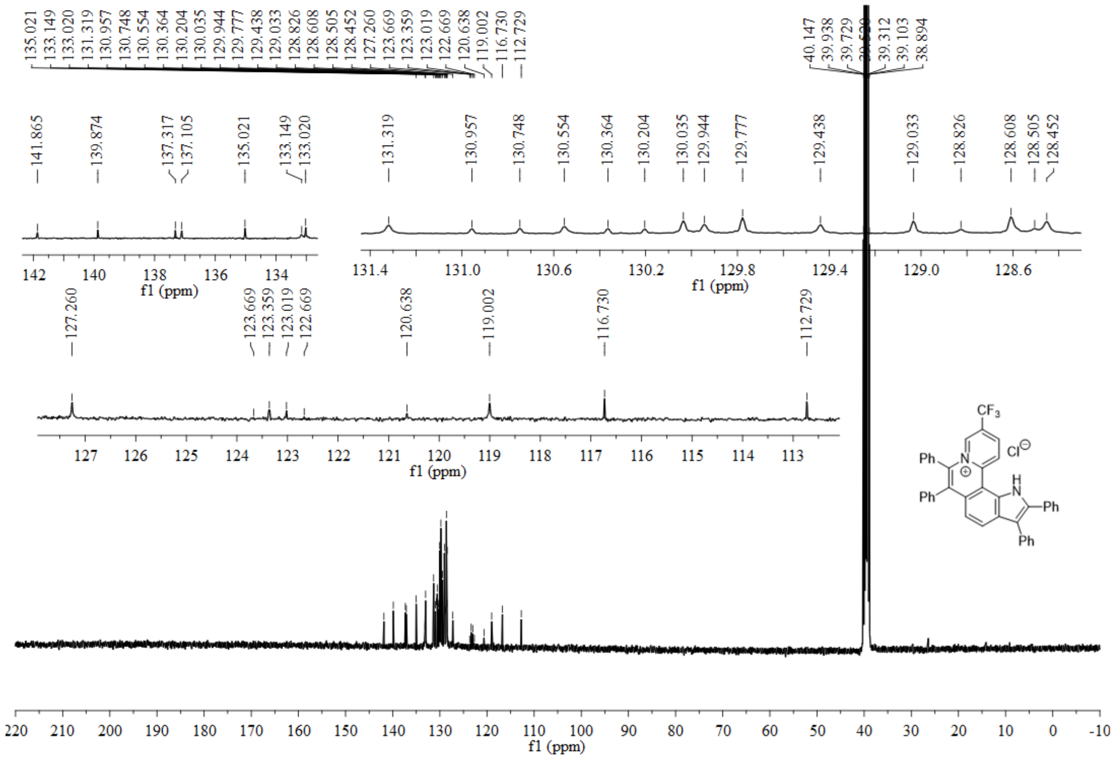

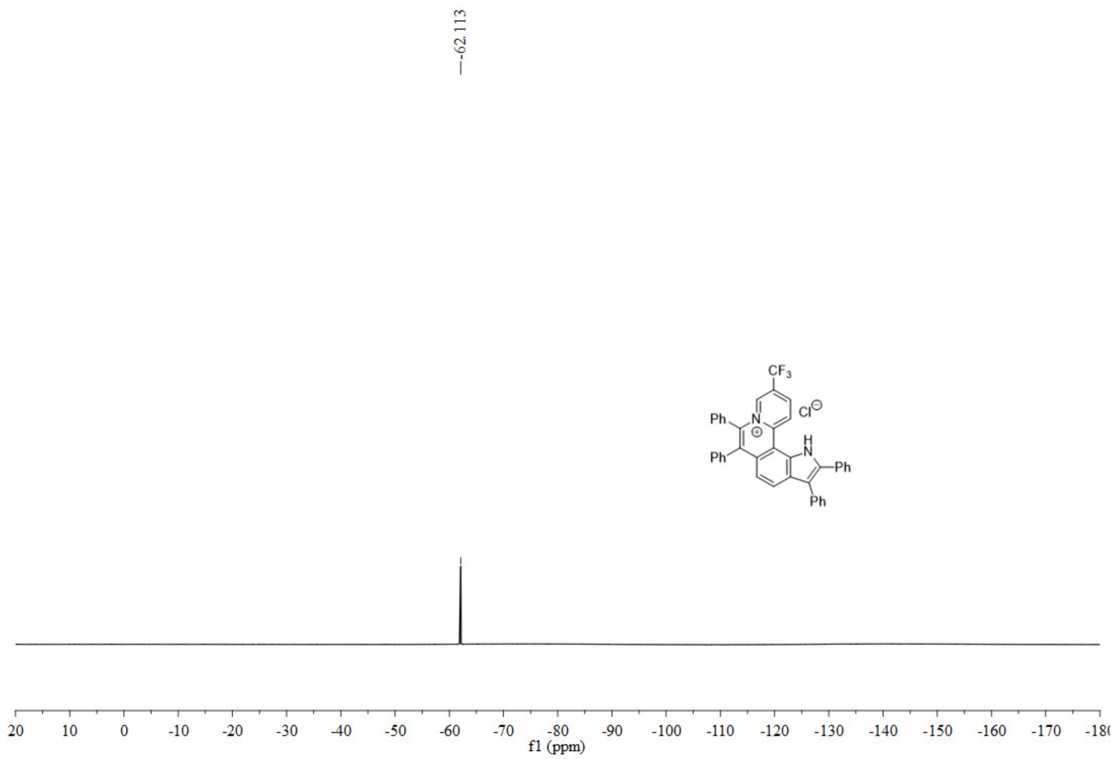
-62.113
CF3
Cl
Ph
Ph
N
H
N
Ph
Ph
20 10 0 -10 -20 -30 -40 -50 -60 -70 -80 -90 -100 -110 -120 -130 -140 -150 -160 -170 -18
f1 (ppm)

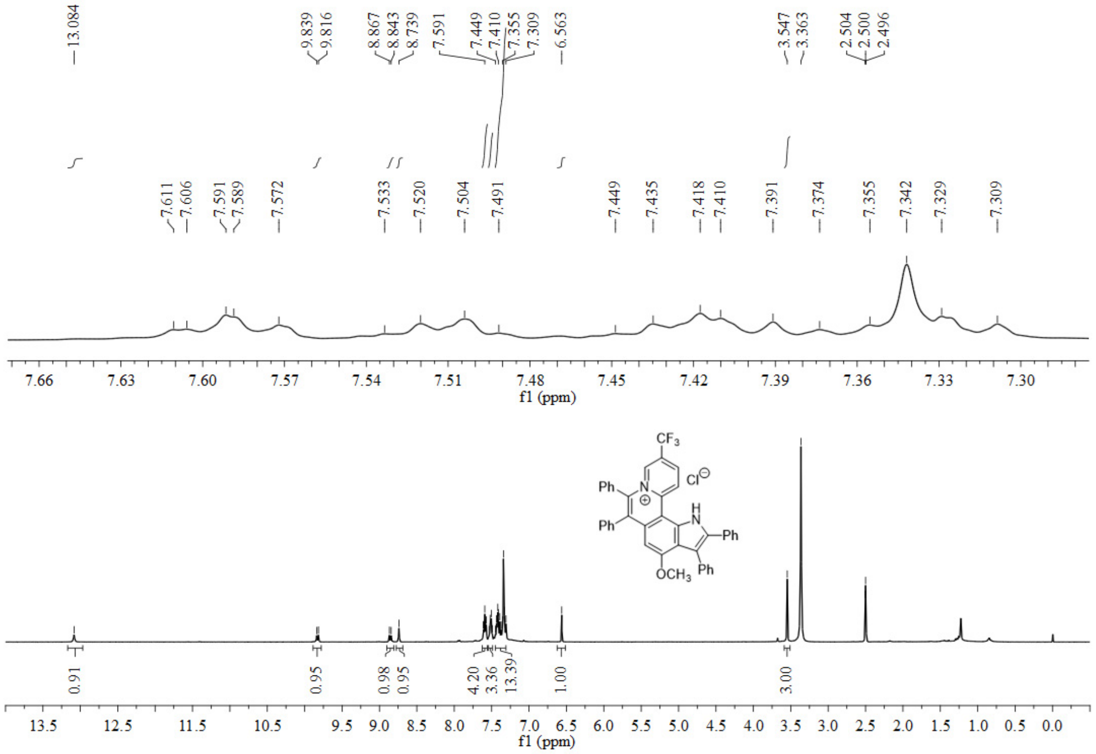
13.084
9.839
9.816
8.867
8.843
8.739
7.591
7.449
7.410
7.355
7.309
6.563
3.547
3.363
2.504
2.500
2.496
7.611
7.606
7.591
7.589
7.572
7.533
7.520
7.504
7.491
7.449
7.435
7.418
7.410
7.391
7.374
7.355
7.342
7.329
7.309
f1 (ppm)
CF3
Cl
Ph
N
H
OCH3
0.91
0.95
0.98
0.95
4.20
3.36
13.39
1.00
3.00
f1 (ppm)

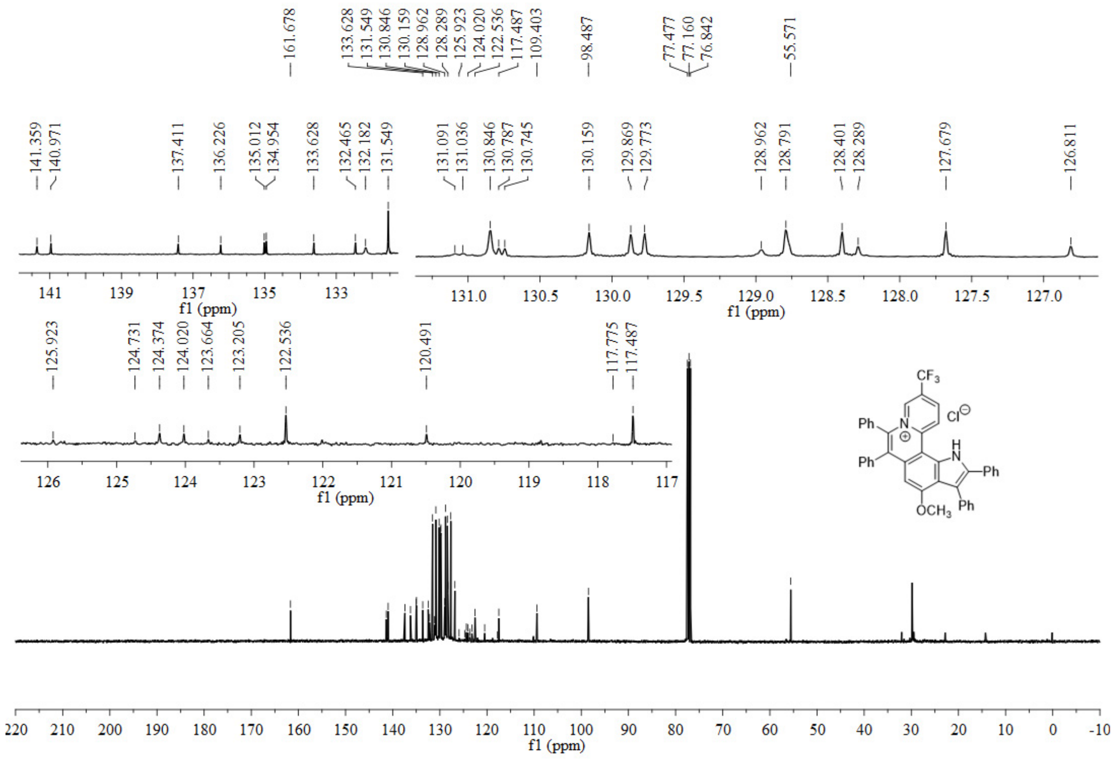
161.678
133.628
131.549
130.846
130.159
128.962
128.289
125.923
124.020
122.536
117.487
109.403
98.487
77.477
77.160
76.842
55.571
141.359
140.971
137.411
136.226
135.012
134.954
133.628
132.465
132.182
131.549
131.091
131.036
130.846
130.787
130.745
130.159
129.869
129.773
128.962
128.791
128.401
128.289
127.679
126.811
f1 (ppm)
f1 (ppm)
125.923
124.731
124.374
124.020
123.664
123.205
122.536
120.491
117.775
117.487
f1 (ppm)
CF3
Cl
Ph
N
H
OCH3
f1 (ppm)

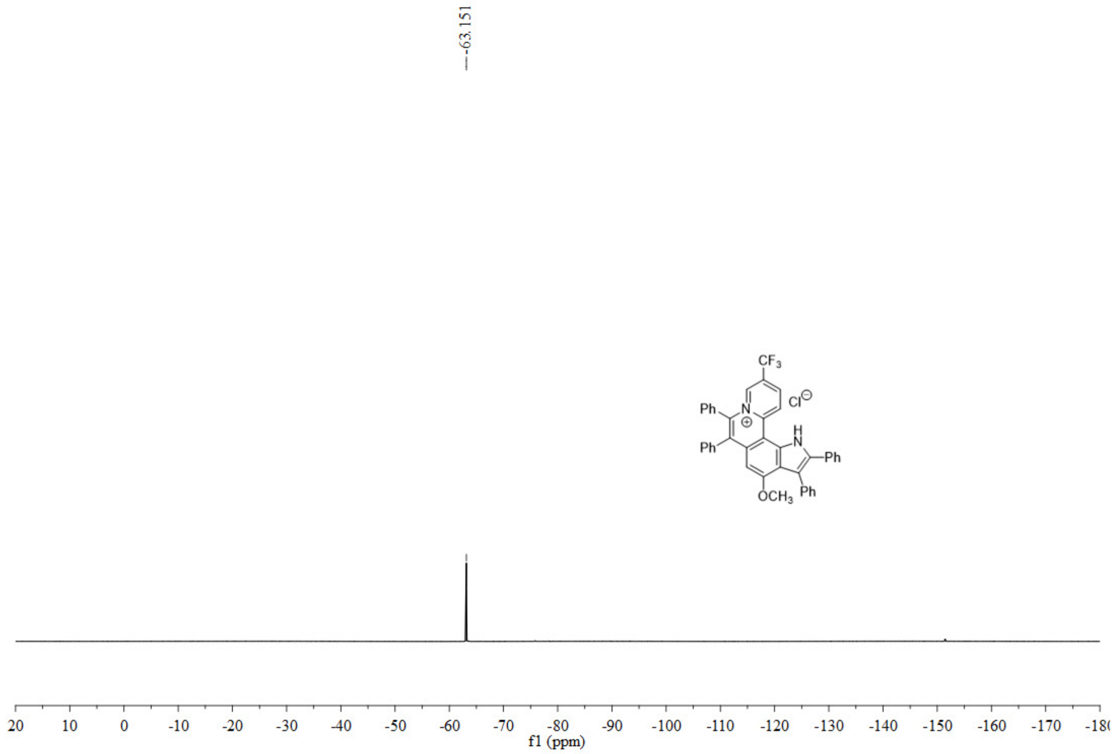
-63.151
CF3
Cl
Ph
N
Ph
H
N
Ph
OCH3
Ph
20 10 0 -10 -20 -30 -40 -50 -60 -70 -80 -90 -100 -110 -120 -130 -140 -150 -160 -170
f1 (ppm)

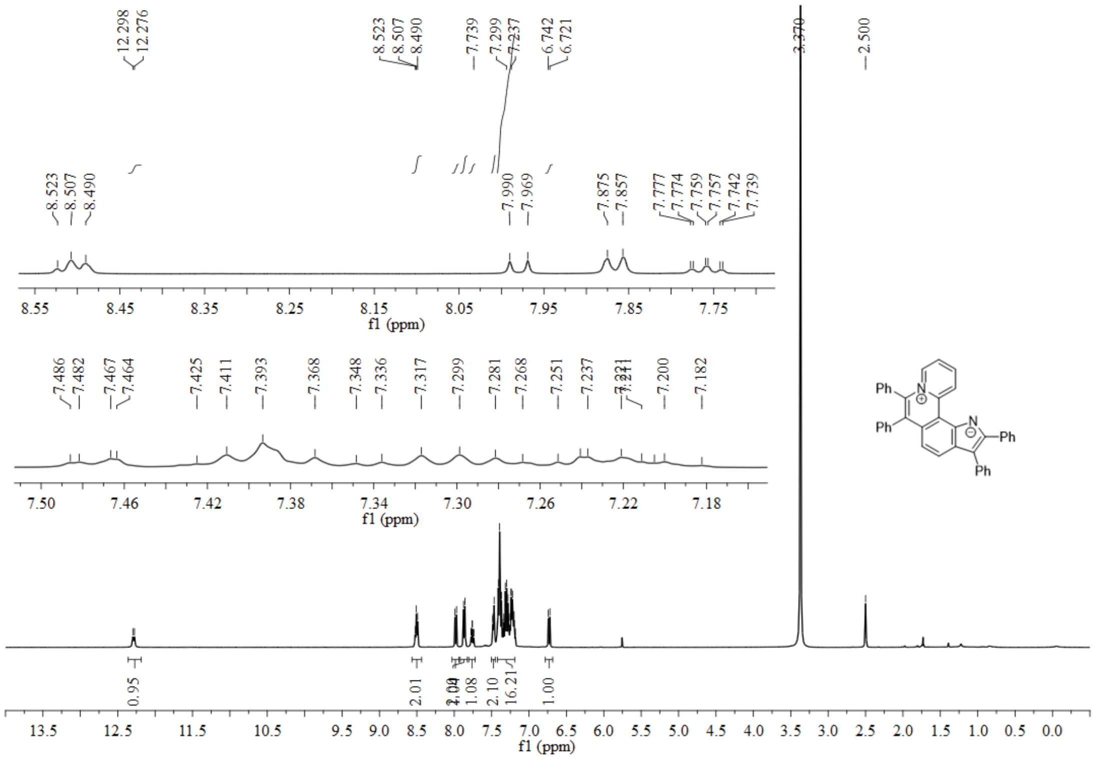

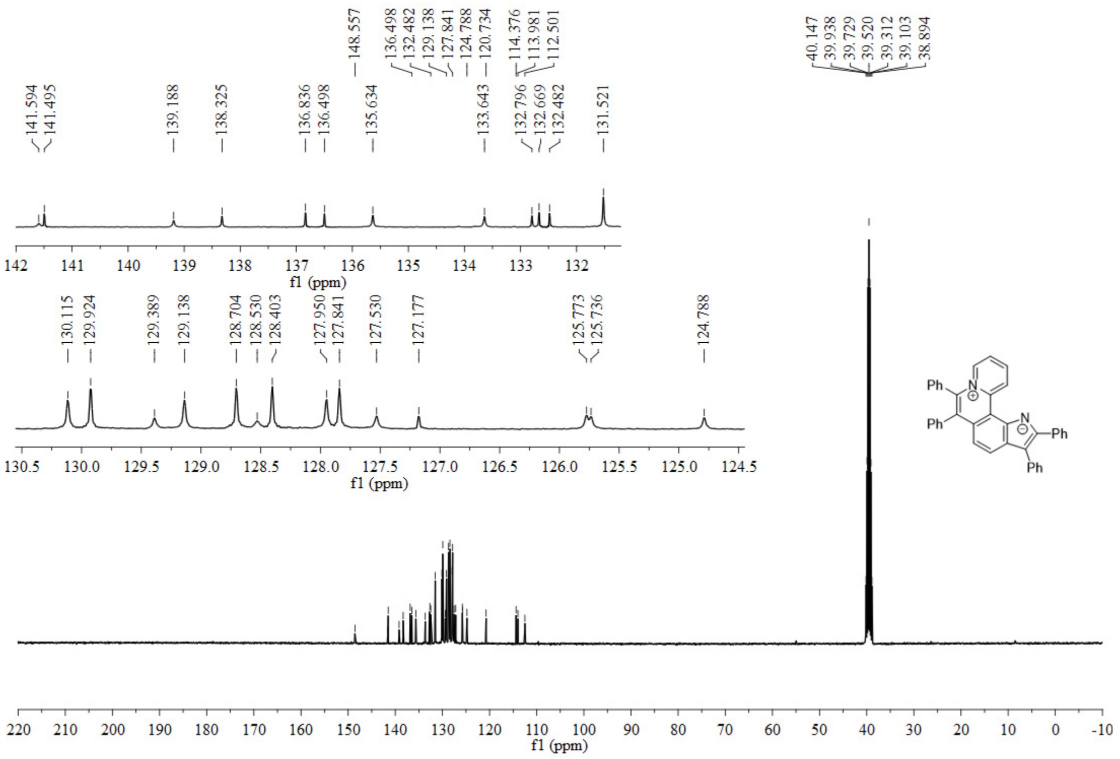

Supplement: SC-012-D0SC05844K-s001 [file SC-012-D0SC05844K-s001.pdf]
